# The comparative efficacy and safety of sweet solutions to reduce preterm infants’ pain levels: a systematic review and network meta-analysis

**DOI:** 10.1186/s13643-025-03043-3

**Published:** 2026-01-10

**Authors:** Defi Efendi, Yi-No Kang, Regina Natalia, Ariesta Milanti, Putri M. T. Marsubrin, Christina Yeni Kustanti, Kee-Hsin Chen

**Affiliations:** 1 School of Nursing, Taipei Medical University, Taipei, Taiwan; 2 Department of Pediatric Nursing, Faculty of Nursing, Universitas Indonesia, Depok, West Java Indonesia; 3 Neonatology Unit, Universitas Indonesia Hospital, Depok, West Java Indonesia; 4 Evidence-Based Medicine Center & Research Center of Big Data and Meta-Analysis, Wan Fang Hospital, Taipei Medical University, Taipei, Taiwan; 5 Cochrane Taiwan, Taipei Medical University, Taipei, Taiwan; 6 Institute of Health Policy and Management, College of Public Health, National Taiwan University, Taipei, Taiwan; 7 School of Nursing, Institut Kesehatan Mitra Bunda, Batam, Indonesia; 8 Strategic Delivery Unit, Ministry of Health, Jakarta, Indonesia; 9 Neonatology Division, Department of Child Health, Cipto Mangunkusumo National General Hospital, Faculty of Medicine Universitas Indonesia, Jakarta, Indonesia; 10 Study Program of Nursing Science, Sekolah Tinggi Ilmu Kesehatan Bethesda Yakkum, Yogyakarta, Indonesia; 11 Lotus Care, Private Clinic for Wound & Palliative Care, Homecare, Yogyakarta, Indonesia; 12 Post-Baccalaureate Program in Nursing, College of Nursing, Taipei Medical University, Taipei, Taiwan; 13 Department of Nursing & Research Center in Nursing Clinical Practice, Wan Fang Hospital, Taipei Medical University, Taipei, Taiwan

**Keywords:** Sweetener, Sucrose, Glucose, Breast milk, Calming effect, Physiological stability, Low birth weight, Newborn, Meta-regression

## Abstract

**Background:**

Sweet solutions are widely used to reduce procedural pain in preterm infants, but their comparative efficacy and safety remain unclear.

**Methods:**

We searched CINAHL, MEDLINE, Embase, CENTRAL, Scopus, and ProQuest for randomized controlled trials comparing glucose, sucrose, or expressed breast milk with control or with each other in preterm infants. We performed a random-effects frequentist network meta-analysis across three pain time points (reactivity, regulation, recovery). Pain level served as the primary outcome, while heart rate, oxygen saturation, respiratory rate, crying time, and adverse events were designated as secondary outcomes. Treatment efficacy was subsequently ranked using P-scores and a beading plot.

**Results:**

We screened 10,043 records, included 42 RCTs (2733 infants), and analyzed 38 RCTs (2367 infants) in the network meta-analysis. Compared to the controls alone, glucose (standardized mean difference [*SMD*], −0.72; 95% confidence interval [*CI*], −1.19 to −0.25) and sucrose (*SMD*, −0.56; 95% *CI*, −1.04 to −0.07) were associated with lower pain responses in the reaction phase. In the regulation and recovery phases, pain reduction was consistently linked to glucose, sucrose, and expressed breast milk. Those interventions were supported by results of P-scores that ranged from 0.877 to 0.917 showing glucose’s superiority in the three phases. Glucose was associated with a higher risk of adverse events. Half of the 38 trials in the network meta-analysis had a low risk of bias. The evidence certainty for the primary outcome was moderate to very low, while the certainty for the secondary outcomes spanned a range from high to very low.

**Conclusions:**

Glucose ranked most effective for reducing procedural pain in preterm infants, followed by sucrose and expressed breast milk. Future trials should evaluate optimal dosing, repeated administration, and combinations with other non-pharmacological pain-management strategies to maximize efficacy and safety.

**Systematic review registration:**

PROSPERO CRD42023389288

**Tweetable abstract:**

Glucose is the most effective sweet solution in alleviating pain scores in preterm infants, followed by sucrose and expressed breast milk.

**Supplementary Information:**

The online version contains supplementary material available at https://doi.org/10.1186/s13643-025-03043-3.

## Background

Compared to healthy-term neonates, preterm infants are more likely to experience prolonged and repetitive painful procedures [1, 58]. These unpleasant stimuli were reported to have negative clinical, physiological, and psychological consequences [22, 23, 29, 52]. Previous studies showed the impacts of painful exposures on brain development [20, 62], cognitive delays [8, 79, 81], and persistent internalizing behavioral problems across toddlerhood and age 8 years [56]. Furthermore, preterm infants experiencing pain are more likely to develop noncommunicable diseases [32, 42, 83]. Thus, measures to reduce pain sensations in this vulnerable population are urgently needed.

As a simple noninvasive method [35, 47, 76], the utilization of sweet solutions to reduce acute discomfort in neonates has garnered significant attention and investigation [35]. Previous systematic reviews and meta-analyses extensively examined the efficacy of glucose, sucrose, and breast milk [7, 19, 29, 35, 52, 53, 72, 75, 76], and these sweet solutions can reduce pain or crying times during various procedures such as heel lances, venipuncture, intramuscular injections, and eye examinations. However, the most effective sweet solutions intervention has yet to be definitively ascertained, and there is a notable absence of a comprehensive approach for evaluating the efficacy of diverse sweet solutions interventions within the contemporary scientific domain. Comparing more than two interventions using multiple pairwise meta-analyses may lead to incoherent estimates of the relative effects of every intervention compared to every other one [18].

In addition, several significant findings in previous syntheses relied on a fixed-effect model despite statistical heterogeneity concerns, whereas other syntheses aggregated data using a random-effects model. Comparing the effects of various sweet solutions’ interventions across these syntheses is inappropriate, especially given differences in analytical assumptions and statistical models.

A network meta-analysis (NMA) is a reliable method for synthesizing data from multiple treatment comparisons across trials [18, 65, 68]. For each outcome, it provides estimates of the effect for all possible pairwise comparisons and offers a comprehensive overview of the impact of sweet solution interventions on pain alleviation in preterm infants. This study aimed to rank sweet solution interventions for preterm infants undergoing painful procedures based on their efficacy and safety, using a consistent model through network meta-analysis [64].

## Methods

### Study design

A frequentist framework was used in this NMA. We adhered to the Preferred Reporting Items for Systematic reviews and Meta-analyses (PRISMA) with an NMA extension to report this study [43]. The protocol was registered in the PROSPERO International Prospective Register of Systematic Reviews (PROSPERO CRD42023389288).

### Search strategy and study selection

We searched relevant randomized controlled trials (RCTs) to compare different kinds of sweet solutions to reduce pain for various procedures in hospitals from six databases (CINAHL, MEDLINE, Embase, CENTRAL, Scopus, and ProQuest Dissertation and Thesis) starting from their inception to 26 December 2022. In addition, we use the automatic alert function of the database to track whether there are newly published studies until July 2024. Reference lists of published systematic reviews were also searched. Details of the search strategy sample are provided in Supplemental eTable 1.

Preterm infants who were less than 28 days old and experiencing procedural pain such as heel pricking, suctioning, orogastric/nasogastric insertion, or intravenous line cannulation were the target population in this study (Supplemental eTable 2) [33, 73]. Infants who were critically ill with altered consciousness or who had received analgesics were excluded from the study. Two researchers (A. M. and R. N.) independently screened titles and abstracts using EndNote 20 and assessed the full text for potential inclusion in the study. Any disagreement was resolved through a consensus moderated by the first author (D. E.).

### Intervention

This review incorporated three different sweet solutions. The following solutions containing sugar were defined as (1) glucose or dextrose as a single sugar’s sweetness from the monosaccharide class, (2) sucrose as a single sugar’s sweetness from the disaccharide class, (3) fructose as a single sugar’s sweetness from the monosaccharide class, and (4) expressed breast milk as a natural sweetener that contains sugar in the form of lactose and belongs to the disaccharide class [2, 25, 26]. However, fructose could not be analyzed in our synthesis due to data unavailability. Finally, we defined the control group as a placebo, no intervention, or other active intervention given above.

### Primary and secondary outcomes

We included pain as the primary outcome which was measured by validated instruments as described in Supplemental eTable 3. We modified previous definitions to categorize the pain level time measurement [15, 19, 22, 23], comprised of pain reactivity (during a procedure), pain regulation (immediately after a procedure), and pain recovery (more than 2 min after a procedure). Secondary outcomes comprising heart rate, respiratory rate, and oxygen saturation in the reactivity, regulation, and recovery phases were analyzed. In addition, we assessed the impacts of interventions on the total crying time and adverse events (AEs).

### Data extraction

D. E. and R. N. independently extracted data from the reviewed studies. Any discrepancies were resolved through consensus. We extracted characteristics of study participants such as gestational age, birth weight, gender, and other required data. We further extracted arm-level data including the sample size, mean, median, standard deviation, interquartile range, and other types of data for all of the given time measurements. Ten different scenarios were used to calculate missing data as described in a previous study [14].

### Quality of evidence and risk of bias

Two independent researchers (A. M. and R. N.) evaluated the risk of bias (RoB) among studies using the Cochrane risk of bias 2.0 [45, 74]. Additionally, confidence in the NMA (CINeMA) framework covering within-study bias, reporting bias, indirectness, imprecision, heterogeneity, and incoherence was used to assess the certainty of the evidence (CoE) [57]. This approach relies on the methodology proposed by the Grading of Recommendations Assessment, Development and Evaluation (GRADE) Working Group for pairwise meta-analyses (PMAs) [67].

### Data synthesis and statistical analysis

Transitivity assumptions were evaluated by checking distributions of potential confounding variables nested in the infants’ characteristics as described in Supplemental eTable 4. The DerSimonian–Laird random-effects model was used to calculate the contrast-based model meta-analysis [17, 30]. Cohen’s D was used to estimate and classify mean differences (MDs), standardized mean differences (SMDs) and risk ratios (RRs) between groups [21]. Some empirical studies have suggested that there may be minimal practical differences between frequentist and Bayesian approaches to network meta-analysis [51, 69]. Given that our confidence ratings are derived from CINeMA, which is grounded in frequentist principles [57], we opted to estimate relative treatment effects and rankings using a frequentist framework [82].

To illustrate treatment comparisons based on direct evidence, the network plot geometry was used for each outcome of interest [77]. The sizes of nodes in the plots corresponded to sample sizes, and the width of the edges was proportional to the total studies involved in the comparisons. Statistical analyses were performed in R, version 4.2.2, using the “netmeta” package [63, 78].

To summarize the findings with treatment rankings, we used P score that is analogous to the surface under the cumulative ranking curve (SUCRA) [34, 64, 66]. Then, we employed a beading plot to visually summarize the ranking probabilities for all outcomes of interest using the “rankinma” package [13]. This innovative graphic technique provides a concise representation of complex NMA rankings with multiple outcomes in a single linear plot. Each line corresponds to a specific outcome, and the position of the dots along the line indicates the relative ranking of treatments. In our meta-analysis, treatments positioned further to the right are considered more effective [12].

Heterogeneity, incoherence, and outliers within network models were evaluated. We assessed heterogeneity using *τ*^2^ by estimating the variance caused by the true effect size [38]. Values of *τ*^2^ of 0.04, 0.09, and 0.16 are respectively defined as low, moderate, and high heterogeneity [5]. Alternatively, *I*^2^ > 50% was also considered an attribute of higher between-study heterogeneity by assessing the amount of variance observed [40, 41]. We also examined the mean path length to estimate the degree of indirectness of an estimate [60] with a threshold of > 2, which indicated a less-reliable NMA estimate [48].

We used split direct and indirect evidence to detect the local consistency. Global inconsistency and a design-by-treatment interaction model were considered to detect inconsistencies within the entire NMA using Cochrane’s Q statistic with a significant level of *p* < 0.1 [39]. Meanwhile, Egger’s test and visual inspection of the comparison-adjusted funnel plots were used to evaluate publication bias and small study effect [11, 24].

Forward search algorithm was used to detect extreme study effects through “NMAoutlier” package [61]. Cook’s distance and the variance ratio were used to detect potential outliers. Furthermore, we created a forward plot with *z*-values to visually demonstrate whether the results were significantly impacted by each individual study, considering both direct and indirect evidence.

To explore the source of heterogeneity and inconsistency, we performed a sensitivity analysis, subgroup analysis, and network meta-regression. Sensitivity analysis in this study was carried out by removing a single trial with a high risk of bias and an extremely high dose of sweet solutions [16]. Subgroup analysis was performed to clarify the dose–range effect on pain intensity. In addition, a meta-regression was further performed to assess the impact of the risk of bias and type of procedure on the relative effect of treatment comparisons using R package “rjags” and “gemtc” with 10,000 simulation iterations. We explored whether the overall evidence differs from results based on low-bias studies and whether a single painful procedure, such as heel sticking, affects the overall outcomes. Because the analysis of adverse events involved rare events, the inverse variance method was considered susceptible to bias. Consequently, we confirmed our findings using the Peto and exact methods.

## Results

### Study characteristics

**Fig. 1:**
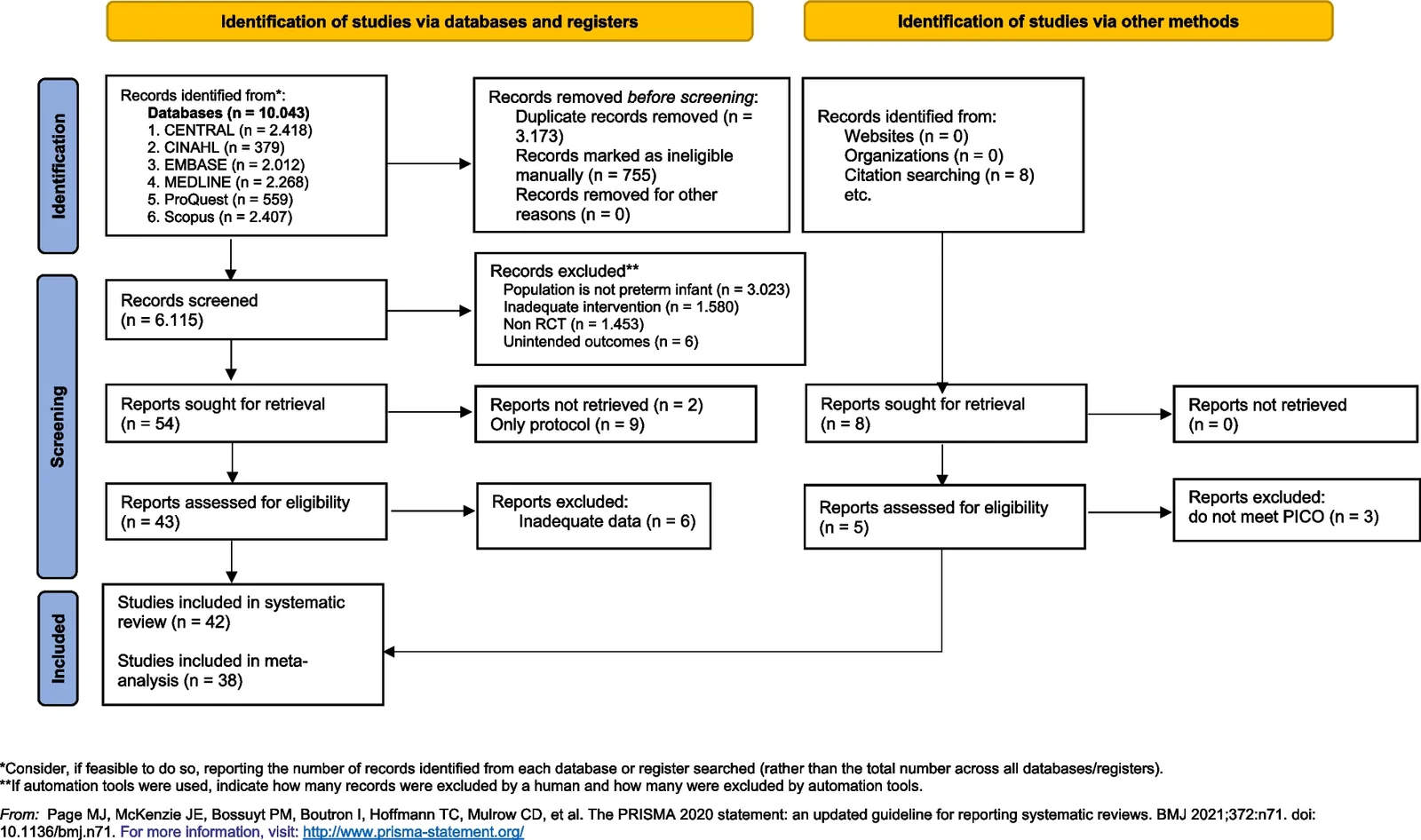
PRISMA 2020 flow diagram for comparative effectiveness among sweet solutions in preterm infants

The literature searches from 6 databases identified 10,043 potential references. After screening for duplicates and excluded studies, 52 full-text articles were assessed for eligibility. Six studies were excluded for reasons listed in Supplemental eTable 5. After incorporating studies from the previous review’s reference lists, 42 (2733 newborns) randomized controlled trials (RCTs) were eligible for the current synthesis, with 38 (2367 newborns) of these ultimately being analyzed in the network meta-analysis (Fig. 1).

The preterm infants included in the study had a mean gestational age of 32.16 weeks and an average birth weight of 1643.12 g. Our NMA was produced from three oral sweeteners consisting of dextrose, sucrose, and expressed breast milk. Most of the comparisons were constructed from direct evidence with a mean path length of < 2 (Supplemental eFig. 1), indicating the high reliability of the NMA estimate [48]. Characteristics of the included trials are described in Table 1. Half of the 38 trials revealed a low risk of overall bias. Ten studies (26.3%) demonstrated a moderate, and the last 9 studies (23.7%) were categorized as having a high RoB. Details of the RoB are reported in supplementary eFig. 2.

**Table 1:** Characteristics of the included studies

No	Author (year)	Country	Design	*N*	Gestational age (weeks)	Birth weight (g)	Procedures	Treatments	Control	Pain tools
1	Abad (1996)	Spain	Parallel	28	34.2 (2.1)	2015.3 (651.67)	Venipuncture	Sucrose 12% Sucrose 24%	Placebo	NA
2	Acharya (2004)	UK	Cross-over	39	30.5 (2.3)	NA	Venipuncture	Sucrose 25%	Placebo	NFCS
3	Axelin et al. (2009)	Finland	Cross-over	20	28.3 (2.3)	1123 (327)	Heel-stick, pharyngeal suctioning	Glucose 24%	Placebo	PIPP and NIPS
4	Belliene et al. (2003)	Italy	Cross-over	17	31.32 (1.11)	NA	Heel-stick	Glucose 10%	NT	NA
5	Bellieni (2001)	Italy	Cross-over	17	NA	NA	Heel-stick	Glucose 10%	Placebo	PIPP
6	Boyer (2004)	Canada	Parallel	41	27.85 (1.75)^a^	NA	Heel-stick, venipuncture and suctioning	Sucrose 24%	Placebo	NA
7	Boyle (2006)	Canada	Parallel	20	NA	NA	ROP screening	Sucrose 33%	Placebo	PIPP
8	Bucher (1995)	Switzerland	Parallel	16	NA	NA	Heel-stick	Sucrose 50%	Placebo	NA
9	Bueno (2012)	Brazil	Parallel	113	35.7 (0.7)	2348.1 (469.5)	Heel-stick	EBM	Glucose 25%	PIPP
10	Carbajal (2002)	France	Cross-over	24	28.1 (1.42)	1036 (153.67)	Subcutaneous injections	Glucose 30%	Placebo	DAN Scale
11	Cirik (2020)	Turkey	Parallel	64	33.18 (0.84)	2048.9 (380.57)^b^	OGT insertion	EBM	NT	PIPP
12	Da Costa (2013)	Brazil	Parallel	124	30.2 (1.95)	1257.4 (274.35)^b^	ROP screening	Glucose 25%	NT	NIPS
13	Dehghani (2019)	Iran	Parallel	66	35.48 (0.64)	1952.7 (385.44)^b^	Phlebotomy	Glucose 50%	NT	NIPS
14	Desai (2017)	India	Parallel	72	32.84 (3.71)	1394.5 (611.41)^b^	Suctioning	EBM	Sucrose (NA)	PIPP
15	Deshmukh (2002)	India	Parallel	60	34.27 (1.47)	1470.5 (235.83)	Venipuncture	Glucose 10% Glucose 25%	Placebo	
16	Elserafy (2009)	Saudi Arabia	Parallel	36	32.4 (2.9)^a^	1700 (600)	Heel-stick and/or venipuncture	Sucrose 24%	Placebo	PIPP
17	Freire (2008)	Brazil	Parallel	64	234.9 (10.06)^a^	1514.2 (317.83)^b^	Heel-stick	Glucose 25%	NT	PIPP
18	Gaspardo (2008)	Brazil	Parallel	33	30.18 (1.97)	1066.7 (203.36)	Venipuncture, heel-stick, and other minor procedures	Sucrose 25%	Placebo	NFCS
19	Hsieh (2017)	Taiwan	Cross-over	20	31.97 (2.49)	1597.3 (446.68)	Heel-stick	Dextrose 10% EBM	Placebo	PIPP
20	Johnston (1997)	Canada	Parallel	47	31.4 (2.9)	1563 (496)	Heel-stick	Sucrose 24%	Placebo	NFCS
21	Johnston (1999)	Canada	Parallel	48	30.5 (2.23)	1482 (425)^b^	Heel-stick	Sucrose 24%	Placebo	PIPP
22	Kazmi (2020)	Pakistan	Parallel	400	NA	2930 (490)	Venipuncture	Glucose 25%	EBM	PIPP
23	Kristoffersen (2011)	Sweden	Cross-over	24	32.14 (1.37)^a^	4644 (356.98)^b^	OGT insertion	Sucrose 30%	Placebo	PIPP
24	Kumari (2016)	India	Parallel	94	35.55 (0.76)	NA	Heel-stick	Glucose 25%	Sucrose 24%	PIPP
25	McCullough (2007)	UK	Parallel	51	NA	1690 (370)^b^	OGT insertion	Sucrose 24%	Placebo	NFCS
26	Naik (2021)	India	Parallel	64	33.31 (1.96)	1455 (320)	ROP examination	EBM	NT	PIPP
27	Okan (2007)	Turkey	Cross-over	90	30.5 (2.7)	1401 (406)	Heel-stick	Sucrose 20% Glucose 20%	Placebo	NFCS
28	Olsson (2010)	Sweden	Parallel	29	28.4 (2.3)	1126.5 (355.5)	ROP screening	Glucose 30%	Placebo	PIPP
29	Ou Yang (2012)	Taiwan	Parallel	123	34.27 (1.45)	2031 (372)	Heel-stick	Glucose 25%	Placebo	N-PASS
30	Pandey (2013)	India	Parallel	105	33.5 (1.65)	1624.6 (371.55)	OGT insertion	Sucrose 24%	Placebo	PIPP
31	Ramar (2019)	India	Parallel	22	32 (2)	1344.8 (418.5)	ROP screening	Glucose 25%	Placebo	PIPP
32	Ramenghi (1996)	UK	Cross-over	15	32.48 (1.4)	1968 (347.82)	Heel-stick	Sucrose 25%	Placebo	NFCS
33	Ramenghi (1999)	UK	Cross-over	30	NA	NA	Heel-stick	Sucrose 25%	Placebo	NA
34	Ranjbar (2020)	Iran	Cross-over	46	32.35 (2.81)	2173.5 (413.47)	Heel-stick	Glucose 50%	NT	PIPP
35	Rawal (2018)	India	Parallel	67	35.19 (0.84)	2136.7 (390)	Heel-stick	Glucose 25% EBM	Placebo	PIPP
36	Rodrigues (2017)	India	Parallel	40	31.58 (1.89)	1448 (385.78)	Nasopharyngeal suctioning	Glucose 25%	EBM	PIPP
37	Sagheb (2020)	Iran	Parallel	20	29.06 (2.21)	1168.5 (266.34)	ROP screening	Glucose 25%	Placebo	PIPP
38	Sasidharan (2022)	India	Parallel	64	32.7 (1.75)	1623.5 (401)	Heel-stick	Glucose 25%	Sucrose 24%	PIPP
39	Taplak (2017)	Turkey	Parallel	60	NA	NA	ROP screening	Sucrose 33% EBM	Placebo	PIPP
40	Tekgunduz (2019)	Turkey	Parallel	71	30.42 (3.51)	1522 (682.49)	NCPAP	Glucose 30%	NT	PIPP
41	Uzelli & Yapucu (2015)	Turkey	Parallel	80	32.55 (2.8)	2220 (500)	IM Injection	Glucose 5%	NT	NIPS
42	Vezyroglou (2014)	Germany	Cross-over	16	34 (1.85)	2083.5 (476.5)	Oropharyngeal suctioning	Glucose 20%	Placebo	PIPP

*Abbreviations and notes*: *NFCS* neonatal facial coding system, *DAN scale* Douleur Aigue ¨ Nouveau-ne ´ scale, *PIPP* Preterm Infant Pain Profile, *NIPS* Neonatal Infant Pain Scale, *N-PASS* Neonatal Pain, Agitation and Sedation Scale, *NT* no treatment, *NA* not available, *EBM* expressed breast milk, *OGT* orogastric tube, *NCPAP* nasal continuous positive airway pressure, *RoP* retinopathy of prematurity, *IM* intramuscular

^a^ Post-menstrual age

^b^ Current weight

### Primary outcome: pain

**Fig. 2:**
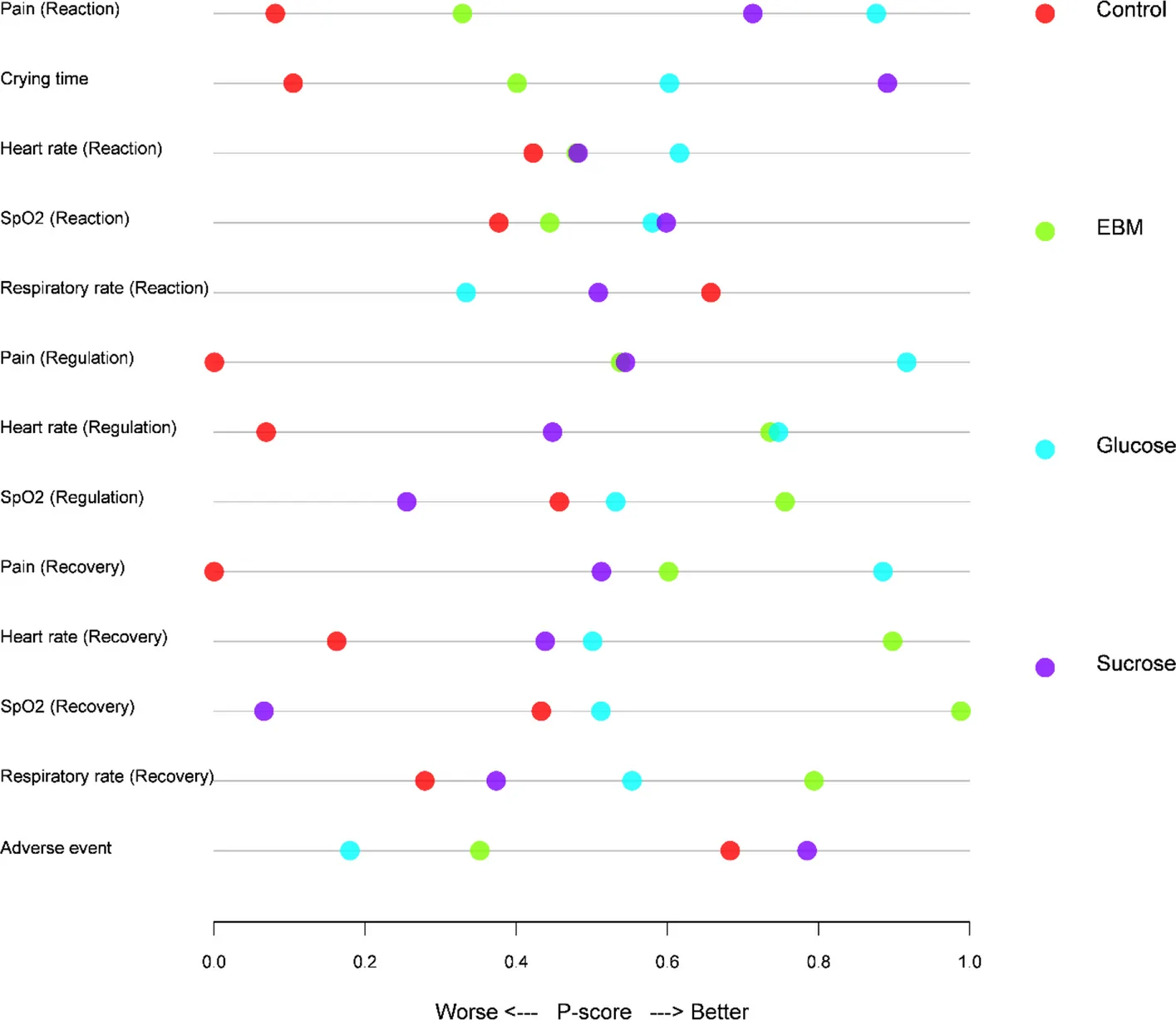
Beading plot using P scores. This figure describes how the ranking probabilities (based on P scores) were distributed across the outcomes of interest. In general, the control groups more likely congregated on the left side of the plot, showing their inferiority compared to the others. Meanwhile, glucose appears to dominate the right area of ​​the picture

Pain levels were assessed using several pain measurement instruments, including the Premature Infant Pain Profile (PIPP), PIPP-R (Premature Infant Pain Profile-Revised), Douleur Aiguë Nouveau-né (DAN; Newborn Acute Pain) Scale, Neonatal/Infant Pain Scale (NIPS), and Neonatal Facial Coding Scale (NFCS). In the reactivity phase, glucose and sucrose mostly showed better clinical outcomes, as depicted in Fig. 2.

**Fig. 3:**
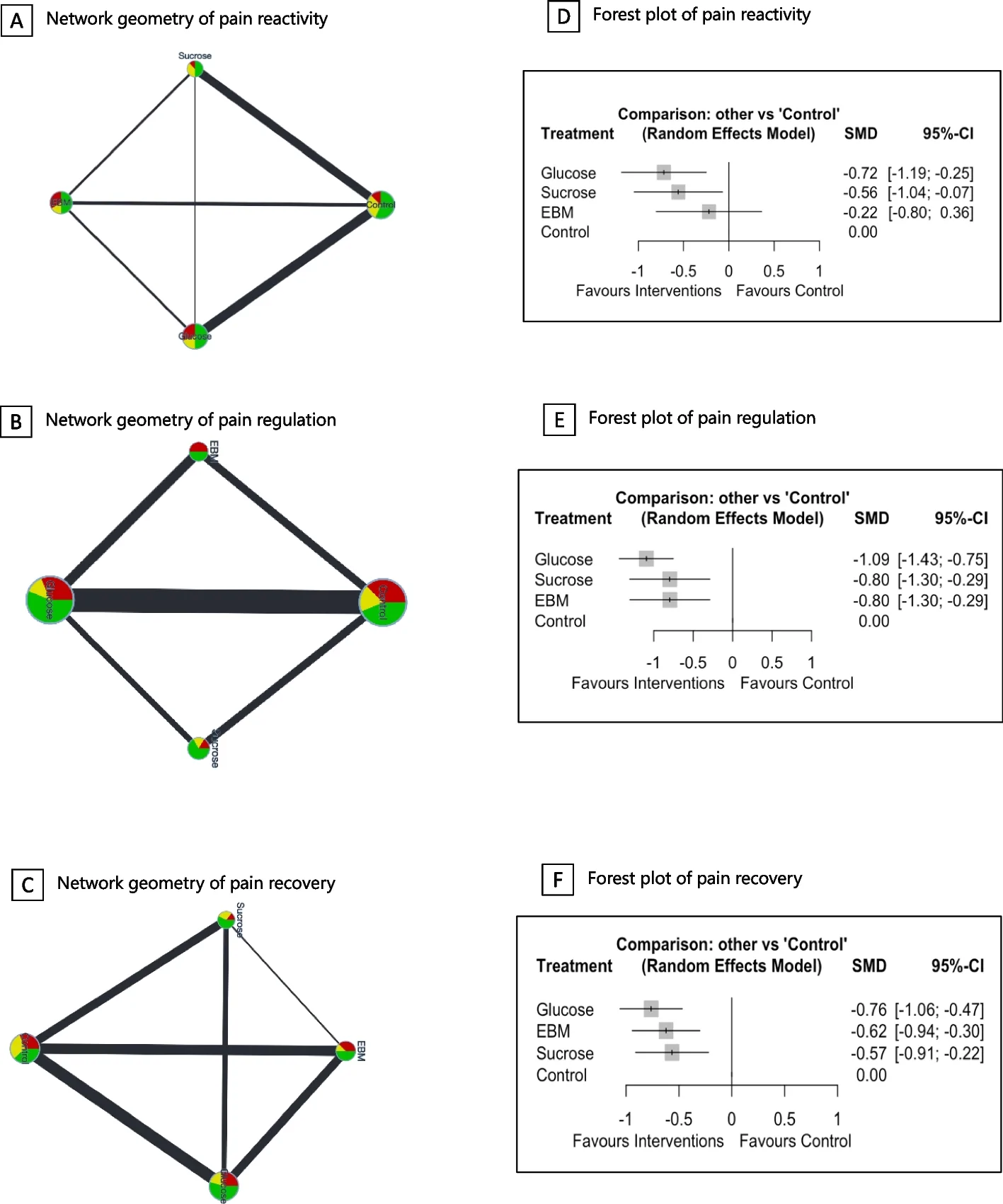
Network geometry, and forest plot of direct comparisons among sweet solutions. Pictures **A**, **B**, and **C** depict the network geometry of pain interventions in the reaction (23 studies, 4 nodes with 1533 participants), regulation (28 studies, 4 nodes with 1669 participants), and recovery stages (17 studies, 4 nodes with 1563 participants). The sizes and colors in the node respectively correspond to the number of subjects and risk of bias (RoB). The thickness of the lines indicates the total number of studies in direct comparisons. Pictures **D**, **E**, and **F** show effect sizes of sweet solutions with the 95% confidence interval (CI). A random-effects meta-analysis was conducted to estimate treatment effects. Glucose demonstrated the most significant reduction in pain regulation (SMD = -1.09, 95% CI: -1.43 to -0.75), followed by sucrose and EBM (both SMD = -0.80, 95% CI: -1.30 to -0.29). For pain recovery, glucose (SMD = -0.76, 95% CI: -1.06 to -0.47) and EBM (SMD = -0.62, 95% CI: -0.94 to -0.30) were more effective than control. These findings highlight the superior efficacy of glucose in managing pain across all domains, with sucrose and EBM offering additional benefits, particularly for pain recovery and regulation

Figure 3 show network and forest plots of direct comparisons of sweet solutions as analgesia. A total of 23 studies involving 1553 infants were investigated for pain reactions (Supplemental eTable 6). Compared to the controls alone, glucose (standardized mean difference [*SMD*], −0.72; 95% confidence interval [*CI*], −1.19 to −0.25) and sucrose (−0.56; 95% *CI*, −1.04 to −0.07) were associated with lower pain responses in the reaction phase. In the regulation phase, glucose (*SMD*, −1.09; 95% *CI*, −1.43 to −0.75), sucrose (*SMD*, −0.80; 95% *CI*, −1.30 to −0.29), and expressed breast milk (*SMD*, −0.80; 95% *CI*, −1.30 to −0.29) were likely to reduce pain compared to the controls. Thirdly, compared to the controls, glucose (*SMD*, −0.76; 95% *CI*, −1.06 to −0.47), sucrose (*SMD*, −0.62; 95% *CI*, −0.94 to −0.30), and expressed breast milk (*SMD*, −0.57; 95% *CI*, −0.91 to −0.22) were likely to reduce pain during the recovery phase. A summary of the NMA is given in Table 2. These interventions were further supported by P-scores that ranged from 0.877 to 0.917, showing glucose’s superiority in the three phases (Supplemental eTable 7).

**Table 2:** Summary of network meta-analysis for comparative efficacy and safety of glucose, sucrose, and expressed breast milk

Outcomes	Glucose vs. controls	Sucrose vs. controls	EBM vs. controls	Glucose vs. sucrose	Glucose vs. EBM	Sucrose vs. EBM
Pain						
Reaction^‡^	−0.72 [−1.19, −0.25]	−0.56 [−1.04, −0.07]	−0.22 [−0.80, 0.36]	−0.16 [−0.78, 0.47]	−0.50 [−1.13, 0.13]	−0.34 [−1.00, 0.32]
Regulation^‡^	−1.09 [−1.43, −0.75]	−0.80 [−1.30, −0.29]	−0.80 [−1.30, −0.29]	−0.30 [−0.82, 0.23]	−0.30 [−0.79, 0.19]	−0.00 [−0.68, 0.67]
Recovery^‡^	−0.76 [−1.06, −0.47]	−0.62 [−0.94, −0.30]	−0.57 [−0.91, −0.22]	−0.20 [−0.56, 0.17]	−0.14 [−0.47, 0.19]	0.06 [0.47, −0.36]
Crying time	−12.31 [−21.32, −3.31]	−19.53 [−30.76, −8.30]	−6.60 [−32.75, 19.55]	7.22 [20.46, −6.03]	−5.71 [−31.25, 19.84]	−12.92 [−40.99, 15.15]
Heart rate						
Reaction^¥^	−0.99 [−6.10, 4.12]	−0.16 [−1.98, 1.67]	−0.15 [−6.56, 6.25]	−0.83 [−6.08, 4.42]	−0.84 [−9.02, 7.34]	−0.01 [−6.61, 6.60]
Regulation^¥^	−6.56 [−14.90, 1.77]	−3.10 [−7.67, 1.46]	−6.50 [−14.45, 1.45]	−3.46 [−11.58, 4.66]	−0.06 [−11.58, 11.45]	3.40 [12.57, −5.77]
Recovery^¥^	−2.07 [−7.14, 2.99]	−1.64 [−6.53, 3.26]	−5.23 [−10.23, −0.23]	−0.44 [−6.57, 5.69]	3.16 [9.35, −3.04]	3.59 [9.76, −2.57]
Oxygen satuation						
Reaction^¥^	0.40 [−1.50, 2.30]	0.41 [−1.16, 1.99]	0.05 [−2.61, 2.70]	0.01 [−2.31, 2.34]	0.35 [−2.90, 3.61]	0.37 [−2.60, 3.34]
Regulation^¥^	0.24 [−2.54, 3.03]	−0.75 [2.00, −3.49]	1.50 [−2.60, 5.60]	0.99 [−2.39, 4.37]	−1.26 [3.70, −6.21]	−2.25 [2.69, −7.18]
Recovery^¥^	0.16 [−1.00, 1.32]	−0.94 [0.48, −2.36]	1.72 [0.45, 2.99]	1.11 [−0.58, 2.79]	−1.56 [0.04, −3.15]	−2.66 [−0.81, −4.52]
Respiratory rate						
Reaction^¥^	1.98 [8.62, −4.65]	0.81 [7.46, −5.84]	NA	1.17 [9.30, −6.96]	NA	NA
Recovery^¥^	−1.33 [−4.88, 2.22]	−0.22 [−5.91, 5.47]	−2.93 [−8.15, 2.29]	−1.10 [−7.06, 4.85]	1.60 [6.25, −3.05]	2.71 [9.97, −4.55]
Adverse events^$^	1.55 [1.09, 2.19]	0.83 [0.36, 1.92]	1.47 [0.44, 4.89]	1.86 [0.76, 4.54]	1.05 [0.33, 3.35]	0.56 [0.13, 2.42]

For pain, a negative standardized mean difference (SMD) indicates that the intervention favored reducing pain compared to the control. Similarly, for crying time, heart rate, respiratory rate, and adverse events, a negative MD or an RR <1 indicates that the intervention was beneficial. Conversely, for oxygen saturation, a positive MD reflects a favorable outcome for the intervention. *NA* not available. ‡Standardized mean difference (SMD). ¥Mean difference (MD). $Risk ratio (RR). *EBM* expressed breast milk, *VS* comparison

### Secondary outcomes

#### Crying time

Crying times were reported in 13 trials involving 731 preterm infants, across 4 treatment groups (Supplemental eFig. 3, eTable 6). Among groups, both glucose (*MD*, −12.31; 95% *CI*, −21.32 to −3.31) and sucrose (*MD*, −19.53; 95% *CI*, −30.76 to −8.30) showed significantly lower total crying times (Supplemental eFig. 4). Meanwhile, expressed breast milk did not show a statistically significant effect of reducing crying times compared to the controls (*MD*, −6.60; 95% *CI*, −32.75 to 19.55). P-scores indicated that sucrose and glucose were superior to the other groups (Supplemental eTable 7).

#### Heart rate

Fifteen studies reported heart rate in the three phases. Most of the comparisons showed that there was no evidence of efficacy. Compared to the controls, only expressed breast milk in the recovery state (number of studies = 3, *n* = 186) was associated with a statistically significant HR reduction (*MD*, −5.23; 95% *CI*, −10.23 to −0.23) (Table 2, Supplemental eTable 6). P-scores also revealed the highest probability for expressed breast milk among the others at 0.8981 (Supplemental eTable 7).

#### Respiratory rate

Table 2 shows that there was no efficacy of the interventions in reducing the respiratory rate in the reaction and recovery stages. The NMA was not performed in the regulation stage due to an inadequate number of studies.

#### Oxygen saturation

Most of the treatment comparisons did not show a statistically significant effect on oxygen saturation in any phase. Compared to the controls (13, *n* = 741), expressed breast milk and sucrose were associated with higher oxygen saturation levels (*MD*, 1.72; 95% *CI*, 0.45 to 2.99) and (MD, 2.66; 95% *CI*, 0.81 to 4.52). P-scores indicated that expressed breast milk was the most effective treatment (0.9886; Supplemental eTable 7).

#### Adverse events (AEs)

Fifteen studies (*n* = 1107) reported one or more AEs through all phases, consisting of desaturation, bradycardia, regurgitation, or vomiting. AEs were significantly more frequent with glucose (60/337) than with control (40/248), with a 55% higher risk of adverse events (*RR* 1.55; 95% *CI* 1.09 to 2.19). Sucrose showed a slightly lower adverse-event rate (8/157) than control, but the effect was not statistically significant (*RR* 0.83; 95% *CI* 0.36 to 1.92). Expressed breast milk also did not differ clearly from control (5/77), with an imprecise estimate and wide confidence interval (*RR* 1.47; 95% *CI* 0.44 to 4.89), reflecting limited data.

### Evaluation of heterogeneity, inconsistency, outlier, and small-study effect

Across outcomes, heterogeneity varied substantially. The primary pain outcomes showed considerable heterogeneity (*τ*^2^ = 0.13–0.42; *I*^2^ = 63.1%–84.6%), and heterogeneity was very high for crying time (*τ*^2^ = 99.34; *I*^2^ = 92.8%). In contrast, physiologic outcomes were generally low-to-moderately heterogeneous (e.g., heart rate: *τ*^2^ = 0–7.34; *I*^2^ = 0%–27.1%; respiratory rate during *τ*^2^ = 13.38; *I*^2^ = 41.9%), with some domains showing marked variability (e.g., oxygen saturation regulation: *τ*^2^ = 3.47; *I*^2^ = 81.5%). For adverse events, heterogeneity was negligible (*τ*^2^ = 0; I^2^ = 0%) (Supplemental eTable 8).

Our NMA showed no local inconsistencies (Supplemental eTable 9). Some inconsistencies were detected in the global assessment and design by treatment, but most of them were resolved after removing studies in the sensitivity analysis (Supplemental eTables 10, 11). Furthermore, Cook’s distance (Supplemental eFig. 5) and ratio of variance (Supplemental eFig. 6) concurrently indicated four outliers in the model of primary outcome. However, these extreme values did not seriously affect the findings. They were only weakly linked to the differences between direct and indirect evidence without statistical significance (absolute *z*-values < 2, Supplemental eFig. 7), and the treatment rankings based on global metrics remained largely stable (Supplemental eFig. 8). Comparison-adjusted funnel plots did not show obvious asymmetry (Supplementary eFig. 9).

### Sensitivity analyses, subgroup analysis, and meta-regression

Glucose maintained its superiority in relieving pain in the sensitivity analysis. A summary of the sensitivity analysis is presented in Supplemental eTable 12. Subgroup analysis by dose category identified dose strength as a potential source of heterogeneity and inconsistency. The low-dose subgroup (*n* = 9) exhibited moderate heterogeneity (*τ*^2^ = 0.3381; *I*^2^ = 60.3%), while the high-dose subgroup (*n* = 9) showed substantially higher heterogeneity (*τ*^2^ = 1.0429; *I*^2^ = 91.1%). This was supported by Cochran’s Q test for inconsistency (low dose: *p* = 0.014; high dose: *p* < 0.0001). The meta-regression analysis showed that high risk of bias was a source of heterogeneity in the consistency model of pain during the reaction phase (*β* = −1.87, *CrI* −3.35 to −0.46), but trends and treatment ranks were not affected by the risk of bias (Supplemental eFig. 10). On the other hand, painful procedure (i.e., heel sticking) seemed to be not a critical source of heterogeneity on pain during the reaction phase (*β* = −0.716, *CrI* −1.88 to 0.454; Supplemental eFig. 11). The results for adverse events were consistent across the inverse variance, Peto, and exact methods, indicating no material differences in the estimates (see eFig. 12).

### Quality-of-evidence assessment

Table 3 presents the certainty of the evidence as assessed using the CINeMA framework. For the primary outcome, pain, the confidence rating was moderate to low in the reaction phase and moderate to very low in both the regulation and recovery phases. Among the secondary outcomes, the CoE for crying time revealed low to very low confidence. For heart rate, confidence ranged from high to low in the reaction phase and low to very low in the regulation and recovery phases. Similarly, oxygen saturation showed low to very low CoE in the reaction phase and the respiratory rate in the recovery phase. The oxygen saturation in the regulation and recovery phases was rated as moderate to very low and high to moderate, respectively. Finally, the respiratory rate in the reaction phase and adverse events both showed a confidence range of moderate to very low and high to low, respectively. Among the three sweet solutions, most comparisons involving expressed breast milk are supported by evidence of very low certainty.

**Table 3:** Summary of mixed evidence’s confidence ratings (CRs)

Outcome	Comparison	k	WsB	Reporting bias	Indirectness	Imprecision	Heterogeneity	Incoherence	CR
Pain (reaction)									
	Control: EBM	3	No concerns	Low risk	No concerns	Some concerns	Some concerns	No concerns	Moderate
	Control: Glucose	8	No concerns	Low risk	No concerns	No concerns	Major concerns	No concerns	Low
	Control: Sucrose	7	No concerns	Low risk	No concerns	No concerns	Major concerns	No concerns	Low
	EBM: Glucose	2	No concerns	Low risk	No concerns	Some concerns	Some concerns	No concerns	Moderate
	EBM: Sucrose	2	No concerns	Low risk	No concerns	Some concerns	Some concerns	No concerns	Moderate
	Glucose: Sucrose	1	No concerns	Low risk	No concerns	Some concerns	Some concerns	No concerns	Moderate
Pain (regulation)									
	Control: EBM	4	Major concerns	Low risk	No concerns	No concerns	Some concerns	No concerns	Very low
	Control: Glucose	12	No concerns	Some concerns	No concerns	No concerns	Some concerns	No concerns	Moderate
	Control: Sucrose	4	No concerns	Low risk	No concerns	No concerns	Some concerns	No concerns	Moderate
	EBM: Glucose	5	Major concerns	Low risk	No concerns	Some concerns	Some concerns	No concerns	Very low
	Glucose: Sucrose	3	No concerns	Low risk	No concerns	Some concerns	Some concerns	No concerns	Moderate
	EBM: Sucrose	0	No concerns	Low risk	Some concerns	Major concerns	No concerns	No concerns	Low
Pain (recovery)									
	Control: EBM	6	Major concerns	Low risk	No concerns	No concerns	Some concerns	No concerns	Very low
	Control: Glucose	7	No concerns	Low risk	No concerns	No concerns	Some concerns	No concerns	Moderate
	Control: Sucrose	5	No concerns	Low risk	No concerns	No concerns	Some concerns	No concerns	Moderate
	EBM: Glucose	5	Major concerns	Low risk	No concerns	No concerns	Major concerns	No concerns	Very low
	EBM: Sucrose	1	No concerns	Some concerns	No concerns	No concerns	Major concerns	No concerns	Low
	Glucose: Sucrose	3	No concerns	Low risk	No concerns	No concerns	Major concerns	No concerns	Low
Crying time									
	Control: EBM	1	Major concerns	Some concerns	No concerns	Major concerns	No concerns	No concerns	Very low
	Control: Glucose	6	No concerns	Low risk	No concerns	No concerns	Major concerns	No concerns	Low
	Control: Sucrose	4	No concerns	Low risk	No concerns	No concerns	Major concerns	No concerns	Low
	EBM: Glucose	1	Major concerns	Some concerns	No concerns	Major concerns	No concerns	No concerns	Very low
	Glucose: Sucrose	1	No concerns	Low risk	No concerns	Major concerns	No concerns	No concerns	Low
	EBM: Sucrose	0	Major concerns	Low risk	Some concerns	Major concerns	No concerns	Major concerns	Very low
Heart rate (reaction)									
	Control: EBM	2	No concerns	Low risk	No concerns	No concerns	No concerns	No concerns	High
	Control: Glucose	3	No concerns	Low risk	No concerns	No concerns	No concerns	No concerns	High
	Control: Sucrose	8	No concerns	Low risk	No concerns	No concerns	No concerns	No concerns	High
	EBM: Sucrose	1	No concerns	Low risk	No concerns	No concerns	No concerns	No concerns	High
	Glucose: Sucrose	1	No concerns	Low risk	No concerns	No concerns	No concerns	No concerns	High
	EBM: Glucose	0	No concerns	Some concerns	Some concerns	No concerns	Some concerns	No concerns	Low
Heart rate (regulation)									
	Control: EBM	1	No concerns	Low risk	No concerns	Major concerns	No concerns	No concerns	Low
	Control: Glucose	1	No concerns	Low risk	No concerns	Major concerns	No concerns	No concerns	Low
	Control: Sucrose	3	Some concerns	Low risk	No concerns	Major concerns	No concerns	No concerns	Very low
	Glucose: Sucrose	1	No concerns	Low risk	No concerns	Major concerns	No concerns	No concerns	Low
	EBM: Glucose	0	No concerns	Some concerns	Some concerns	Major concerns	No concerns	No concerns	Very low
	EBM: Sucrose	0	No concerns	Low risk	Some concerns	Major concerns	No concerns	No concerns	Low
Heart rate (recovery)									
	Control: EBM	3	Some concerns	Low risk	No concerns	No concerns	Major concerns	No concerns	Very low
	Control: Glucose	4	No concerns	Low risk	No concerns	Major concerns	No concerns	No concerns	Low
	Control: Sucrose	4	Some concerns	Low risk	No concerns	Major concerns	No concerns	No concerns	Very low
	EBM: Glucose	1	Major concerns	Low risk	No concerns	Major concerns	No concerns	No concerns	Very low
	EBM: Sucrose	1	Some concerns	Low risk	Some concerns	Major concerns	No concerns	No concerns	Very low
	Glucose: Sucrose	1	No concerns	Low risk	No concerns	Major concerns	No concerns	No concerns	Low
Oxygen saturation (reaction)									
	Control: EBM	2	No concerns	Low risk	No concerns	No concerns	Major concerns	No concerns	Low
	Control: Glucose	4	No concerns	Low risk	No concerns	No concerns	Major concerns	No concerns	Low
	Control: Sucrose	6	Some concerns	Low risk	No concerns	No concerns	Major concerns	No concerns	Very low
	EBM: Sucrose	1	Some concerns	Low risk	No concerns	Some concerns	Some concerns	No concerns	Very low
	Glucose: Sucrose	1	No concerns	Low risk	No concerns	No concerns	Major concerns	No concerns	Low
	EBM: Glucose	0	No concerns	Low risk	Some concerns	Some concerns	Some concerns	Some concerns	Low
Oxygen saturation (regulation)									
	Control: EBM	1	No concerns	Low risk	No concerns	Some concerns	Some concerns	Major concerns	Very low
	Control: Glucose	2	No concerns	Low risk	No concerns	Some concerns	Some concerns	No concerns	Moderate
	Control: Sucrose	2	No concerns	Low risk	No concerns	Some concerns	Some concerns	No concerns	Moderate
	Glucose: Sucrose	1	No concerns	Low risk	No concerns	Some concerns	Some concerns	No concerns	Moderate
	EBM: Glucose	0	No concerns	Low risk	Some concerns	Major concerns	No concerns	Major concerns	Very low
	EBM: Sucrose	0	No concerns	Low risk	Some concerns	Some concerns	Some concerns	Major concerns	Very low
Oxygen saturation (recovery)									
	Control: EBM	3	No concerns	Low risk	No concerns	No concerns	Some concerns	No concerns	Moderate
	Control: Glucose	4	No concerns	Some concerns	No concerns	No concerns	No concerns	No concerns	Moderate
	Control: Sucrose	3	No concerns	Low risk	No concerns	No concerns	No concerns	No concerns	High
	EBM: Glucose	1	No concerns	Low risk	No concerns	Some concerns	No concerns	No concerns	Moderate
	EBM: Sucrose	1	No concerns	Low risk	No concerns	No concerns	No concerns	No concerns	High
	Glucose: Sucrose	1	No concerns	Low risk	No concerns	No concerns	Some concerns	No concerns	Moderate
Respiratory rate (reaction)									
	Control: Glucose	2	No concerns	Low risk	No concerns	Some concerns	Some concerns	No concerns	Moderate
	Control: Sucrose	3	No concerns	Low risk	No concerns	Major concerns	No concerns	No concerns	Low
	Glucose: Sucrose	1	No concerns	Low risk	No concerns	Major concerns	No concerns	No concerns	Low
Respiratory rate (recovery)									
	Control: EBM	1	Major concerns	Low risk	No concerns	Some concerns	Some concerns	No concerns	Very low
	Control: Glucose	3	Major concerns	Low risk	No concerns	No concerns	Major concerns	No concerns	Very low
	Control: Sucrose	2	No concerns	Low risk	No concerns	Major concerns	No concerns	No concerns	Low
	EBM: Glucose	1	Major concerns	Low risk	No concerns	Some concerns	Some concerns	No concerns	Very low
	Glucose: Sucrose	1	No concerns	Low risk	No concerns	Some concerns	Some concerns	No concerns	Low
	EBM: Sucrose	0	Major concerns	Some concerns	Some concerns	Some concerns	Some concerns	No concerns	Very low
Adverse events									
	Control: EBM	2	No concerns	Some concerns	No concerns	Some concerns	No concerns	No concerns	Low
	Control: Glucose	6	No concerns	Low risk	No concerns	No concerns	No concerns	No concerns	High
	Control: Sucrose	3	No concerns	Some concerns	No concerns	Some concerns	No concerns	No concerns	Low
	EBM: Glucose	2	No concerns	Low risk	No concerns	Some concerns	Some concerns	No concerns	Moderate
	Glucose: Sucrose	2	No concerns	Low risk	No concerns	Some concerns	No concerns	No concerns	Moderate
	EBM: Sucrose	0	No concerns	Some concerns	Some concerns	Some concerns	No concerns	No concerns	Low

*k *number of studies providing direct evidence, *WsB* within-study bias

## Discussion

To our knowledge, this is the first NMA to systematically examine the comparative effectiveness of various sweet solutions for optimal pain management in preterm infants. This review included 42 studies (2733 preterm infants). Thirty-eight of these studies were eligible for the network meta-analysis, which compared 3 distinct sweet solutions aimed at reducing pain responses during the reaction, regulation, and recovery stages. All the sweet solutions provided better effects of reducing pain than the control groups, with glucose showing the greatest reduction in pain. However, the secondary outcomes revealed differences in the efficacy of the interventions.

Our study confirmed the efficacy of sucrose and glucose in reducing procedural pain in preterm infants. In line with this study, a former PMA proved the analgesic effects of sucrose [29, 52–54, 75, 76] and glucose [7] in relieving procedural pain in neonates. Prior reviews have also indicated that expressed breast milk is an effective analgesic intervention [4, 70–72]. However, in our analysis, most evidence supporting the use of expressed breast milk was graded as having a very low certainty of evidence (CoE) (see Table 3). Consequently, while acknowledging its potential, we advise cautious interpretation regarding the efficacy of expressed breast milk based on the current evidence.

Previous studies [19, 35] and clinical guidelines [9, 55, 80] have supported the analgesic effect of sweet solutions for procedural pain in newborns, though often by examining them collectively as a single intervention. Our study builds upon this foundation by providing a critical refinement: we directly compare these solutions against one another using a network meta-analysis. This robust quantitative synthesis generates a ranking of treatment efficacy, thereby offering much-needed, specific evidence for preterm infants—a population frequently underrepresented in existing guidelines.

Even though our evidence showed there was no treatment to handle AEs required, glucose or sucrose administration in newborns needs careful attention. According to recent guidelines, a maximum of 30% glucose or sucrose solution is applicable for safety reasons [84]. Lower osmolarity (less than 24%) is more recommended for preterm infants to reduce the risk of adverse outcomes such as hyperglycaemia and necrotizing enterocolitis (NEC) [46, 50]. Furthermore, using high concentrations such as 50% glucose did not show the additional beneficial analgesic effect as shown in our sensitivity analysis (eTable 12).

The mechanism of analgesia induced by oral sweet solutions in humans is still not well understood [3, 31, 59]. It was postulated that endogenous opioids may be involved in the underlying mechanism [59]. Intraoral sucrose activates two key brainstem sites which are critically involved in modulating descending pain: neurons in the periaqueductal gray matter and in the nucleus raphe magnus [10]. This process should be mediated by the sweet sensation from the solutions [3]. Glucose is the only monosaccharide among the sweeteners examined [25, 26], which may make it easier to absorb [37]. Meanwhile, although both expressed breast milk and sucrose are in the disaccharide group [25], due to high concentrations of tryptophan as a precursor of melatonin [36], they may induce an increase in beta-endorphin concentrations [28].

A former Cochrane review and a PMA depicted no effect of sucrose use on heart rate, respiration rate, or oxygen saturation during or after painful procedures [52, 75, 76]. Another MA also showed no effect of expressed breast milk administration on heart range changes [72]. Due to an inadequate number of studies, the effects of glucose or other sweeteners on those given outcomes remain inconclusive [7, 19]. Our current review provides additional evidence of expressed breast milk’s superiority in reducing heart and respiratory rates in the recovery stage.

Several PMAs proposed glucose, sucrose, or expressed breast milk to reduce the total crying time of neonates undergoing painful procedures [7, 35, 53, 75, 76]. This current review supported the efficacies of glucose and sucrose in reducing crying times. However, our NMA estimate did not prove the efficacy of expressed breast milk. Although current and former meta-analyses did not depict signs of inconsistency, the contrasting results may have been induced by the different populations. This NMA only focused on preterm infants, while a preceding PMA also included healthy term infants [72].

We found a review that reported similar AEs between sweet solutions and topical anesthesia [19]. However, this NMA had insufficient studies to produce estimates to systematically compare AEs among treatments. Our results provide additional evidence which indicates potential AEs for preterm infants consuming glucose as oral analgesia. This finding was congruent with a previous report which showed higher AEs in infants receiving glucose, compared to those who received water [7]. Nevertheless, AEs self-recovered with no requirement for professional interventions [6, 49]. AEs more likely occur in preterm infants on which our study focused [52, 76]. Meanwhile, previous meta-analyses incorporated healthy newborns in their studies [53, 76]. Moreover, using the inverse variance to estimate the effect of rare events in an NMA may potentially cause biases [27].

For term neonates, a recommended single dose of 24% sucrose ranges from 0.1 to 1 mL (0.2–0.5 mL/kg), given no more than 2 min prior to the painful procedure. In preterm infants, more cautious dosing is recommended due to their immature gastrointestinal and metabolic functions. Therefore, a single administration of 24% sucrose in this population should typically not exceed 0.05–0.5 mL (0.1 mL/kg), accompanied by careful monitoring for potential adverse effects [76]. If repeated administrations are indicated, a maximum of 10 doses per 24 h should be avoided, particularly for preterm infants [44]. During the sweet solution administration, healthcare professionals should note any AEs such as heart rate change, oxygen desaturation, apnea, choking, vomiting, the occurrence of residues in the stomach, and abdominal distention and be ready to provide appropriate rescue measures as needed.

### Limitations, strength, and future directions

This study has several limitations. First, the substantial heterogeneity observed across outcomes may partly reflect unmeasured differences in infants’ baseline clinical status. Preterm infants likely varied in illness severity, yet most trials did not report sufficient details to allow stratified analyses or adjustment, which may have contributed to variability in effect estimates. Second, intervention delivery differed markedly between studies, including solution concentration, dose, timing, frequency, and administration method, creating practical heterogeneity that limits firm conclusions about the optimal regimen. Third, pain was assessed using multiple instruments. Although we used SMDs to enable pooling, differences in scale properties and timing of assessment may still have introduced measurement-related heterogeneity and could influence relative rankings.

Some evidence produced in this study relied on low or very low certainty, particularly for glucose versus expressed breast milk, and many comparisons for heart rate in the recovery phase were supported by very low certainty evidence (Table 3). Notably, direct head-to-head evidence comparing expressed breast milk and sucrose was unavailable for pain reactivity and adverse events, increasing reliance on indirect comparisons. Taken together, variability in intervention delivery, outcome measurement, and participant characteristics—combined with uncertainty in evidence quality—means that the findings should be interpreted cautiously in clinical settings. In addition, we could not evaluate the effects of repeated administrations or cumulative exposure because dosing schedules and repeated-use data were limited and inconsistently reported.

Although comparison-adjusted funnel plots and Egger’s tests did not suggest small-study effects, publication bias remains possible, as unpublished studies or selectively reported outcomes may not have been captured despite searching gray literature and dissertations. Missing or incompletely reported outcome data were also common; several trials lacked measures of dispersion or presented outcomes in formats requiring conversion, and adverse events were often variably defined and underreported. While we applied prespecified methods to derive missing statistics, these imputations may add uncertainty and could affect effect estimates and treatment rankings, especially for outcomes informed by relatively few studies. Finally, generalizability is constrained by clinical and methodological diversity: gestational age and clinical stability varied across trials, painful procedures differed, and sweet-solution regimens and co-interventions were not standardized. Therefore, results may not fully generalize to extremely preterm or critically ill infants, routine repeated dosing, or settings with different monitoring and supportive-care practices.

Our study also has some strengths. The main conclusions were robust to sensitivity analyses, with findings remaining consistent after excluding trials at high risk of bias and those using extremely high doses. For the pain outcomes, the mean path length was below the prespecified threshold, indicating that most estimates were supported by relatively strong direct evidence across the network and thereby strengthening the credibility of the comparisons. In addition, we synthesized a comparatively large body of randomized evidence with substantial sample size, applying clearly defined eligibility criteria and rigorous, transparent methods for study selection, data handling, and network meta-analysis.

Future research must prioritize four key areas to advance the safe and effective use of sweet solutions for analgesia in preterm infants. First, comprehensive reporting of AEs’ data is essential to fully establish the safety profile of these interventions in this vulnerable population. Trialists should report AEs as individual events (e.g., desaturation, bradycardia, regurgitation, or vomiting) rather than using composite outcomes. Second, well-designed randomized controlled trials are needed to confirm efficacy, both as monotherapies and in combination with other non-pharmacological measures, with a specific focus on extremely preterm infants. Third, investigations into optimal dosing regimens, including the effects of repeated applications and different concentrations, are critical to guide clinical practice. Lastly, elucidating the underlying analgesic mechanisms of sweet solutions could provide valuable insights into why glucose appears to be the most effective option.

## Conclusions

Based on low-to-moderate certainty evidence, sweet solutions were effective in reducing procedural pain in preterm infants. Among them, glucose appeared to be the most effective intervention, demonstrating a greater likelihood of a superior analgesic effect across all procedural phases, albeit with moderate to low CoE. The administration of sweet solutions showed no significant association with physiological stabilization (i.e., heart rate, respiratory rate, and oxygen saturation), with the exception of expressed breast milk. However, these specific outcome estimates are derived from a limited number of studies and are supported by evidence of low to very low certainty. Consequently, these particular findings require cautious interpretation.

Given the potential for adverse effects, glucose administration should be carefully monitored and implemented by experienced healthcare professionals. In cases where glucose is not appropriate, expressed breast milk or sucrose may be considered as alternative options. Among the various types of sweet solutions, the evidence from expressed breast milk is graded as having low certainty. Therefore, any comparisons involving expressed breast milk should be interpreted with caution.

## Supplementary Information

Additional file 1: eTable 1. Search Strategy. eTable 2. Review eligibility criteria. eTable 3. Validated pain assessment tools. eTable 4. Characteristics population in trials include studies. eTable 5. List of excluded studies. eTable 6. Characteristics of direct comparison in the network meta-analysis. eTable 7. Treatment ranking probability using P-Score. eTable 8. Assessment of heterogeneity. eTable 9. Split of direct and indirect evidence. eTable 10. Assessment of global inconsistency. eTable 11. Full design-by-treatment interaction random effects model. eTable 12. Sensitivity analysis. eTable 13. Meta-regression. eFigure 1. The mean path length. eFigure 2. Risk of bias. eFigure 3. Network geometry. eFigure 4. Forest plot of direct comparison. eFigure 5. Forward plot of Cook’s distance for model of pain during recovery phase. eFigure 6. Forward plot of ratio of variance for model of pain during recovery phase. eFigure 7. Forward plot of z-values that compare relative treatment effects estimated from direct and indirect evidence. eFigure 8. Forward plot for P-score on pain during recovery phase. eFigure 9. Adjusted publication bias. eFigure 10. Forest plots of differences in pain during recovery phase by risk of bias. eFigure 11. Forest plots of differences in pain during recovery phase by painful procedure. eFigure 12. Forest plot of adverse events in pain reaction using the Peto, inverse variance, and exact methods.

## Data Availability

The datasets are available from the corresponding author on reasonable request.

## Abbreviations

AEs: Adverse events
CINeMA: Confidence in Network Meta-Analysis
CoE: Certainty of the evidence
CI: Confidence interval
DAN: Douleur Aiguë Nouveau-né (Newborn Acute Pain) Scale
GRADE: Grading of Recommendations Assessment, Development and Evaluation
MDs: Mean differences
NFCS: Neonatal Facial Coding Scale
NIPS: Neonate/Infant Pain Scale
NMA: Network meta-analysis
PIPP: Premature Infant Pain Profile
PIPP-R: Premature Infant Pain Profile-Revised
PMAs: Pairwise meta-analyses
PRISMA: Preferred Reporting Items for Systematic reviews and Meta-Analysis
RCTs: Randomized clinical trials
RoB: Risk of bias
RRs: Risk ratios
SMD: Standardized mean difference
SUCRA: Surface under the cumulative ranking curve
