# Correction: The comparative efficacy and safety of sweet solutions to reduce preterm infants’ pain levels: a systematic review and network meta‑analysis

**DOI:** 10.1186/s13643-026-03324-5

**Published:** 2026-10-09

**Authors:** Defi Efendi, Yi‑No Kang, Regina Natalia, Ariesta Milanti, Putri M. T. Marsubrin, Christina Yeni Kustanti, Kee‑Hsin Chen

**Affiliations:** 1 School of Nursing, Taipei Medical University, Taipei, Taiwan; 2 Department of Pediatric Nursing, Faculty of Nursing, Universitas Indonesia, Depok, West Java Indonesia; 3 Neonatology Unit, Universitas Indonesia Hospital, Depok, West Java Indonesia; 4 Evidence‑Based Medicine Center & Research Center of Big Data and Meta‑Analysis, Wan Fang Hospital, Taipei Medical University, Taipei, Taiwan; 5 Cochrane Taiwan, Taipei Medical University, Taipei, Taiwan; 6 Institute of Health Policy and Management, College of Public Health, National Taiwan University, Taipei, Taiwan; 7 School of Nursing, Institut Kesehatan Mitra Bunda, Batam, Indonesia; 8 Strategic Delivery Unit, Ministry of Health, Jakarta, Indonesia; 9 Neonatology Division, Department of Child Health, Cipto Mangunkusumo National General Hospital, Faculty of Medicine Universitas Indonesia, Jakarta, Indonesia; 10 Study Program of Nursing Science, Sekolah Tinggi Ilmu Kesehatan Bethesda Yakkum, Yogyakarta, Indonesia; 11 Lotus Care, Private Clinic for Wound & Palliative Care, Homecare, Yogyakarta, Indonesia; 12 Post‑Baccalaureate Program in Nursing, College of Nursing, Taipei Medical University, Taipei, Taiwan; 13 Department of Nursing & Research Center in Nursing Clinical Practice, Wan Fang Hospital, Taipei Medical University, Taipei, Taiwan

**Correction: Syst Rev 15, 50 (2026)**

**https://doi.org/10.1186/s13643-025–03043-3**

Following the official publication of the original article [1], the authors noticed some minor, typographical errors in the initials of the independent researchers. The corrections are given below in bold:

1. “Two researchers (O.O. and O.O)” should be “Two researchers (**A.M. and R.N.**)
2. “a consensus moderated by the first author (O.O.)” should be “a consensus moderated by the first author (**D.E.**)”
3. “O. O. and O. O. independently extracted data from the reviewed studies” should be “**D. E. and R. N** independently extracted data from the reviewed studies.”
4. “Two independent researchers (O. O. and O. O.) evaluated the risk of bias (RoB)” should be “Two independent researchers (**A.** **M.** and **R.** **N.**) evaluated the risk of bias (RoB)”

Furthermore, minor errors were also spotted in capturing the Standard Mean Difference. Under the heading Secondary Outcomes, “SMD” should be captured as “**MD**” only.

The corrections do not compromise the validity of the conclusions and the overall content of the article. The original article [1] has been corrected.

## References

[1] Efendi D, Kang YN, Natalia R, et al. The comparative efficacy and safety of sweet solutions to reduce preterm infants’ pain levels: a systematic review and network meta-analysis. Syst Rev. 2026;15:50. 10.1186/s13643-025-03043-3. PMC12882208 41514374

