# Supplementary material for: The comparative efficacy and safety of sweet solutions to reduce preterm infants’ pain levels: a systematic review and network meta-analysis
Source: Syst Rev. 2026 Jan 10;15:50. doi: 10.1186/s13643-025-03043-3 (PMC12882208; doi:10.1186/s13643-025-03043-3)
Supplement: Supplementary file 1 — Additional file 1: eTable 1. Search Strategy. eTable 2. Review eligibility criteria. eTable 3. Validated pain assessment tools. eTable 4. Characteristics population in trials include studies. eTable 5. List of excluded studies. eTable 6. Characteristics of direct comparison in the network meta-analysis. eTable 7. Treatment ranking probability using P-Score. eTable 8. Assessment of heterogeneity. eTable 9. Split of direct and indirect evidence. eTable 10. Assessment of global inconsistency. eTable 11. Full design-by-treatment interaction random effects model. eTable 12. Sensitivity analysis. eTable 13. Meta-regression. eFigure 1. The mean path length. eFigure 2. Risk of bias. eFigure 3. Network geometry. eFigure 4. Forest plot of direct comparison. eFigure 5. Forward plot of Cook’s distance for model of pain during recovery phase. eFigure 6. Forward plot of ratio of variance for model of pain during recovery phase. eFigure 7. Forward plot of z-values that compare relative treatment effects estimated from direct and indirect evidence. eFigure 8. Forward plot for P-score on pain during recovery phase. eFigure 9. Adjusted publication bias. eFigure 10. Forest plots of differences in pain during recovery phase by risk of bias. eFigure 11. Forest plots of differences in pain during recovery phase by painful procedure. eFigure 12. Forest plot of adverse events in pain reaction using the Peto, inverse variance, and exact methods. [file 13643_2025_3043_MOESM1_ESM.docx]

**Supplemental Online Content**

**eTable 1.** Search Strategy

**eTable 2.** Review eligibility criteria

**eTable 3.** Validated pain assessment tools

**eTable 4.** Characteristics population in trials include studies

**eTable 5.** List of excluded studies

**eTable 6**. Characteristics of direct comparison in the network meta-analysis

**eTable 7**. Treatment ranking probability using P-Score

**eTable 8**. Assessment of heterogeneity

**eTable 9**. Split of direct and indirect evidence

**eTable 10.** Assessment of global inconsistency

**eTable 11.** Full design-by-treatment interaction random effects model

**eTable 12.** Sensitivity analysis

**eTable 13.** Meta-regression

**eFigure 1.** The mean path length

**eFigure 2.** Risk of bias

**eFigure 3.** Network geometry

**eFigure 4.** Forest plot of direct comparison

**eFigure 5.** Forward plot of Cook’s distance for model of pain during recovery phase

**eFigure 6.** Forward plot of ratio of variance for model of pain during recovery phase

**eFigure 7.** Forward plot of z-values that compare relative treatment effects estimated from direct and indirect evidence

**eFigure 8.** Forward plot for P-score on pain during recovery phase

**eFigure 9.** Adjusted publication bias

**eFigure 10.** Forest plots of differences in pain during recovery phase by risk of bias

**eFigure 11.** Forest plots of differences in pain during recovery phase by painful procedure

**eFigure 12.** Forest plot of adverse events in pain reaction using the peto, inverse variance, and exact methods

**eTable 1. Search Strategy**

Sample of search strategy

|  | **EMBASE** |  |
| --- | --- | --- |
| #21 | #6 AND #13 AND #16 AND ([article]/lim OR [article in press]/lim OR [review]/lim OR [preprint]/lim) AND ([chinese]/lim OR [english]/lim OR [indonesian]/lim) AND ([newborn]/lim OR [infant]/lim OR [child]/lim) | 2012 |
| #20 | #6 AND #13 AND #16 AND ([article]/lim OR [article in press]/lim OR [review]/lim OR [preprint]/lim) AND ([chinese]/lim OR [english]/lim OR [indonesian]/lim) | 2257 |
| #19 | #6 AND #13 AND #16 AND [humans]/lim AND [clinical study]/lim | 1844 |
| #18 | #6 AND #13 AND #16 AND ([cochrane review]/lim OR [systematic review]/lim OR [meta analysis]/lim OR [randomized controlled trial]/lim OR 'controlled clinical trial'/de) AND ([article]/lim OR [article in press]/lim OR [review]/lim OR [preprint]/lim) AND ([chinese]/lim OR [english]/lim OR [indonesian]/lim) AND ([newborn]/lim OR [infant]/lim) AND [humans]/lim AND [clinical study]/lim | 982 |
| #17 | #6 AND #13 AND #16 | 2897 |
| #16 | #14 OR #15 | 2174255 |
| #15 | randomized:ti,ab,kw OR placebo:ti,ab,kw OR randomly:ti,ab,kw OR trial:ti,ab,kw | 2060046 |
| #14 | 'randomized controlled trial'/exp | 745948 |
| #13 | #11 OR #12 | 840912 |
| #12 | 'breast milk taste':ti,ab,kw OR 'breast milk':ti,ab,kw OR 'human milk':ti,ab,kw OR 'expressed breast milk':ti,ab,kw OR 'expressed human milk':ti,ab,kw OR 'donor human milk':ti,ab,kw OR 'mother milk':ti,ab,kw OR 'mother own milk':ti,ab,kw | 31921 |
| #11 | #7 OR #8 OR #9 OR #10 | 809759 |
| #10 | glucose:ti,ab,kw OR 'oral glucose':ti,ab,kw OR 'orally administered glucose':ti,ab,kw OR 'glucose solution':ti,ab,kw | 723158 |
| #9 | dextrose:ti,ab,kw OR 'oral dextrose':ti,ab,kw OR 'orally administered dextrose':ti,ab,kw OR 'dextrose solution':ti,ab,kw | 17470 |
| #8 | sucrose:ti,ab,kw OR 'oral sucrose':ti,ab,kw OR 'orally administered sucrose':ti,ab,kw OR 'sucrose solution':ti,ab,kw | 81255 |
| #7 | sweetness:ab,ti OR 'sweet taste':ab,ti OR 'sweet-tasting solution':ab,ti OR 'sweet tasting solution':ab,ti OR 'oral sugar':ab,ti OR 'oral sweet solution':ti,ab,kw OR 'oral solution':ti,ab,kw | 7583 |
| #6 | #4 OR #5 | 760384 |
| #5 | preemie:ab,ti OR 'very premature infant':ab,ti OR 'very preterm infant':ab,ti OR 'very low birth weight':ab,ti OR 'extremely low birth weight':ab,ti | 13638 |
| #4 | 'newborn'/exp OR 'prematurity'/exp OR 'low birth weight'/exp OR 'small for date infant'/exp | 759937 |
| #3 | 'newborn'/exp OR 'prematurity'/exp OR 'low birth weight'/exp | 759937 |
| #2 | 'newborn'/exp OR 'prematurity'/exp | 728527 |
| #1 | 'newborn'/exp | 659387 |

**MEDLINE**

| (("sweet taste"[Title/Abstract] OR "sweet solution"[Title/Abstract] OR "sweet tasting solution"[Title/Abstract] OR ("glucose"[Title/Abstract] OR "oral glucose"[Title/Abstract] OR "oral sugar"[Title/Abstract] OR "oral solution"[Title/Abstract] OR "orally administered glucose"[Title/Abstract] OR "glucose solution"[Title/Abstract]) OR ("sucrose"[Title/Abstract] OR "oral sucrose"[Title/Abstract] OR "orally administered sucrose"[Title/Abstract] OR "sucrose solution"[Title/Abstract]) OR ("dextrose"[Title/Abstract] OR "oral dextrose"[Title/Abstract] OR "dextrose solution"[Title/Abstract]) OR ("breast milk"[Title/Abstract] OR "breast milk taste"[Title/Abstract] OR "humanmilk"[Title/Abstract] OR "expressed breast milk"[Title/Abstract] OR "expressed human milk"[Title/Abstract] OR "mother's milk"[Title/Abstract] OR "mother's own milk"[Title/Abstract] OR "donor human milk"[Title/Abstract] OR "donor milk"[Title/Abstract])) AND ("infant, newborn"[MeSH Terms] OR ("infant"[All Fields] AND "newborn"[All Fields]) OR "newborn infant"[All Fields] OR "neonatal"[All Fields] OR "neonate"[All Fields] OR "neonates"[All Fields] OR "neonatality"[All Fields] OR "neonatals"[All Fields] OR "neonate s"[All Fields] OR ("infant, premature"[MeSH Terms] OR ("infant"[All Fields] AND "premature"[All Fields]) OR "premature infant"[All Fields] OR ("preterm"[All Fields] AND "infant"[All Fields]) OR "preterm infant"[All Fields]) OR ("low-birth weight"[Title/Abstract] OR "low-birth weight"[Title/Abstract] OR "low-birth weights"[Title/Abstract] OR "low-birth weights"[Title/Abstract] OR "low birthweight"[Title/Abstract] OR "low birthweights"[Title/Abstract] OR "lbw"[Title/Abstract] OR "vlbw"[Title/Abstract] OR "elbw"[Title/Abstract])) AND ("randomized controlled trial"[Publication Type] OR "randomized controlled trials as topic"[MeSH Terms] OR "randomized controlled trial"[All Fields] OR "randomised controlled trial"[All Fields])) AND ((fft[Filter]) AND (humans[Filter]) AND (chinese[Filter] OR english[Filter] OR indonesian[Filter]) AND (newborn[Filter] OR allinfant[Filter])) | Full text, Chinese, English, Indonesian, Newborn: birth-1 month, Infant: birth-23 months, Humans | 1,839 |
| --- | --- | --- |
|  |  |  |

**ProQuest**

| ((newborn* OR "preterm infant" OR "preterm newborn*" OR prematur* OR "low birth weight*" OR baby OR babies OR neonat* OR infant OR infants OR "pre mature" OR "pre term" OR preemie OR "small gestational age" OR "term neonat*" OR "term newborn*" OR "newborn infant" OR "full-term neonat*" OR "very preterm infant" OR "extremely preterm infant" OR "very premature infant" OR "extremely premature" OR "very low birth weight*" OR "extremely low birth weight*" OR elbw OR vlbw OR lbw OR sga)) AND (abstract("sweet taste" OR "sweet solution*" OR "sweet tasting solution*" OR "oral sugar" OR "oral sweet solution*" OR "oral solution*" OR sucrose OR "oral sucrose" OR "orally administered sucrose" OR "sucrose solution*") OR abstract(glucose OR "oral glucose" OR "orally administered glucose" OR "glucose solution*") OR abstract(dextrose OR "oral dextrose" OR "orally administered dextrose" OR "dextrose solution*") OR abstract("breast milk taste" OR "breast milk" OR "human milk" OR "expressed breast milk" OR "expressed human milk" OR "mother's milk" OR "mother's own milk" OR "donor human milk")) AND abstract("controlled trial, randomized" OR "randomised controlled study" OR "randomised controlled trial" OR "randomized controlled study" OR "trial, randomized controlled" OR "randomized clinical trial*" OR "randomized clinical trial*" OR randomized OR randomised OR placebo OR randomly OR trial*) | ProQuest Dissertations & Theses Global | 883 |
| --- | --- | --- |

**eTable 2.** **Review eligibility criteria**

| **Patient** | Preterm infants |
| --- | --- |
| **Intervention** | Oral sweet solution: glucose, sucrose, and breast milk |
| **Comparator** | Sterile water (placebo), no treatment/intervention, and routine care |
| **Outcomes** | Primary Outcome: Pain |
| **Study design** | Randomized clinical trial |
| **Inclusion criteria** | 1. Randomized controlled trial. 2. Preterm infants with gestational age under 37 weeks. 3. Indication for pain procedure |
| **Exclusion criteria** | 1. Infants who were critically ill with altered consciousness 2. Combine with pharmaceutical therapy 3. Observational study designs, quasi experiment, or single arm study 4. Review articles, observational studies, letter to editor, and abstract |

**eTable 3. Validated pain assessment tools**

| **No** | **Instruments** | **Score** | **Interpretation** | **Notes** |
| --- | --- | --- | --- | --- |
| 1 | Premature Infant Pain Profile (PIPP)^1^ | - Full term: 0 – 18 - < 28 gestational age: 0 - 21 | Physiology and behavior items were given a numerical score of 0, 1, 2, 3 and contextual items gestational age (GA) and behavioural state (BS) were given a numerical score of 3, 2, 1, 0.   - Score < 6 indicates no to minimal pain. - Score > 12 indicates moderate to severe pain. | PIPP was a multidimensional pain score consist of 3 behavioral items, 2 physiological items, and 2 contextual items. |
| 2 | Douleur Aigue¨ Nouveaune´  (DAN) Scale^2^ | Pain scale ranging from 0 – 10. | The higher total scale, the more severe pain sensation. | Scales were developed on a behavioral basis to assess acute pain in term and preterm neonates. The three items evaluated were: facial expressions, limb movements, and vocal expressions (sounds). |
| 3 | Neonatal Infant Pain Scale (NIPS)^3^ | Minimum and maximum sore was 0 to 7 | A higher total score indicates more pain | Six categories of behavior were identified, namely, facial expressions, crying, breathing patterns, arm movements, leg movements, and responses to stimuli (states of arousal). NIPS is used in premature, full-term, and infants up to 1 year of age. |
| 4 | Neonatal Facial Coding System (NFCS)^4^ | - Full term: 0 – 9 - Preterm: 0 - 10 | Point of 0 is given for the facial action that did not occur and 1 for the facial action that occurred. | Assess facial expressions to assess pain. Interpret the baby's facial activity reported as taking eight hours for one person. |
| 5 | Neonatal Pain, Agitation and Sedation Scale (N-PASS)^5^ | 0 to 10 | Five criteria were scored 0.1 or 2 for pain/agitation and 0.1 or 2 for sedation. A high pain/agitation score indicates more pain, and a low sedation score indicates a decreased response to stimulation, or a deeper level of sedation. | Five indicators are included in the N-PASS, selected for their validity, clinical applicability, and ease of assessment. Crying/irritability, behavior/state, facial expressions, extremities/tone, and vital signs (heart rate, respiratory rate, blood pressure, and/or oxygen saturation). |

**eTable 4.** **Characteristics population in trials include studies.**

| **No** | **1^st^ Author (Year)** | **Country** | **Design** | **GA / PMA^a^**  (week, mean(SD)) | **Male  n (%)** | **BW/ CW^b^**  (gr, mean(SD)) | **Total sample** | **Pain procedure** | **Groups** | | **Outcomes** |
| --- | --- | --- | --- | --- | --- | --- | --- | --- | --- | --- | --- |
|  |  |  |  |  |  |  |  |  | **Interventions** | **Control** |  |
| 1 | Abad* (1996)^6^ | Spain | Parallel | **Placebo:**  33.3 (2.5) **Sucrose 12%:** 35.6 (1.3) **Sucrose 24%:** 33.7 (2.5) | - | **Placebo:**  1770 (438) **Sucrose 12%:** 2441 (835) **Sucrose 24%:** 1835 (682) | 28 | Venepunctures | Sucrose 12%  Sucrose 24% | Placebo | - Crying time - Heart rate - Respiratory rate - Oxygen saturation |
| 2 | Acharya* (2004)^7^ | UK | Cross-over | 30.5 (2.3) | 22 (56.4%) | - | 39 | Venepunctures | Sucrose 25% | Placebo | - Heart rate - Oxygen saturation |
| 3 | Axelin* (2009)^8^ | Finland | Cross-over | 28 (2.3) | - | 1123 (327) | 20 | Heel-stick, pharyngeal suctioning | Glucose 25% | Placebo | Pain |
| 4 | Bellieni* (2003)^9^ | Italy | Cross-over | Median (range): 31 (30-34) | 7 (41.2%) | - | 17 | Heel-stick | Glucose 10% | No treatment | Crying time |
| 5 | Bellieni* (2001)^10^ | Italy | Cross-over | - | 7 (41.2%) | - | 17 | Heel-stick | Glucose 10% Placebo | No treatment | Pain |
| 6 | Boyer* (2004)^11^ | Canada | Parallel | **Sucrose 24% ^a^:** 28 (1.6)  **Placebo:**  27.7 (1.9) | - | - | 41 | Heel-stick, venepuncture, suctioning | Sucrose 24% | Placebo | - Heart rate - Salivary cortisol |
| 7 | Boyle (2006)^12^ | Canada | Parallel | Median (range): **Sucrose 33%:** 29 (25-34) **Placebo:**  27 (24-30) | - | Median (range):  **Sucrose 33%:**  1030 (810-1570) **Placebo:**  1000 (755-1550) | 20 | RoP screening | Sucrose 33% | Placebo | Pain |
| 8 | Bucher (1995)^13^ | Switzerland | Parallel | - | - | - | 16 | Heel-stick | Sucrose 50% | Placebo | - Heart rate - Oxygen saturation - PO_2_ and PCO_2_ |
| 9 | Bueno* (2012)^14^ | Brazil | Parallel | **EBM:**  35.5 (0.7) **Glucose 25%:**  35.8 (0.7) | EBM:  30 (53.6%) **Glucose 25%:** 35 (61.4%) | **EBM:**  2460.5 (482.2) **Glucose 25%:** 2235.7 (456.8)  **EBM:^b^**  2395.5 (482.2) **Glucose 25%:** 2184.7 (444.3) | 113 | Heel-stick | EBM | Glucose 25% | Pain |
| 10 | Carbajal* (2002)^15^ | France | Cross-over | Mean  (95% CI):  28.1  (27.3–29.0) | 17 (70,8%) | Mean (95% CI): 1036  (944–1128)  Mean (95% CI):^b^ 1234  (1120–1348) | 24 | Subcutaneous injections | Glucose 30% | Placebo | Pain |
| 11 | Cirik*  (2020)^16^ | Turkey | Parallel | **EBM:**  33.35 (0.80) **No treatment:**  33 (0.87) | - | **EBM:^b^**  2081.42 (396.94)  **No treatment:**  2016.27 (364.20) | 64 | OGT insertion | EBM | No treatment | - Pain - Heart rate - Oxygen saturation |
| 12 | Da Costa* (2013)^17^ | Brazil | Parallel | **Glucose 25%:**  30.5 (1.9) **No treatment:**  29.9 (2.0) | - | **Glucose 25%:**  1287.80 (255.1)  **No treatment**:  1.227 (293.6)  **Glucose 25%:^b^**  1904.4 (330.2)  **No treatment:**  1801 (363.8) | 124 | RoP screening | Glucose 25% | No treatment | Pain |
| 13 | Dehghani* (2019)^18^ | Iran | Parallel | **No treatment:**  35.45 (0.66) **Glucose 50%:** 35.51 (0.62) | 54 (81.8%) | **No treatment:^b^**  1876.97 (347) **Glucose 50%:** 2028.48 (423.874) | 66 | Phlebotomy | Glucose 50% | No treatment | Pain |
| 14 | Desai*  (2017)^19^ | India | Parallel | **EBM:**  32.76 (3.96) **Sucrose:**  32.91 (3.45) | - | **EBM:^b^**  1358.52 (573.93) **Sucrose:**  1430.50 (648.89) | 72 | Suctioning | EBM | Sucrose (NA) | Pain |
| 15 | Deshmukh* (2002)^20^ | India | Parallel | **Dextrose 10%:**  34.05 (1.51) **Dextrose 25%:**  34.2 (1.36)  **Placebo:**  34.55 (1.53) | Sex ratio (M:F)  **Dexrose 10%** (n=20): 0.7:1 **Dextrose 25%** (n=20): 0.7:1  **Placebo** (n=20): 1.5:1 | **Dextrose 10%:** 1172.5 (274.73) **Dextrose 25%:** 1706.5 (253.42)  **Placebo:**  1532.5 (179.33) | 60 | Venepuncture | Dextrose 10%  Dextrose 25% | Placebo | - Crying time - Heart rate - Respiratory rate - Oxygen saturation |
| 16 | Elserafy* (2009)^21^ | Saudi Arabia | Parallel | 32.4 (2.9)^a^ | - | 1700 (600) | 36 | Heel-stick and/or venepuncture | Placebo  Sucrose 24% | No treatment | - Crying time - Heart rate - Respiratory rate - Oxygen saturation - Blood pressure |
| 17 | Freire* (2008)^22^ | Brazil | Parallel | **Glucose 25%:**^a^ 233.7 days (9.15)  **No treatment:** 236.09 days (10.97) |  | **Glucose 25%:^b^** 1559.03 (329.55)  **No treatment:** 1469.39 (306.11) | 64 | Heel-stick | Glucose 25% | No treatment | - Crying time - Heart rate - Oxygen saturation - Behavioral state indicators |
| 18 | Gaspardo* (2008)^23^ | Brazil | Parallel | Median (range): **Sucrose 25%:** 30 (25-33) **Placebo:**  31 (27-33) | **Sucrose 25%:** 6 (35%)  **Placebo:**  10 (62%) | Median (range):  **Sucrose 25%:** 995 (640-1430) **Placebo:**  1177 (685-1360) | 33 | Needle-related procedures, endotracheal tube introduction and suctioning, gavage insertion for feeding, and removal of electrode leads and tape. | Sucrose 25% | Placebo | - Pain - Crying time - Heart rate - Infant sleep-wake states |
| 19 | Hsieh*  (2017)^24^ | Taiwan | Crosss-over | Median (range): 32.3 (26.6 – 35.9) | 7 (35%) | Median (range):  1596 (766 - 2435) | 20 | Heel-stick | Dextrose 10% EBM Placebo | No treatment | Pain |
| 20 | Johnston (1997)^25^ | Canada | Parallel | **Sucrose 24%:** 31.4 (2.89) **Placebo:**  31.4 (2.9) | - | Sucrose 24%: 1726 (545) Water:  1583 (447) | 47 | Heel-stick | Sucrose 24% | Placebo | - Pain - Heart rate |
| 21 | Johnston* (1999)^26^ | Canada | Parallel | **Single Sucrose 24%:**  30 (2.3) **Repeated Succrose 24%:**  30.7 (2.3) **Placebo:**  30.9 (2.1) | - | **Single Sucrose 24%:^b^**  1533 (374) **Repeated Sucrose 24%:** 1400 (469) **Placebo:**  1513 (432) | 48 | Heel-stick | Single dose sucrose 24%   Repeated dose sucrose 24% | Placebo | Pain |
| 22 | Kazmi* (2020)^27^ | Pakistan | Parallel | - | **Dextrose 25%:**  Male: 103 (51.5%) **EBM:** Male: 102 (51%) | **Dextrose 25%:^b^**  2900 (0.490)  **EBM:**  2950 (0.480) | 400 | Venepuncture | Dextrose 25% | EBM | Pain |
| 23 | Kristoffersen* (2011)^28^ | Sweden | Crosss-over | Median (range)   **No treatment:** 32.3 (29.9-34.6) **Placebo:**  32.1 (29.4-34.9) **Sucrose 30%:** 32 (29.3-35.1) | Male: 7 Female: 17 | * Median (range): **No treatment:**  1580 (750-2470) **Placebo:** 1460 (970-2160) **Sucrose 30%:**  1540 (990-2250) | 24 | Insertion of a feeding tube | Sucrose 30%  Placebo | No treatment | Pain |
| 24 | Kumari* (2016)^29^ | India | Parallel | **Glucose 25%:** 35.53 (0.73) **Sucrose 24%:** 35.56 (0.78) | **Glucose 25%:**  26 (55.32%) **Sucrose 24%:**  29 (61.7%) | - | 94 | Heel-stick | Glucose 25% | Sucrose 24% | Pain |
| 25 | McCullough* (2007)^30^ | UK | Parallel | - | 11 (21.6%) | **Sucrose 24%:^b^**  1700 kg (380) **Placebo:**  1680(350) | 51 | NGT insertion | Sucrose 24% | Placebo | - Pain - Crying time - Heart rate - Oxygen saturation |
| 26 | Naik* (2021)^31^ | India | Parallel | **No treatment:**  33.27 (1.79) **EBM:**  33.35 (2.12) | **No treatment:**  17 (26.6%) **EBM:**  17 (26.6%) | **No treatment:**   1440 (260) **EBM:**  1470 (380) | 64 | RoP examination | EBM | No treatment | Pain |
| 27 | Okan*  (2007)^32^ | Turkey | Cross-over | 30.5 (2.7)  32.3 (1.5)^a^ | 15 (48.4%) | 1401 (406)    1524 (352)^b^ | 90 | Heel-stick | Sucrose 20% Glucose 20% | Placebo | - Pain - Crying time - Heart rate - Oxygen saturation - Respiratory rate |
| 28 | Olsson* (2010)^33^ | Sweeden | Parallel | **Glucose 30%:** 28.9 (2.1) **Placebo:**  27.9 (2.5) | **Glucose 30%:** 8 (57%) **Placebo:**  7 (43%) | **Glucose 30%:** 1202 (374)  **Placebo:**  1051 (337) | 29 | RoP examination | Glucose 30% | Placebo | - Pain - Crying time - Heart rate - Oxygen saturation |
| 29 | Ou Yang* (2012)^34^ | Taiwan | Parallel | **Placebo:**  34.24 (1.58) **Glucose 25%:** 34.69 (1.34) **EBM:**  33.84 (1.43) | **Placebo:**  22 (50%) **Glucose 25%:**  20 (51.3%)  **EBM:**  19 (47.5%) | **Placebo:**  1959 (390) **Glucose 25%:** 2138 (367) **EBM:**  1996 (359) | 123 | Heel-stick | Glucose 25%  EBM | Placebo | - Pain - Crying time - Heart rate - Oxygen saturation - Respiratory rate - Blood pressure - Behavioral response (latency, crying, squeezing) |
| 30 | Pandey* (2013)^35^ | India | Parallel | **Sucrose 24%:** 33.7 (1.3) **Placebo:**  33.3 (2.0) | - | **Sucrose 24%:** 1643.8 (403.5) **Placebo:**  1605.3 (339.6) | 105 | OGT insertion | Sucrose 24% | Placebo | - Pain - Heart rate - Oxygen saturation |
| 31 | Ramar* (2019)^36^ | India | Parallel | **Glucose:**  32 (2) **Placebo:**  32 (2) | **Glucose:**  5 (45%) **Placebo:**  6 (55%) | **Glucose:**  1355.5 (476) **Placebo:**  1334 (361) | 22 | RoP screening | Glucose 25% | Placebo | Pain |
| 32 | Ramenghi* (1996)^37^ | UK | Crosss-over | Median(range): 33 (29-34)  Median (range):^a^ 34 (32 - 34) | - | Median (range): 1980 (1340 - 2520) | 15 | Heel-stick | Sucrose 25% | Placebo | - Pain - Crying time - Heart rate - Behavioural state score |
| 33 | Ramenghi (1999)^38^ | UK | Cross-over | Median (range): **Sucrose 25%:** 35 (-)  **Placebo:**  34 (-) | - | Median (range): **Sucrose 25%:** 2380 (-) **Placebo:**  2410 (-) | 30 | Heel-stick | Sucrose 25% | Placebo | - Crying time - Behaviour score |
| 34 | Ranjbar* (2020)^39^ | Iran | Cross-over | 32.35 (2.81) | 37 (61.66%) | 2173.45 (413.47)^b^ | 46 | Heel-stick | Desxtrose 50% | No treatment | - Pain - Feasibility |
| 35 | Rawal* (2018)^40^ | India | Parallel | **Dex25%:** 35.12 (0.87)  **EBM**: 35.26 (0.80)  **Placebo:**  35.27 (0.86) | **Dex25%:** 12 (57.1%)  **EBM:** 14 (66.7%)  **Placebo:** 11 (52.4%) | **Dextrose 25%:** 2070 (0.35)  **EBM:**  2160 (0.45)  **Placebo:**  2180 (0.38) | 67 | Heel-stick | Dextrose 25% EBM | Placebo | - Pain - Heart rate - Oxygen saturation |
| 36 | Rodrigues* (2017)^41^ | India | Parallel | **Dextrose 25%:**  31.5 (1.82)  **EBM:**  31.65 (1.95) | **Dextrose 25%:**  6 (30%)  **EBM:**  9 (45%) | **Dextrose 25%:** 1415 (367.2)  **EBM:**  1481 (404.36) | 40 | Nasopharyngeal suctioning | Dextrose 25% | EBM | Pain |
| 37 | Sagheb* (2020)^42^ | Iran | Parallel | **Glucose 25%:** 28.9 (2.5) **No treatment:** 29.1 (1.99) **Placebo:**  29.20 (2.14) | **Glucose 25%:**  6 (60%) **No treatment:**  11 (45%) **Placebo:**  5 (50%) | **Glucose 25%:^b^** 1170.9 (291.3)  **No treatment:**  1223.5 (267.8) **Placebo:**  1111 (239.92) | 20 | RoP examination | Glucose 25% Placebo | No treatment | Pain |
| 38 | Sasidharan* (2022)^43^ | India | Parallel | **Dextrose 25%:**  32.6 (1.9) **Sucrose 24%:** 32.8 (1.6) | **Dextrose 25%:** 24 (75%)  **Sucrose 24%:** 14 (44%) | **Dextrose 25%:** 1596 (452)  **Sucrose 24%:** 1651 (350) | 64 | Heel-stick | Dextrose 25% | Sucrose 24% | Pain |
| 39 | Taplak* (2017)^44^ | Turkey | Parallel | - | **EBM:** 9 (45%)  **Sucrose 33%:** 6 (30%)  **Placebo:** 12 (60%) | - | 60 | RoP examination | Sucrose 33%  EBM | Placebo | - Pain - Crying time - Heart rate - Oxygen saturation |
| 40 | Tekgunduz* (2019)^45^ | Turkey | Parallel | **Glucose 30%:** 30.57 (3.47) **No treatment:**  30.26 (3.54) | **Glucose 30%:**  18 (51.4%) **No treatment:** 19 (55.9%) | **Glucose 30%:** 1582.5 (680.06)  **No treatment:**  1460.67 (684.91) | 71 | Nasal continuous positive airway pressure | Glucose 30% | No treatment | - Pain - Heart rate - Oxygen saturation - Heart rate |
| 41 | Uzelli* (2015)^46^ | Turkey | Parallel | **Glucose 5%:** 32.8 (3.0) **No treatment:** 32.3 (2.6) | **Glucose 5%:** 20 (50%)  **No treatment:**  20 (50%) | **Glucose 5%:** 2.170 (0.5)  **No treatment:** 2.270 (0.5) | 80 | IM injection | Glucose 5% | No treatment | - Pain - Crying time - Heart rate - Oxygen saturation |
| 42 | Vezyroglou* (2014)^47^ | Germany | Cross-over | 33.6 (2.1) | 6 (37.5%) | Mean (SD): 1983 (428) | 16 | Oropharyngeal suctioning | Glucose 20% | Placebo | - Pain - Heart rate - Oxygen saturation - Respiratory rate |

Note: GA: gestational age; PMA: post menstrual age; BW: birth weight; CW: current weight; SW: study weight; RoP: Retinopathy of prematurity; OGT: orogastric tube; IM: intramuscular; * eligible for network meta-analysis.

**eTable 5. List of excluded studies**

| **No** | **Author (Year)** | **Country** | **Design** | **Reason** |
| --- | --- | --- | --- | --- |
| 1 | Banga (2016)^48^ | India | Parallel | Did not report outcome of interest |
| 2 | Dili (2014)^49^ | Turkey | Parallel | Using pacifier after giving solution |
| 3 | Grabska (2005)^50^ | USA | Parallel | Using pacifier within the intervention |
| 4 | Ilarslan (2012)^51^ | Turkey | Parallel | Using pacifier before infants receive solution |
| 5 | Silveira (2021)^52^ | Brazil | Crossover | No comparator group |
| 6 | Velumula (2022)^53^ | USA | Parallel | Sweet solution combines with swaddling |

**eTable 6. Characteristics of direct comparison in the network meta-analysis**

| **Outcomes** | **Glucose vs control** | | **Sucrose vs control** | | **EBM vs**  **control** | | **Glucose vs Sucrose** | | **Glucose vs EBM** | | **Sucrose vs**  **EBM** | | **Total** | |
| --- | --- | --- | --- | --- | --- | --- | --- | --- | --- | --- | --- | --- | --- | --- |
|  | Studies | Cases | Studies | Cases | Studies | Cases | Studies | Cases | Studies | Cases | Studies | Cases | Studies | Cases |
| **Pain** |  |  |  |  |  |  |  |  |  |  |  |  |  |  |
| Reaction | 8 | 340 | 7 | 413 | 3 | 168 | 1 | 60 | 2 | 440 | 2 | 112 | 23 | 1533 |
| Regulation | 12 | 634 | 4 | 273 | 4 | 230 | 3 | 218 | 5 | 314 | 0 | 0 | 28 | 1669 |
| Recovery | 7 | 391 | 5 | 266 | 6 | 334 | 3 | 218 | 5 | 314 | 1 | 40 | 27 | 1563 |
| Crying time | 6 | 326 | 4 | 182 | 1 | 84 | 1 | 60 | 1 | 79 | 0 | 0 | 13 | 731 |
| **Heart rate** |  |  |  |  |  |  |  |  |  |  |  |  |  |  |
| Reaction | 3 | 171 | 8 | 332 | 2 | 102 | 1 | 60 | 0 | 0 | 1 | 40 | 15 | 705 |
| Regulation | 1 | 60 | 3 | 168 | 1 | 62 | 1 | 60 | 0 | 0 | 0 | 0 | 6 | 350 |
| Recovery | 4 | 254 | 4 | 150 | 3 | 186 | 1 | 60 | 1 | 79 | 1 | 40 | 14 | 769 |
| **Oxygen saturation** |  |  |  |  |  |  |  |  |  |  |  |  |  |  |
| Reaction | 4 | 251 | 6 | 321 | 2 | 104 | 1 | 60 | 0 | 0 | 1 | 40 | 14 | 776 |
| Regulation | 2 | 140 | 2 | 138 | 1 | 64 | 1 | 60 | 0 | 0 | 0 | 0 | 6 | 402 |
| Recovery | 4 | 254 | **3** | **120** | **3** | **188** | 1 | 60 | 1 | 79 | 1 | 40 | 13 | 741 |
| **Respiratory rate** |  |  |  |  |  |  |  |  |  |  |  |  |  |  |
| Reaction | 2 | 100 | 3 | 152 | 0 | 0 | 1 | 60 | 0 | 0 | 0 | 0 | 6 | 312 |
| Recovery | 3 | 183 | 2 | 80 | 1 | 84 | 1 | 60 | 1 | 79 | 0 | 0 | 8 | 486 |
| **Adverse Event** | 6 | 434 | 3 | 255 | 2 | 106 | 2 | 157 | 2 | 155 | 0 | 0 | 15 | 1107 |

**eTable 7. Treatment ranking probability using P-Score**

| **Phase** | **Glucose** | **Sucrose** | **EBM** | **Control** |
| --- | --- | --- | --- | --- |
| **Pain** |  | | | |
| During | 0.8765 | 0.7132 | 0.329 | 0.0813 |
| Regulation | 0.9166 | 0.5447 | 0.5379 | 0.0007 |
| Recovery | 0.8854 | 0.5128 | 0.6016 | 0.0002 |
| **Crying time** | 0.6028 | 0.8912 | 0.4013 | 0.1048 |
| **Heart rate** |  | | | |
| During | 0.6162 | 0.4818 | 0.4794 | 0.4225 |
| Regulation | 0.7471 | 0.4481 | 0.7357 | 0.0691 |
| Recovery | 0.5012 | 0.4383 | 0.8981 | 0.1625 |
| **Oxygen Saturation** |  | | | |
| During | 0.58 | 0.5986 | 0.4444 | 0.377 |
| Regulation | 0.5316 | 0.2553 | 0.7559 | 0.4572 |
| Recovery | 0.5121 | 0.0662 | 0.9886 | 0.4332 |
| **Respiratory rate** |  | | | |
| During | 0.3339 | 0.5085 | NA | 0.6576 |
| Recovery | 0.5532 | 0.3737 | 0.7941 | 0.279 |
| **Adverse event** | 0.3339 | 0.5085 | NA | 0.6576 |

**eTable 8. Assessment of heterogeneity**

**A.** Heterogeneity in main analysis

| **Outcomes** | **Between study variance (τ^2^)** | **Heterogeneity assessment (I^2^)** |
| --- | --- | --- |
| **Pain** |  |  |
| During | 0.4196 | 84.6% |
| Regulation | 0.3224 | 81% |
| Recovery | 0.1263 | 63.1% |
| **Crying time** | 99.3414 | 92.8% |
| **Heart rate** |  |  |
| During | 1.4086 | 13.9% |
| Regulation | 0 | 0% |
| Recovery | 7.3433 | 27.1% |
| **Oxygen saturation** |  |  |
| During | 1.8602 | 50.3% |
| Regulation | 3.4706 | 81.5% |
| Recovery | 0 | 0% |
| **Respiratory rate** |  |  |
| During | 13.3827 | 41.9% |
| Recovery | 0 | 0% |
| **Adverse event** | 0 | 0% |

**B.** Heterogeneity in sensitivity analysis

| **Outcomes** | **Between study variance (τ^2^)** | **Heterogeneity assessment (I^2^)** |
| --- | --- | --- |
| **Pain** |  |  |
| During | 0.4103 | 82.7% |
| Regulation | 0.3442 | 81.9% |
| Recovery | - | - |
| **Crying time** | 175.2817 | 94.1% |
| **Heart rate** |  |  |
| During | 2.3040 | 21.6% |
| Regulation | 7.4112 | 30.2% |
| Recovery | 19.7319 | 50.4% |
| **Oxygen saturation** |  |  |
| During | 0.7163 | 28.7% |
| Regulation | 0.2363 | 24.1% |
| Recovery | 0 | 0% |
| **Respiratory rate** |  |  |
| During | NA | NA |
| Recovery | 4.2008 | 19.4% |
| **Adverse event** | 0 | 0% |

**eTable 9. Local consistency: split of direct and indirect evidence**

| **Comparison** | **k** | **NMA** | **Direct** | **Indirect** | **Diff** | **Diff_95CI** | | ***p*-Value** |
| --- | --- | --- | --- | --- | --- | --- | --- | --- |
|  |  |  |  |  |  | **lower** | **upper** |  |
| **Pain (reaction)** | | | | | | | | |
| EBM:Control | 3 | -0.22 | -0.17 | -0.27 | 0.10 | -1.06 | 1.27 | 0.86 |
| Glucose:Control | 8 | -0.72 | -0.69 | -0.84 | 0.15 | -1.07 | 1.36 | 0.81 |
| Sucrose:Control | 7 | -0.56 | -0.52 | -0.81 | 0.29 | -1.13 | 1.71 | 0.69 |
| EBM:Glucose | 2 | 0.50 | 0.57 | 0.44 | 0.13 | -1.14 | 1.41 | 0.84 |
| EBM:Sucrose | 2 | 0.34 | 0.22 | 0.44 | -0.22 | -1.54 | 1.10 | 0.75 |
| Glucose:Sucrose | 1 | -0.16 | 0.15 | -0.24 | 0.40 | -1.14 | 1.93 | 0.61 |
| **Pain (regulation)** | | | | | | | | |
| EBM:Control | 4 | -0.80 | -0.94 | -0.52 | -0.42 | -1.49 | 0.65 | 0.45 |
| Glucose:Control | 12 | -1.09 | -1.11 | -1.03 | -0.08 | -1.03 | 0.88 | 0.87 |
| Sucrose:Control | 4 | -0.80 | -0.64 | -1.12 | 0.48 | -0.61 | 1.56 | 0.39 |
| EBM:Glucose | 5 | 0.30 | 0.36 | 0.09 | 0.27 | -0.90 | 1.45 | 0.65 |
| EBM:Sucrose | 0 | 0.00 | NA | 0.00 | NA | NA | NA | NA |
| Glucose:Sucrose | 3 | -0.30 | -0.14 | -0.49 | 0.35 | -0.71 | 1.40 | 0.52 |
| **Pain (recovery)** | | | | | | | | |
| EBM:Control | 6 | -0.62 | -0.66 | -0.50 | -0.15 | -0.92 | 0.62 | 0.70 |
| Glucose:Control | 7 | -0.76 | -0.76 | -0.77 | 0.01 | -0.64 | 0.67 | 0.97 |
| Sucrose:Control | 5 | -0.57 | -0.63 | -0.42 | -0.21 | -0.96 | 0.55 | 0.60 |
| EBM:Glucose | 5 | 0.14 | 0.15 | 0.13 | 0.02 | -0.70 | 0.74 | 0.96 |
| EBM:Sucrose | 1 | -0.06 | -0.28 | 0.00 | -0.28 | -1.33 | 0.76 | 0.60 |
| Glucose:Sucrose | 3 | -0.20 | -0.15 | -0.26 | 0.11 | -0.62 | 0.84 | 0.78 |
| **Crying time** | | | | | | | | |
| EBM:Control | 1 | -6.60 | -27.36 | 25.28 | -52.64 | -106.15 | 0.87 | 0.05 |
| Glucose:Control | 6 | -12.31 | -13.77 | 24.14 | -37.91 | -84.66 | 8.84 | 0.11 |
| Sucrose:Control | 4 | -19.53 | -19.30 | -22.32 | 3.02 | -39.74 | 45.78 | 0.89 |
| EBM:Glucose | 1 | 5.71 | 14.89 | -63.06 | 77.95 | -1.29 | 157.20 | 0.05 |
| EBM:Sucrose | 0 | 12.92 | NA | 12.92 | NA | NA | NA | NA |
| Glucose:Sucrose | 1 | 7.22 | 15.15 | 1.39 | 13.76 | -13.04 | 40.56 | 0.31 |
| **Heart rate (reaction)** | | | | | | | | |
| EBM:Control | 2 | -0.15 | 0.83 | -25.42 | 26.25 | -7.57 | 60.07 | 0.13 |
| Glucose:Control | 3 | -0.99 | 0.22 | -8.28 | 8.50 | -6.15 | 23.15 | 0.26 |
| Sucrose:Control | 8 | -0.16 | -0.26 | 9.24 | -9.50 | -27.22 | 8.22 | 0.29 |
| EBM:Glucose | 0 | 0.84 | NA | 0.84 | NA | NA | NA | NA |
| EBM:Sucrose | 1 | 0.01 | -6.83 | 2.58 | -9.41 | -24.22 | 5.41 | 0.21 |
| Glucose:Sucrose | 1 | -0.83 | -5.00 | 2.19 | -7.19 | -17.83 | 3.46 | 0.19 |
| **Heart rate (regulation)** | | | | | | | | |
| EBM:Control | 1 | -6.50 | -6.50 | NA | NA | NA | NA | NA |
| Glucose:Control | 1 | -6.56 | -10.00 | 2.90 | -12.90 | -31.75 | 5.95 | 0.18 |
| Sucrose:Control | 3 | -3.10 | -3.10 | NA | NA | NA | NA | NA |
| EBM:Glucose | 0 | 0.06 | NA | 0.06 | NA | NA | NA | NA |
| EBM:Sucrose | 0 | -3.40 | NA | -3.40 | NA | NA | NA | NA |
| Glucose:Sucrose | 1 | -3.46 | -1.00 | -16.02 | 15.02 | -6.92 | 36.97 | 0.18 |
| **Heart rate (recovery)** | | | | | | | | |
| EBM:Control | 3 | -5.23 | -4.47 | -11.83 | 7.36 | -9.01 | 23.73 | 0.38 |
| Glucose:Control | 4 | -2.07 | -2.55 | 0.60 | -3.15 | -17.28 | 10.97 | 0.66 |
| Sucrose:Control | 4 | -1.64 | -0.58 | -8.40 | 7.82 | -6.48 | 22.12 | 0.28 |
| EBM:Glucose | 1 | -3.16 | -0.80 | -5.22 | 4.42 | -8.00 | 16.84 | 0.49 |
| EBM:Sucrose | 1 | -3.59 | -6.00 | -1.37 | -4.63 | -16.98 | 7.71 | 0.46 |
| Glucose:Sucrose | 1 | -0.44 | 4.00 | -3.78 | 7.78 | -4.61 | 20.16 | 0.22 |
| **Respiratory rate (reaction)** | | | | | | | | |
| Glucose:Control | 2 | 1.98 | 2.28 | -2.90 | 5.18 | -23.21 | 33.58 | 0.72 |
| Sucrose:Control | 3 | 0.81 | -0.23 | 12.83 | -13.05 | -37.67 | 11.56 | 0.30 |
| Glucose:Sucrose | 1 | 1.17 | -2.00 | 5.84 | -7.84 | -24.40 | 8.72 | 0.35 |
| **Respiratory rate (regulation)** | | | | | | | | |
| EBM:Control | 1 | -2.93 | -3.20 | -1.62 | -1.58 | -15.46 | 12.30 | 0.82 |
| Glucose:Control | 3 | -1.33 | -1.60 | 7.19 | -8.78 | -29.40 | 11.84 | 0.40 |
| Sucrose:Control | 2 | -0.22 | -1.06 | 5.00 | -6.06 | -22.52 | 10.39 | 0.47 |
| EBM:Glucose | 1 | -1.60 | -1.50 | -4.32 | 2.82 | -21.93 | 27.57 | 0.82 |
| EBM:Sucrose | 0 | -2.71 | NA | -2.71 | NA | NA | NA | NA |
| Glucose:Sucrose | 1 | -1.10 | -1.00 | -1.28 | 0.28 | -12.03 | 12.60 | 0.96 |
| **Oxygen saturation (reaction)** | | | | | | | | |
| EBM:Control | 2 | 0.05 | -0.17 | 4.63 | -4.80 | -17.51 | 7.91 | 0.46 |
| Glucose:Control | 4 | 0.40 | 0.24 | 4.40 | -4.17 | -13.95 | 5.61 | 0.40 |
| Sucrose:Control | 6 | 0.41 | 0.20 | 4.98 | -4.78 | -12.50 | 2.95 | 0.23 |
| EBM:Glucose | 0 | -0.35 | NA | -0.35 | NA | NA | NA | NA |
| EBM:Sucrose | 1 | -0.37 | 0.95 | -1.01 | 1.96 | -4.37 | 8.28 | 0.54 |
| Glucose:Sucrose | 1 | -0.01 | -1.00 | 0.53 | -1.53 | -6.39 | 3.32 | 0.54 |
| **Oxygen saturation (regulation)** | | | | | | | | |
| EBM:Control | 1 | 1.50 | 1.50 | NA | NA | NA | NA | NA |
| Glucose:Control | 2 | 0.24 | 0.20 | 0.83 | -0.63 | -11.76 | 10.50 | 0.91 |
| Sucrose:Control | 2 | -0.75 | -1.21 | 7.27 | -8.49 | -20.52 | 3.54 | 0.17 |
| EBM:Glucose | 0 | 1.26 | NA | 1.26 | NA | NA | NA | NA |
| EBM:Sucrose | 0 | 2.25 | NA | 2.25 | NA | NA | NA | NA |
| Glucose:Sucrose | 1 | 0.99 | 0.00 | 2.62 | -2.62 | -9.60 | 4.35 | 0.46 |
| **Oxygen saturation (recovery)** | | | | | | | | |
| EBM:Control | 3 | 1.72 | 1.73 | 1.65 | 0.08 | -3.67 | 3.82 | 0.97 |
| Glucose:Control | 4 | 0.16 | 0.26 | -1.34 | 1.60 | -3.20 | 6.41 | 0.51 |
| Sucrose:Control | 3 | -0.94 | -1.00 | 0.80 | -1.79 | -10.21 | 6.62 | 0.68 |
| EBM:Glucose | 1 | 1.56 | 1.50 | 1.58 | -0.08 | -3.69 | 3.54 | 0.97 |
| EBM:Sucrose | 1 | 2.66 | 2.75 | 2.65 | 0.10 | -5.42 | 5.63 | 0.97 |
| Glucose:Sucrose | 1 | 1.11 | 1.00 | 1.33 | -0.33 | -3.94 | 3.28 | 0.86 |
| **Adverse effect** | | | | | | | | |
| EBM:Control | 1 | 0.39 | -1.10 | 0.64 | -1.74 | -5.14 | 1.66 | 0.32 |
| Glucose:Control | 4 | 0.44 | 0.42 | 0.89 | -0.46 | -2.77 | 1.84 | 0.69 |
| Sucrose:Control | 2 | -0.19 | -0.06 | -0.94 | 0.88 | -1.51 | 3.26 | 0.47 |
| EBM:Glucose | 2 | -0.05 | -0.04 | -1.16 | 1.12 | -11.42 | 13.66 | 0.86 |
| EBM:Sucrose | 0 | 0.57 | NA | 0.57 | NA | NA | NA | NA |
| Glucose:Sucrose | 2 | 0.62 | 1.35 | 0.48 | 0.88 | -1.51 | 3.26 | 0.47 |

Note:

k - Number of studies providing direct evidence; NMA - Estimated treatment effect (SMD/MD) in network meta-analysis; Direct - Estimated treatment effect (SMD/MD) derived from direct evidence; Indirect - Estimated treatment effect (SMD/MD) derived from indirect evidence; Diff - Difference between direct and indirect treatment estimates; Diff_95CI - Confidence interval of difference between direct and indirect treatment estimates; *p*-Value - p-value of test for direct – indirect disagreement.

**eTable 10. Assessment of global inconsistency**

**A.** Assessment of global inconsistency in the main network meta-analysis

| **Outcome** | **Q** | **df** | ***p-value*** |
| --- | --- | --- | --- |
| **Pain** |  |  |  |
| During | 17.51 | 6 | **0.0076** |
| Regulation | 14.46 | 6 | 0.0249 |
| Recovery | 23.54 | 8 | **0.0027** |
| **Crying time** | 84.61 | 3 | <0.0001 |
| **Heart rate** |  |  |  |
| During | 3.72 | 4 | 0.4457 |
| Regulation | 1.80 | 1 | 0.1797 |
| Recovery | 9.15 | 6 | 0.1653 |
| **Oxygen saturation** |  |  |  |
| During | 12.5 | 4 | 0.017 |
| Regulation | 10.8 | 2 | **0.0045** |
| Recovery | 1.47 | 6 | 0.9614 |
| **Respiratory rate** |  |  |  |
| During | 2.22 | 2 | 0.3296 |
| Recovery | 2.53 | 3 | 0.4697 |
| **Adverse event** | 1.8 | 3 | 0.6154 |

Bold = Significant consistency improvement from the main analysis to sensitivity analysis

**B.** Assessment of global inconsistency in the sensitivity analysis

| **Outcome** | **Q** | **df** | ***p-value*** |
| --- | --- | --- | --- |
| **Pain** |  |  |  |
| During | 7.49 | 6 | **0.2777** |
| Regulation | 14.71 | 6 | 0.0266 |
| Recovery | 4.14 | 5 | **0.529** |
| **Crying time** | 75.71 | 2 | <0.0001 |
| **Heart rate** |  |  |  |
| During | 3.71 | 4 | 0.4460 |
| Regulation | 1.43 | 1 | 0.2312 |
| Recovery | 8.81 | 4 | 0.0660 |
| **Oxygen saturation** |  |  |  |
| During | 10.48 | 4 | 0.0330 |
| Regulation | 1.32 | 1 | **0.2510** |
| Recovery | 0.35 | 4 | 0.9866 |
| **Respiratory rate** |  |  |  |
| During | NA | NA | NA |
| Recovery | 2.48 | 2 | 0.2892 |
| **Adverse event** | 0.54 | 1 | 0.4631 |

Bold = Significant consistency improvement from the main analysis to sensitivity analysis

**eTable 11. Full design-by-treatment interaction random effects model**

**A.** Consistency under the assumption of a full design-by-treatment interaction random effects model

| **Outcome** | **Q** | **df** | ***p -* Value** |
| --- | --- | --- | --- |
| **Pain** |  |  |  |
| During | 1.23 | 6 | 0.9754 |
| Regulation | 2.33 | 6 | 0.8869 |
| Recovery | 7.01 | 8 | 0.5355 |
| **Crying time** | 13.06 | 3 | **0.0045** |
| **Heart rate** |  |  |  |
| During | 4.35 | 4 | 0.3612 |
| Regulation | 1.80 | 1 | 0.1797 |
| Recovery | 9.15 | 6 | 0.1653 |
| **Oxygen saturation** |  |  |  |
| During | 9.08 | 4 | 0.0591 |
| Regulation | 10.80 | 2 | 0.0045 |
| Recovery | 1.47 | 6 | 0.964 |
| **Respiratory rate** |  |  |  |
| During | 0.37 | 2 | 0.8307 |
| Recovery | 2.53 | 3 | 0.4697 |
| **Adverse event** | 1.80 | 3 | 0.6154 |

Bold = Significant consistency improvement from main analysis to sensitivity analysis

**B.** Consistency of full design-by-treatment in the sensitivity analysis

| **Phase** | **Q** | **df** | ***p -* Value** |
| --- | --- | --- | --- |
| **Pain** |  |  |  |
| During | 1.35 | 6 | 0.9688 |
| Regulation | 2.21 | 6 | 0.8994 |
| Recovery | 1.13 | 5 | 0.9517 |
| **Crying time** | 2.65 | 2 | **0.2658** |
| **Heart rate** |  |  |  |
| During | 4.32 | 4 | 0.366 |
| Regulation | 1.43 | 2 | 0.2312 |
| Recovery | 5.45 | 4 | 0.2442 |
| **Oxygen saturation** |  |  |  |
| During | 10.48 | 4 | 0.0330 |
| Regulation | 1.32 | 1 | 0.2510 |
| Recovery | 0.35 | 4 | 0.9866 |
| **Respiratory rate** |  |  |  |
| During | NA | NA | NA |
| Recovery | 2.48 | 2 | 0.2892 |
| **Adverse event** | 0.54 | 1 | 0.4631 |

Bold = Significant consistency improvement from main analysis to sensitivity analysis

**eTable 12. Sensitivity analysis**

Comparison should be interpreted left to right with SMD <0 indicating favour to interventions for all of outcomes, except oxygen saturation. Significant results are exhibited in the bold fonts.

**A. Pain**

**(1)** Pain level in regulation phase by excluding extreme concentration of sweet solution (Deghani, 2019)

| Glucose | -0.14 [-0.86;  0.57] | -0.36 [-0.93;  0.21] | -1.13 [-1.53; -0.73] |
| --- | --- | --- | --- |
| -0.31 [-0.85;  0.23] | Sucrose | . | -0.64 [-1.28; -0.01] |
| -0.30 [-0.81;  0.20] | 0.00 [-0.69;  0.70] | EBM | -0.94 [-1.58; -0.30] |
| **-1.11 [-1.48; -0.75]** | -0.81 [-1.33; -0.28] | -0.81 [-1.33; -0.28] | Control |

**(2)** Pain level in recovery phase after excluding Ramenghi 1996 (high risk of bias in a group detecting higher heterogeneity)

| Glucose | -0.15 [-0.54;  0.25] | -0.15 [-0.64;  0.34] | -0.76 [-1.11; -0.40] |
| --- | --- | --- | --- |
| -0.14 [-0.47;  0.20] | EBM | -0.28 [-1.23;  0.66] | -0.66 [-1.02; -0.29] |
| -0.22 [-0.60;  0.16] | -0.08 [-0.52;  0.35] | Sucrose | -0.59 [-1.04; -0.13] |
| **-0.75 [-1.05; -0.45]** | **-0.62 [-0.94; -0.29]** | **-0.53 [-0.90; -0.16]** | Control |

**B. Crying time**

**(3)** Total crying time by excluding high risk of bias study in the inconsistency group (Glucose VS Control): Ou Yang 2012, Uzelli & Yapucu 2019.

| Sucrose | -15.15 [-41.72; 11.42] | -19.69 [-34.63; -4.74] |
| --- | --- | --- |
| -8.18 [-26.24;  9.87] | Glucose | -13.47 [-27.59;  0.66] |
| **-19.72 [-34.18; -5.27]** | -11.54 [-25.28;  2.20] | Control |

[**C. Heart rate**](#_Toc53220847)

**(4)** Heart rate in reactivity phase procedure after excluding Ramenghi 1996 (high risk of bias in a group detecting higher heterogeneity)

| Glucose | -5.00 [-13.32;  3.32] | . | 0.23 [ -5.39;  5.86] |
| --- | --- | --- | --- |
| -0.76 [-6.18; 4.66] | Sucrose | 6.83 [ -5.93; 19.59] | -0.35 [ -2.53;  1.82] |
| -0.79 [-9.13; 7.56] | -0.03 [-6.82; 6.77] | EBM | 0.83 [ -5.83;  7.49] |
| -0.98 [-6.20; 4.25] | -0.22 [-2.38; 1.95] | -0.19 [-6.72; 6.34] | Control |

**(5)** Heart rate in the regulation phase after excluding Ramenghi 1996 (high risk of bias in a group detecting higher heterogeneity)

| Glucose | . | -1.00 [-11.36;  9.36] | -10.00 [-21.09;  1.09] |
| --- | --- | --- | --- |
| -0.91 [-14.66; 12.84] | EBM | . | -6.50 [-16.07;  3.07] |
| -3.00 [-12.59;  6.58] | -2.09 [-13.68;  9.49] | Sucrose | -4.41 [-10.92;  2.11] |
| -7.41 [-17.28;  2.45] | -6.50 [-16.07;  3.07] | -4.41 [-10.92;  2.11] | Control |

**(6)** Heart rate in the recovery phase after excluding Ou Yang 2012, and Ramenghi 1996 (high risk of bias in a group detecting higher heterogeneity)

| EBM | -6.00 [-17.26;  5.26] | . | -5.21 [-13.22;  2.81] |
| --- | --- | --- | --- |
| -4.40 [-13.25;  4.44] | Sucrose | -4.00 [-15.62;  7.62] | -1.20 [ -8.19;  5.80] |
| -5.02 [-15.37;  5.34] | -0.61 [ -9.16;  7.94] | Glucose | -2.82 [-10.62;  4.99] |
| -6.80 [-14.53;  0.92] | -2.40 [ -8.95;  4.14] | -1.79 [ -9.17;  5.59] | Control |

**D. Oxygen saturation**

**(7)** Oxygen saturation in reactivity phase after removing Uzelli & Yapucu 2019 (high risk of bias in a group detecting higher heterogeneity)

| Sucrose | -0.95 [-5.69; 3.79] | 0.26 [-1.01; 1.52] | 1.00 [-2.28; 4.28] |
| --- | --- | --- | --- |
| 0.39 [-2.03; 2.81] | EBM | -0.18 [-2.37; 2.01] | . |
| 0.37 [-0.88; 1.61] | -0.02 [-2.17; 2.13] | Control | 0.65 [-1.02; 2.32] |
| 0.80 [-1.16; 2.76] | 0.41 [-2.29; 3.11] | 0.43 [-1.21; 2.07] | Glucose |

**(8)** Oxygen saturation in regulation phase after removing Uzelli & Yapucu 2019 (high risk of bias)

| EBM | 1.50 [-0.59; 3.59] | . | . |
| --- | --- | --- | --- |
| 1.50 [-0.59;  3.59] | Control | 0.93 [-0.24; 2.10] | 2.00 [-0.15; 4.15] |
| **2.43 [ 0.04;  4.83]** | 0.93 [-0.24;  2.10] | Sucrose | -0.00 [-2.44; 2.44] |
| **3.09 [ 0.20;  5.98]** | 1.59 [-0.40;  3.58] | 0.66 [-1.41;  2.72] | Glucose |

**(9)** Oxygen saturation in regulation phase after removing Ou Yang 2012 (high risk of bias)

| EBM | . | 1.42 [-0.07; 2.90] | 2.75 [-2.40; 7.90] |
| --- | --- | --- | --- |
| 1.32 [-0.60;  3.24] | Glucose | 0.13 [-1.11; 1.37] | 1.00 [-1.05; 3.05] |
| 1.45 [-0.03;  2.92] | 0.13 [-1.11;  1.37] | Control | 1.00 [-0.44; 2.43] |
| **2.41 [ 0.40;  4.41]** | 1.09 [-0.64;  2.82] | 0.96 [-0.46;  2.38] | Sucrose |

**E. Respiratory rate**

**(10)** During procedure

#SA not perform:

- no extreme concentration

- no heterogeneity

- no inconsistency

- no pub. bias

- no study with high RoB

**(11)** Respiratory rate in recovery phase after removing Ou Yang 2012 (high risk of bias)

| Glucose | -1.00 [-9.52; 7.52] | -1.42 [-7.54; 4.71] |
| --- | --- | --- |
| -0.82 [-8.03; 6.40] | Sucrose | -0.97 [-7.74; 5.80] |
| -0.82 [-6.74; 5.09] | -0.01 [-6.50; 6.49] | Control |

**F. Adverse effect**

**(12).** Adverse efect after removing high risk of bias study (Carbajal 2002, Rawal 2018)

| Sucrose | 0.94 [0.38;  2.34] | . | 0.26 [0.03;  2.29] |
| --- | --- | --- | --- |
| 0.83 [0.36; 1.92] | Control | 3.00 [0.13; 69.61] | 0.67 [0.47;  0.96] |
| 0.57 [0.13; 2.47] | 0.70 [0.21; 2.32] | EBM | 0.96 [0.30;  3.07] |
| 0.55 [0.22; 1.33] | **0.66 [0.47; 0.94]** | 0.95 [0.30; 3.03] | Glucose |

**eTable 13. Meta-regression**

| **Variables** | **Coefficient** | **(95% CI)** | **DIC** | **I2 (%)** |
| --- | --- | --- | --- | --- |
| Pain during |  |  | 41.328 | 16% |
| Heel stick | -0.72 | -1.88 to 0.45 | 26.852 | 14% |
| High risk of bias | -1.87* | -3.35 to -0.46 | 18.158 | 13% |
| Pain regulation |  |  | 43.123 | 15% |
| Heel stick | -0.70 | -1.93 to 0.45 | 27.050 | 14% |
| High risk of bias | -1.87* | -3.40 to -0.46 | 19.370 | 13% |

*Significantly associated with pain reduction during and regulation phase

**eFigure 1. The mean path length**


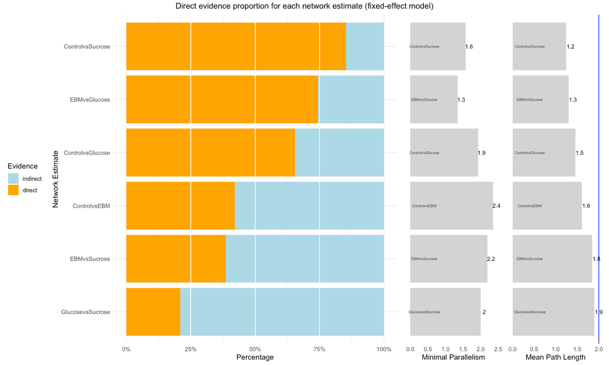

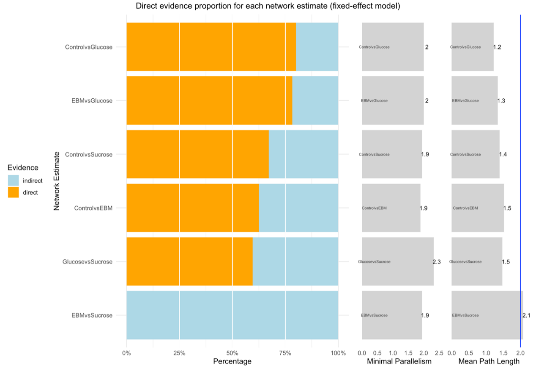

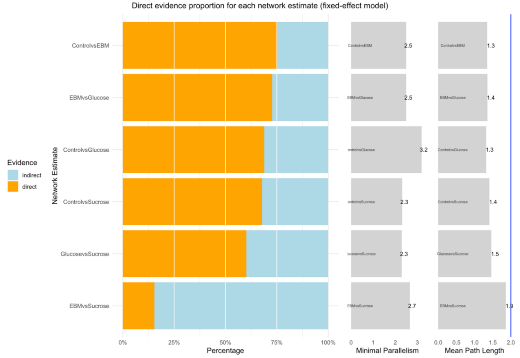


1

2

3

**A.** League direct evidence proportion of (1) pain reactivity, (2) pain regulation, and (3) pain recovery

**
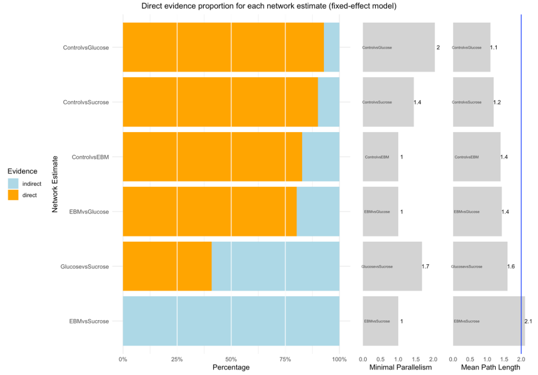
**

**B.** League direct evidence proportion of overall crying time


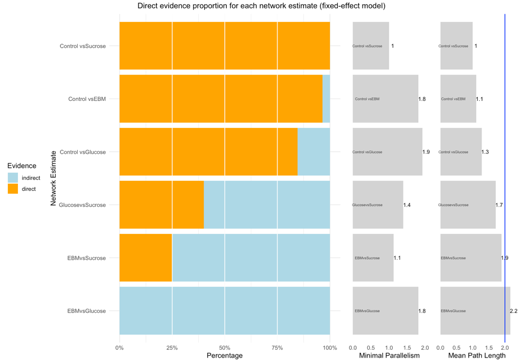


1


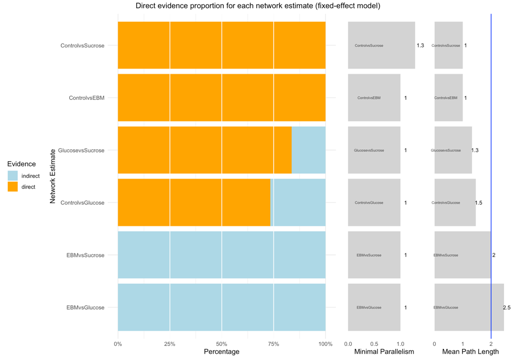

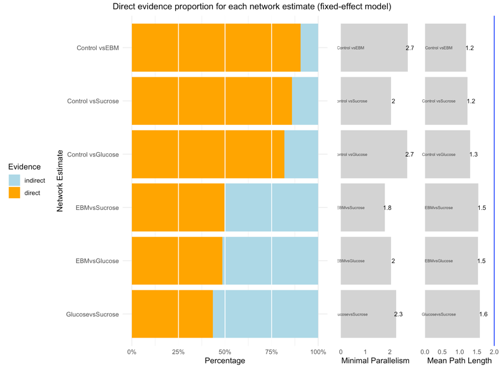


2

3

**C.** League direct evidence portion of heart rate in (1) reactivity, (2) regulation, and (3) recovery phase


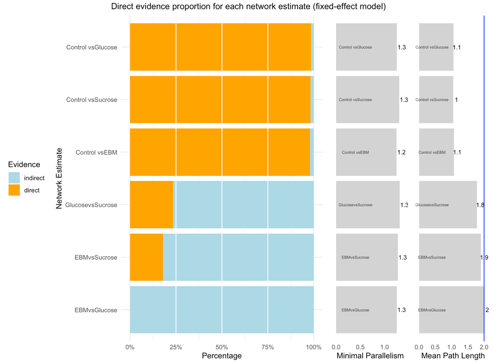

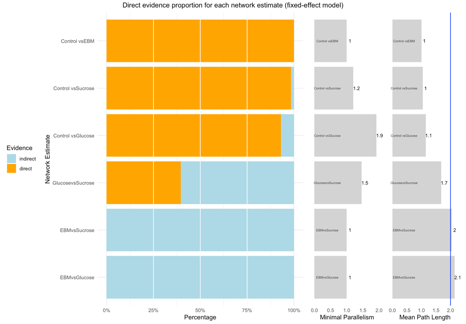

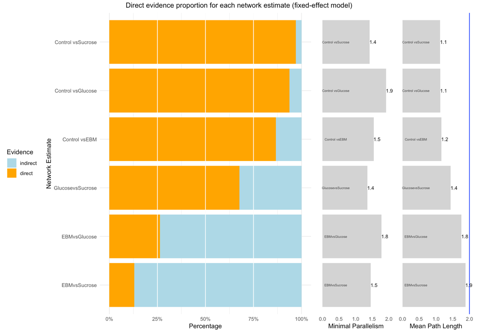


2

1

3

**D.** League direct evidence portion of oxygen saturation in (1) reactivity, (2) regulation, and (3) recovery phase


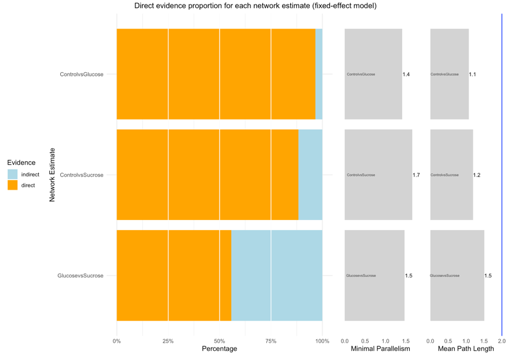

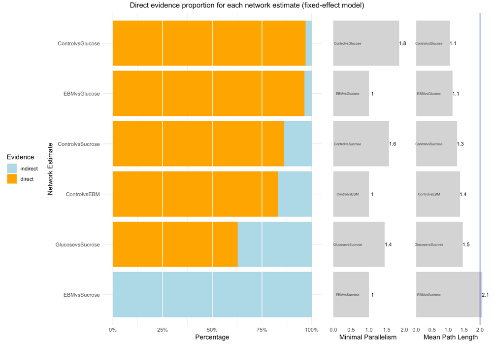


1

2

**E.** League direct evidence portion of respiratory rate in (1) reactivity, and (2) recovery phase


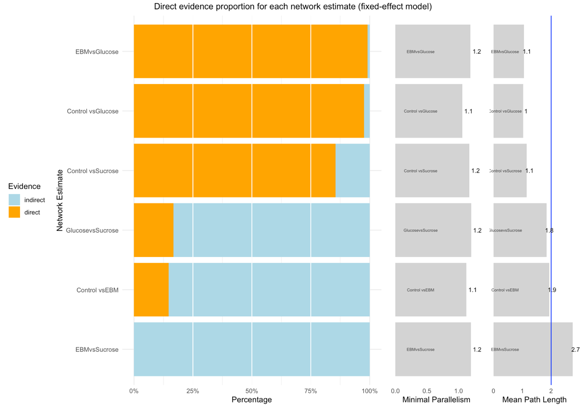


**F.** League direct evidence proportion of overall adverse effect

**eFigure 2. Risk of bias**


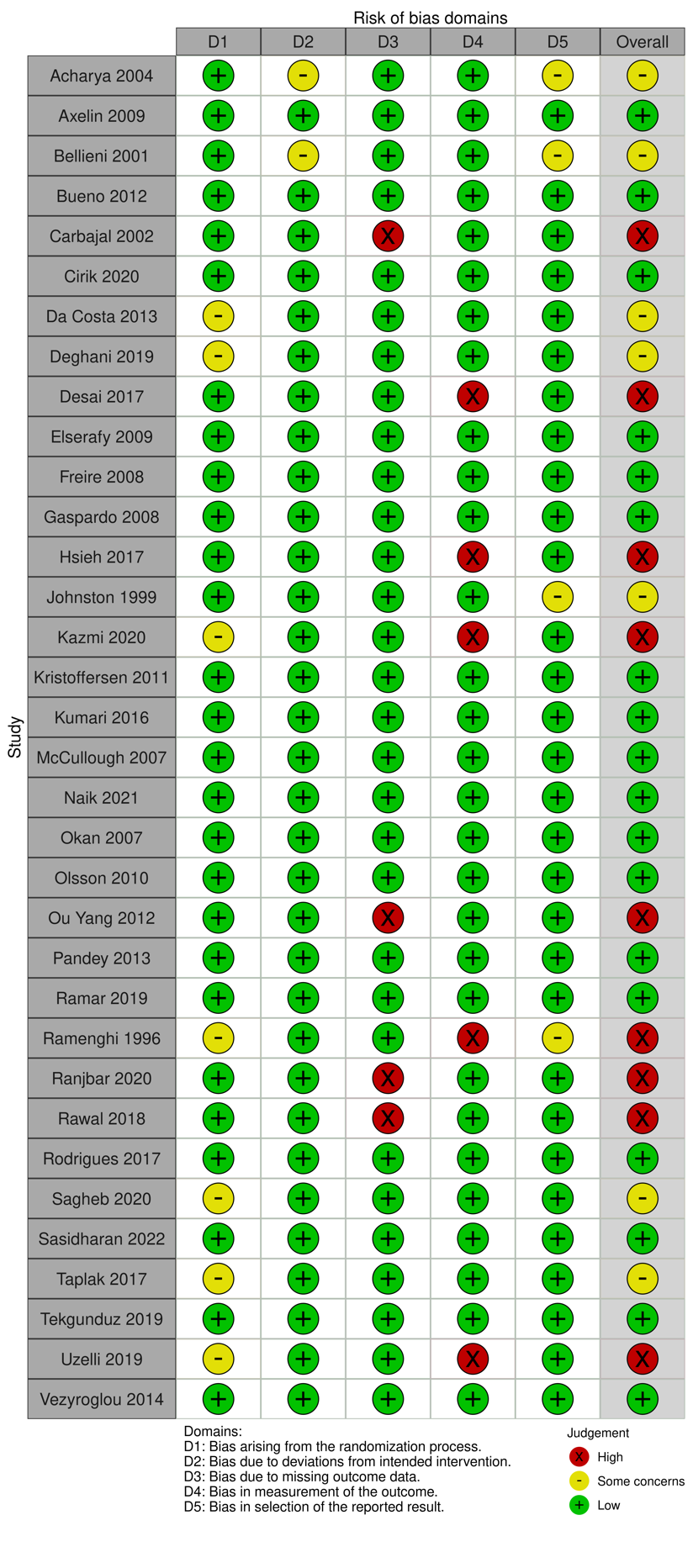


**A.** Risk of bias assessment in pain trials


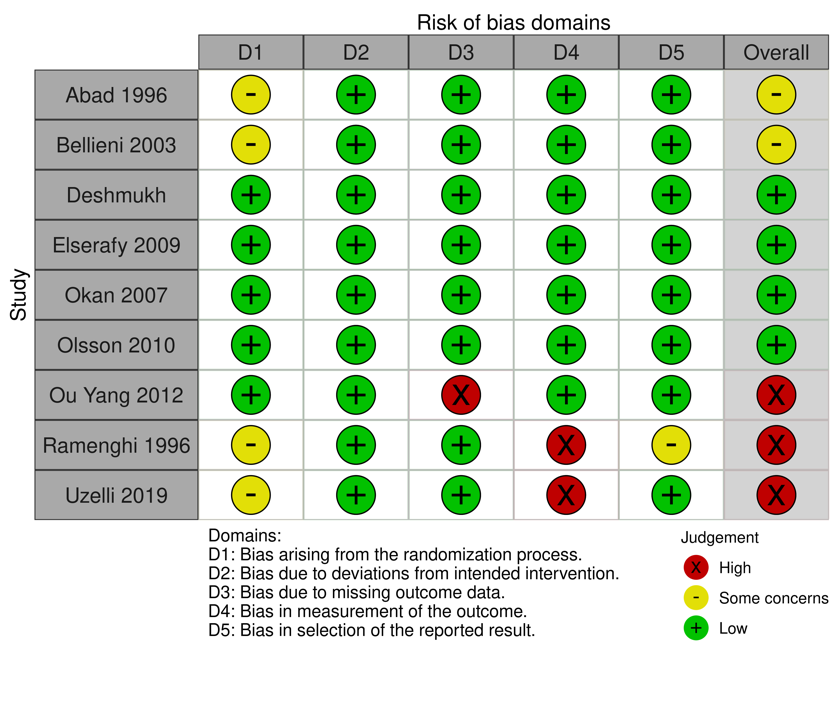


**B.** Risk of bias assessment in the total crying time

**
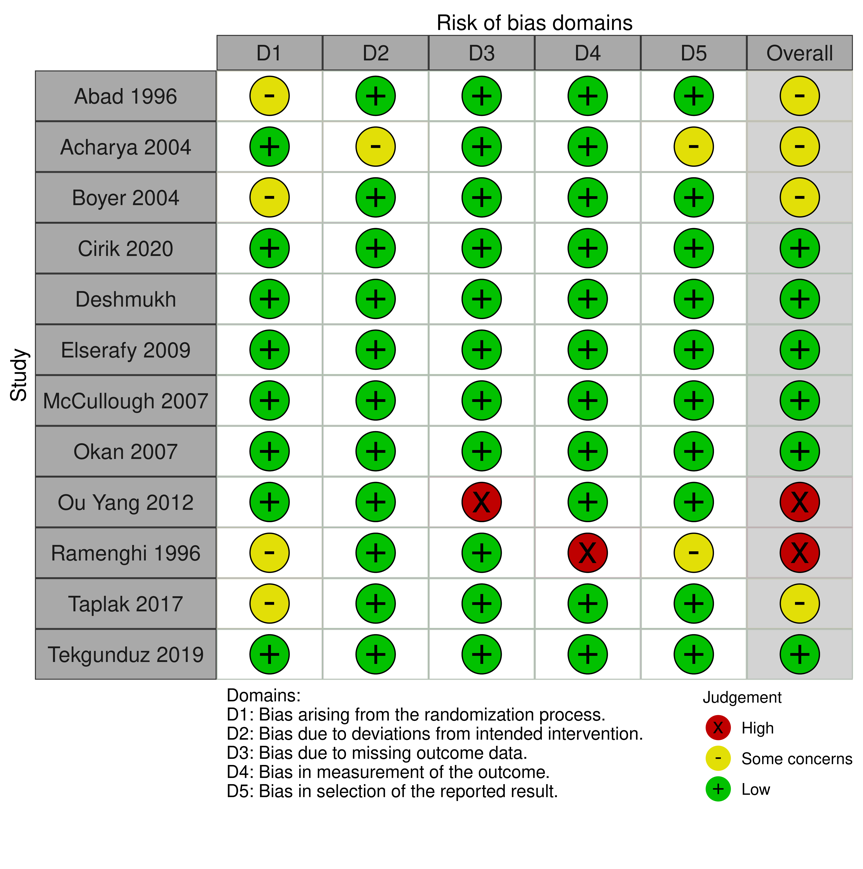
**

**C.** Risk of bias assessment in heart rate outcome

**
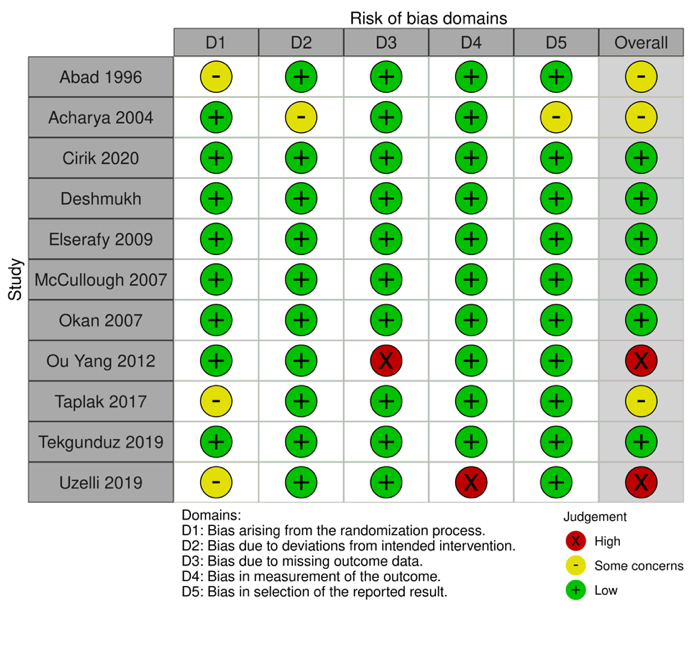
**

**D.** Risk of bias assessment for oxygen saturation

**
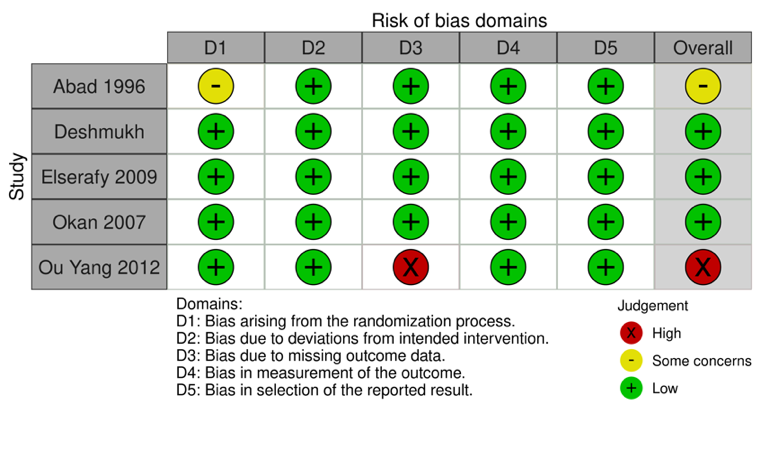
**

**E.** Risk of bias assessment for respiratory rate

**
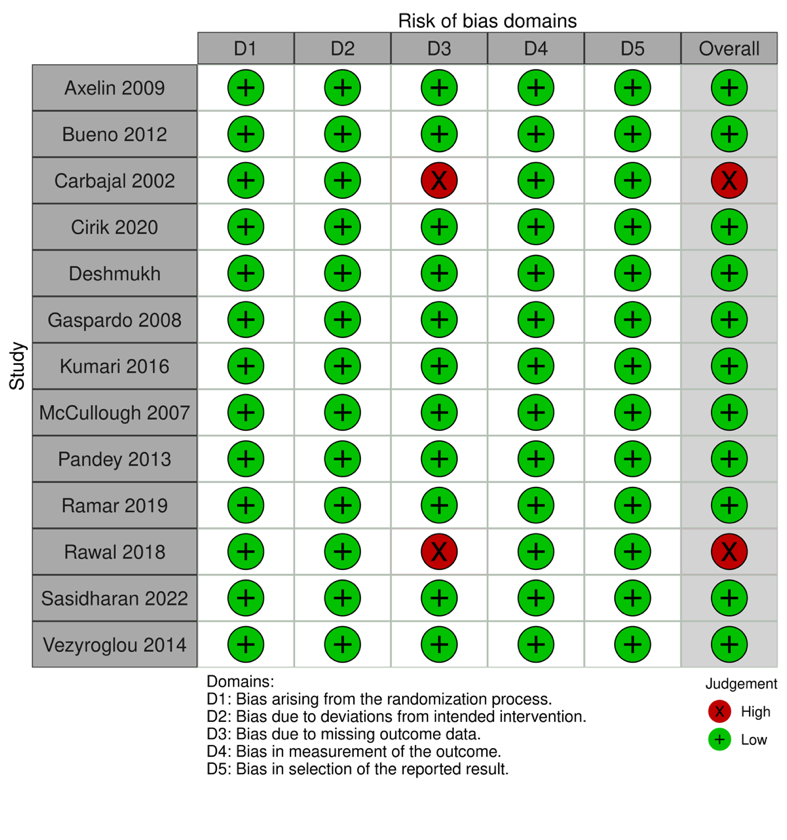
**

**F.** Risk of bias assessment for adverse event

**
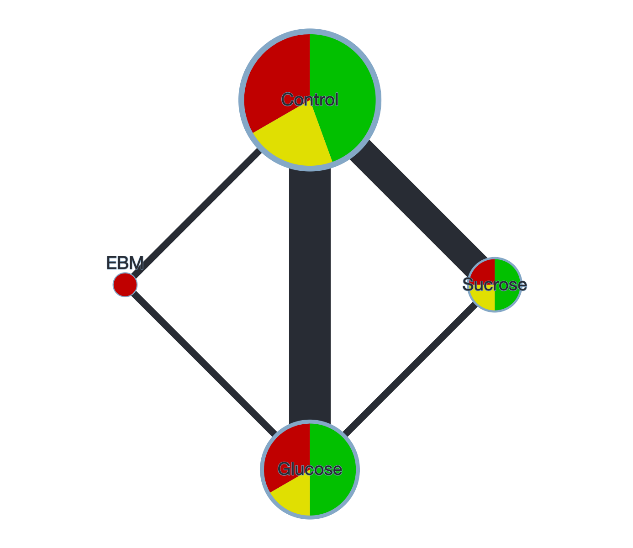
eFigure 3. Network geometry**

A. Network geometry for total crying time


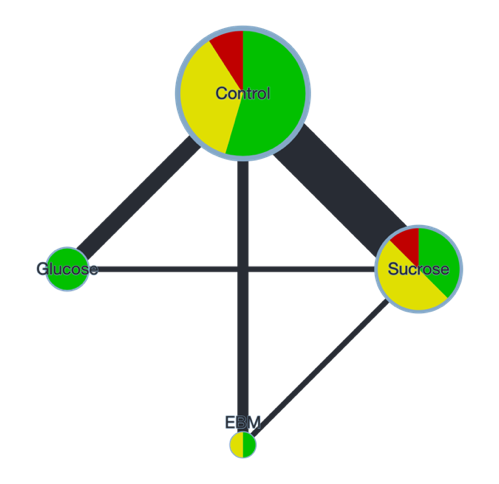


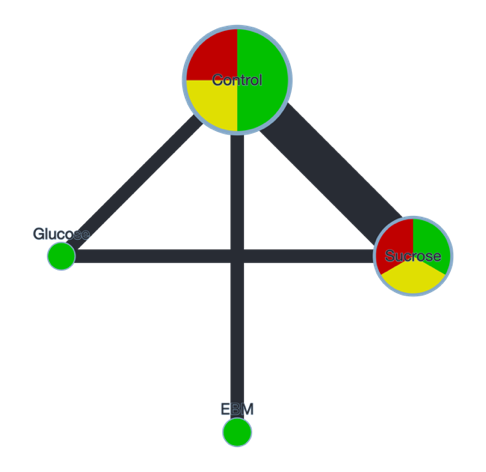

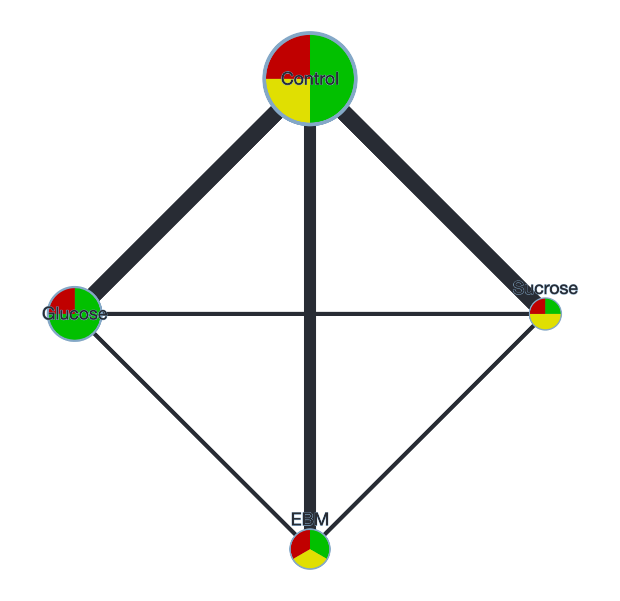


1

2

3

B. Network geometry for heart rate in (1) reactivity, (2) regulation and (3) recovery phase


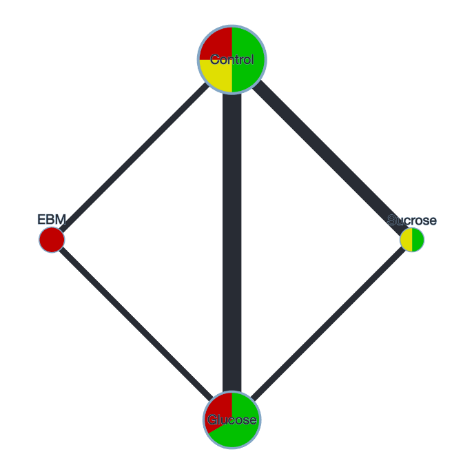

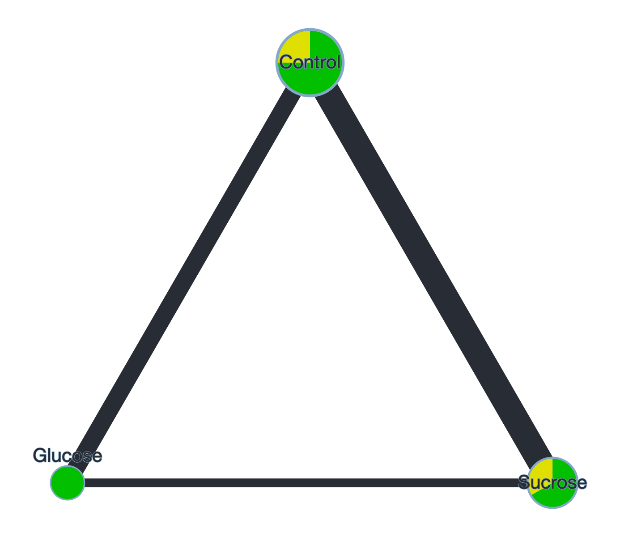


2

1

C. Network geometry for respiratory rate in (1) reactivity, and (2) recovery phase


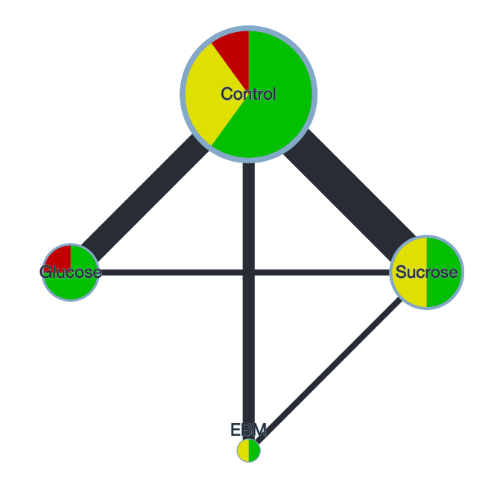

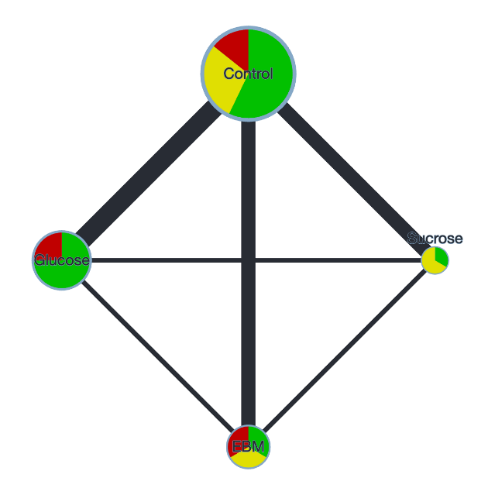

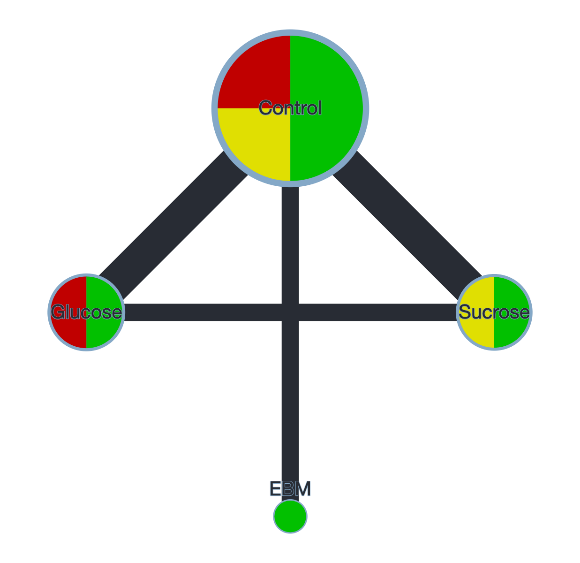


1

2

3

D. Network geometry for oxygen saturation in (1) reactivity, (2) regulation and (3) recovery phase


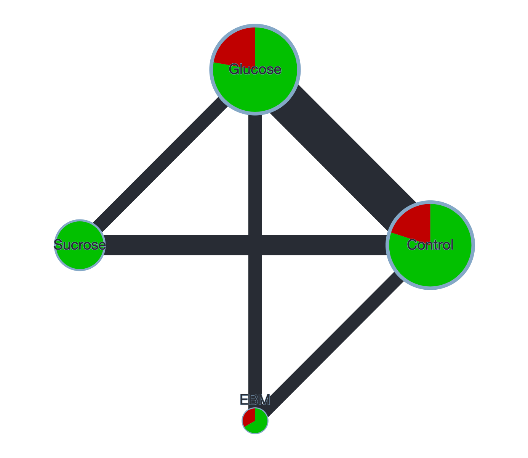


E. Network geometry of adverse effect

**eFigure 4. Forest plot of direct comparison**


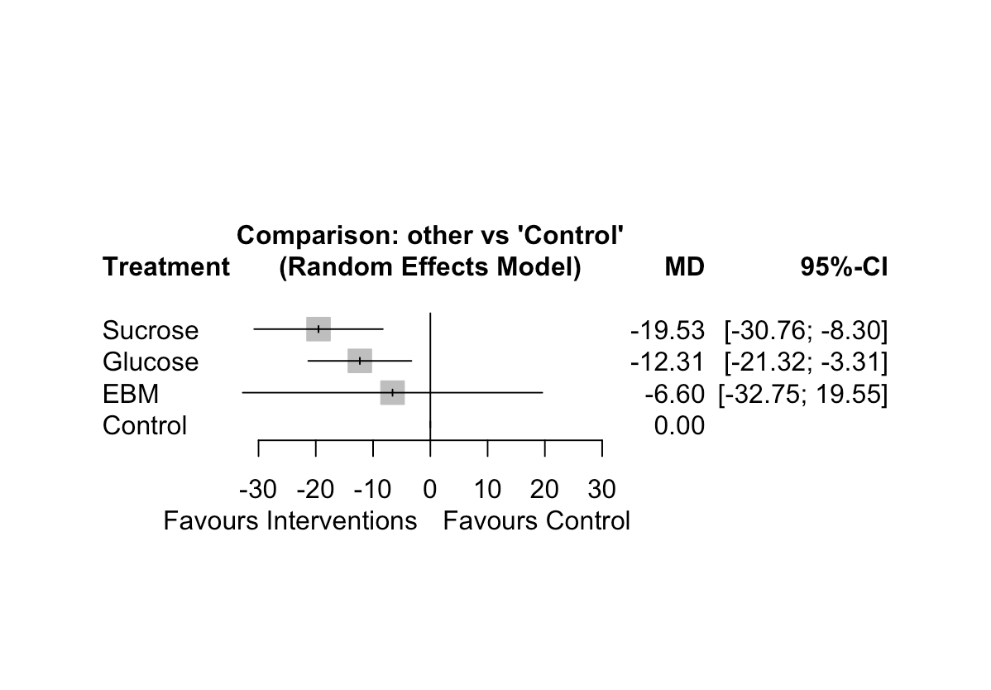


**A. Forest plot of overall crying time.** Control group as common comparator.

1

Forest plot of heart rate in reactivity phase


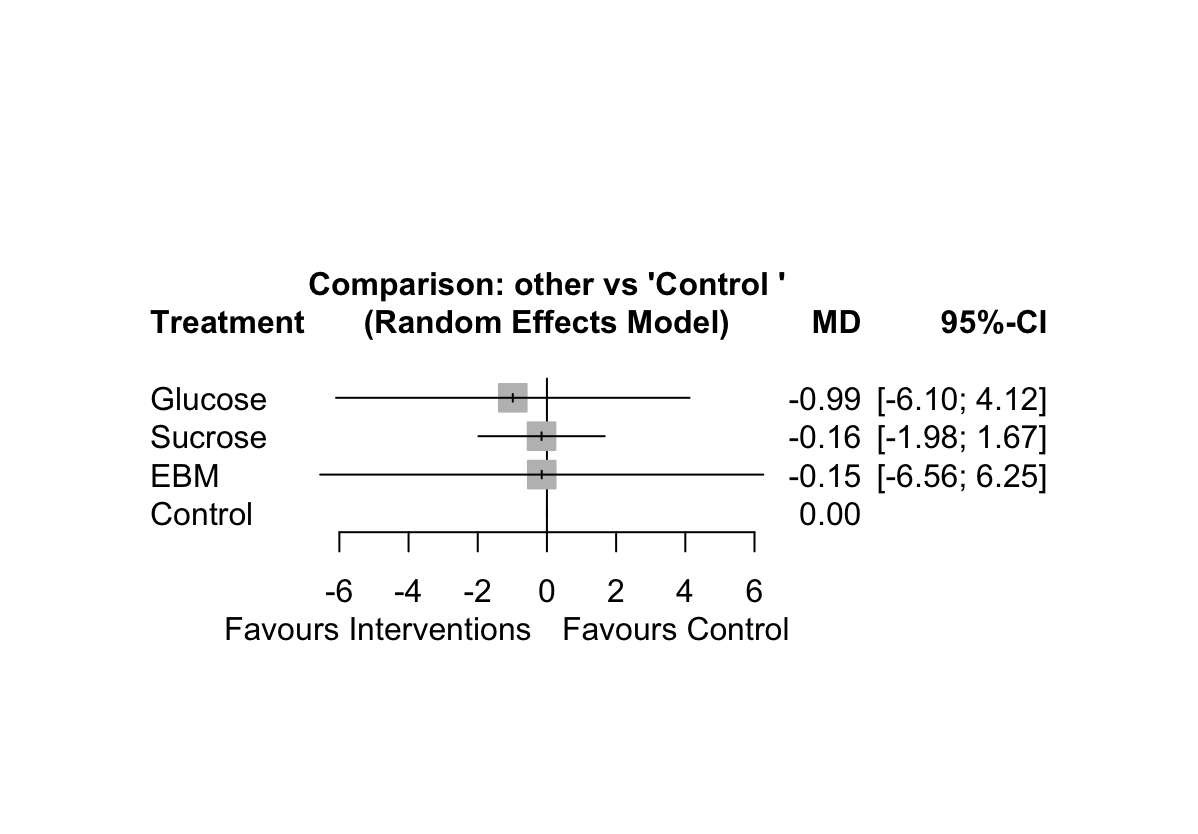


2

Forest plot of heart rate in regulation state

**
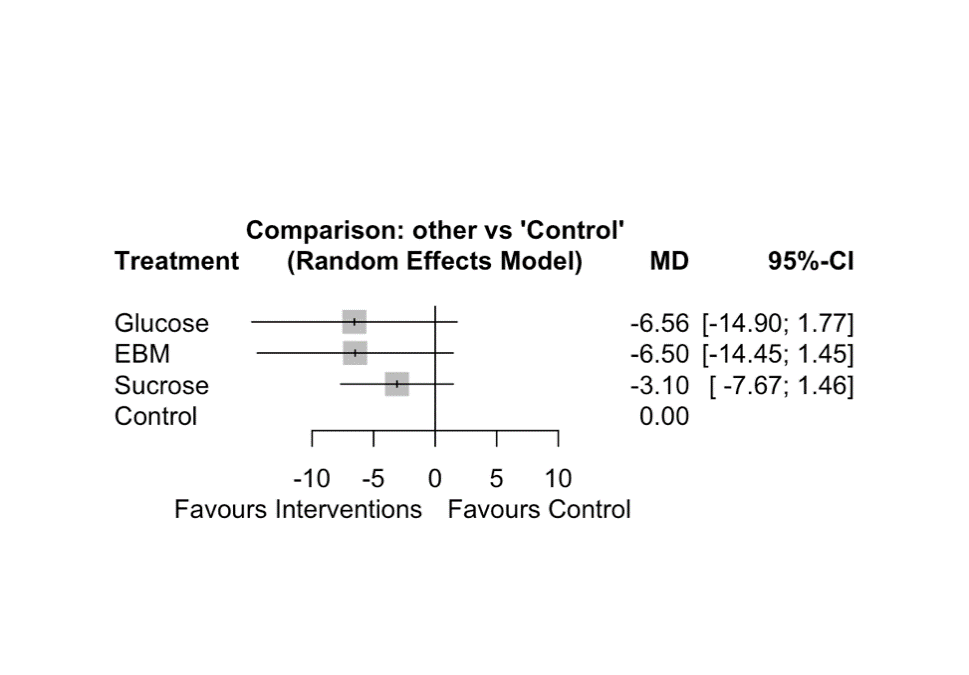
**

3

Forest plot of heart rate in recovery state


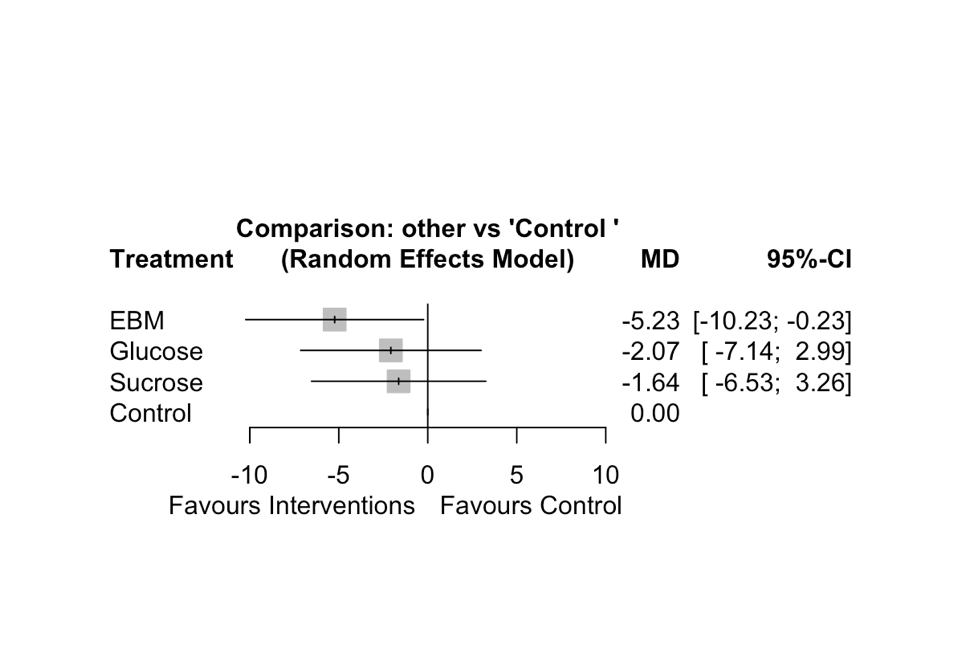


**B. Forest plot of heart rate:** (1) reactivity phase; (2) regulation phase; (3) recovery phase. Control group as common comparator.

**1.** Forest plot of oxygen saturation during reactivity state


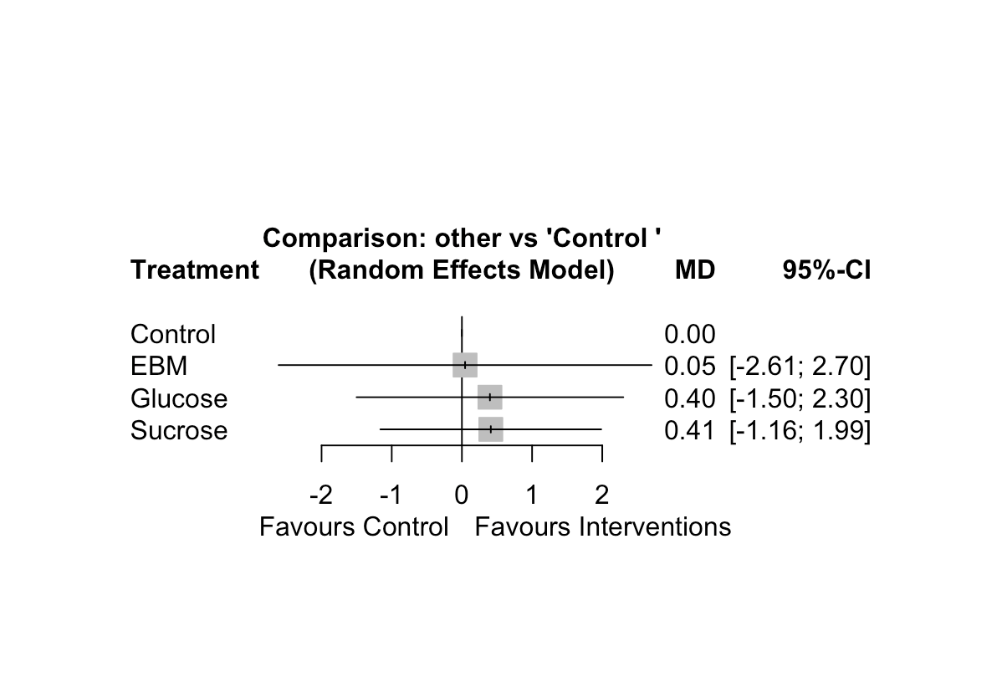


**2.** Forest plot of oxygen saturation regulation


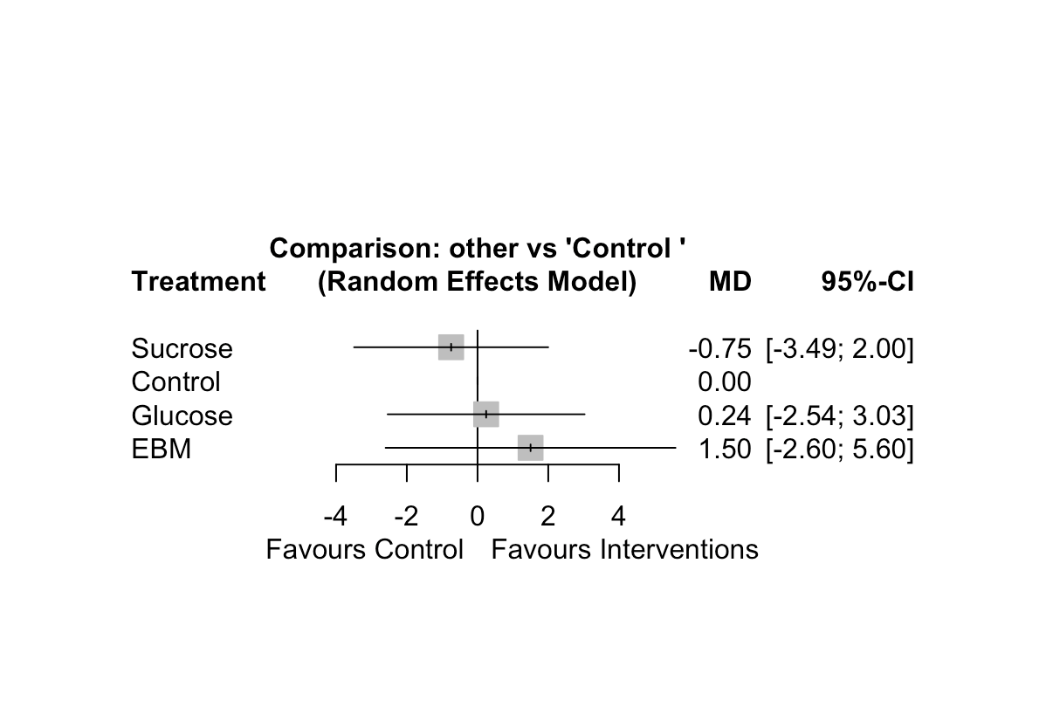


**3.** Forest plot of oxygen saturation recovery

**
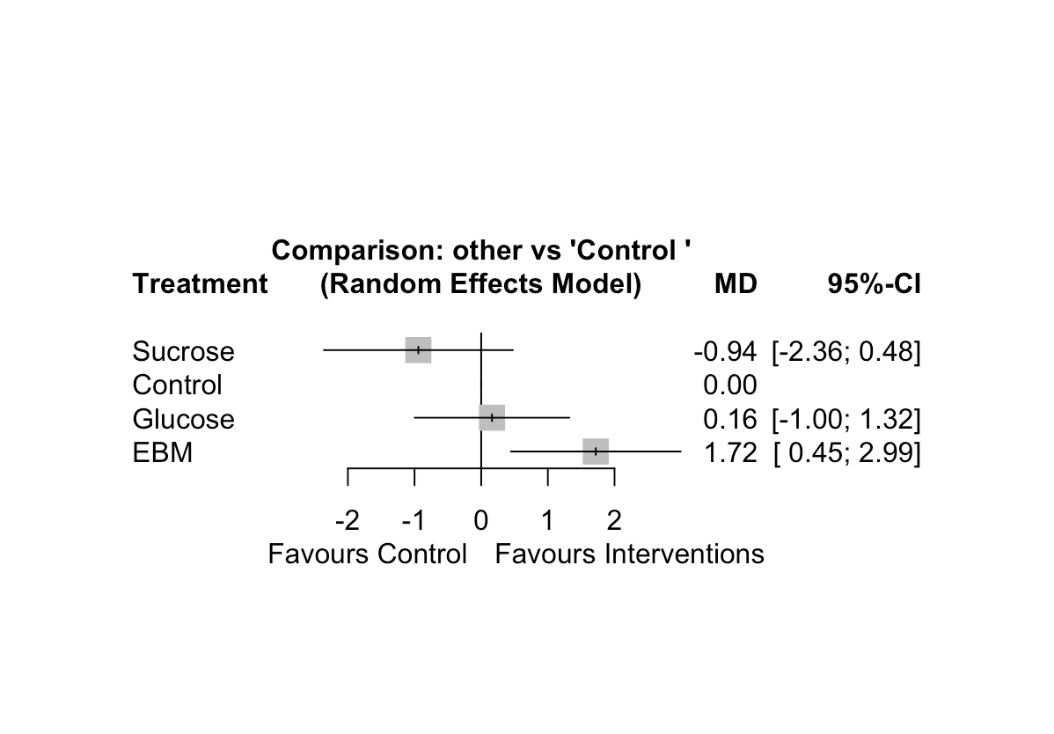
**

**C. Forest plot of oxygen saturation** among sweet solutions to control in: (1) reactivity phase; (2) regulation phase; and (3) recovery

1. Forest plot of respiratory rate during reactivity


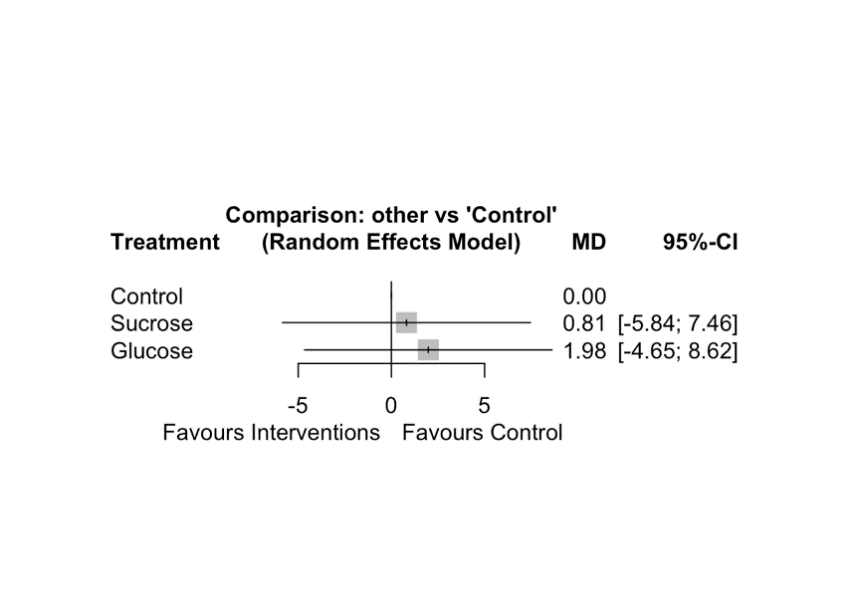


2. Forest plot of respiratory rate for recovery state


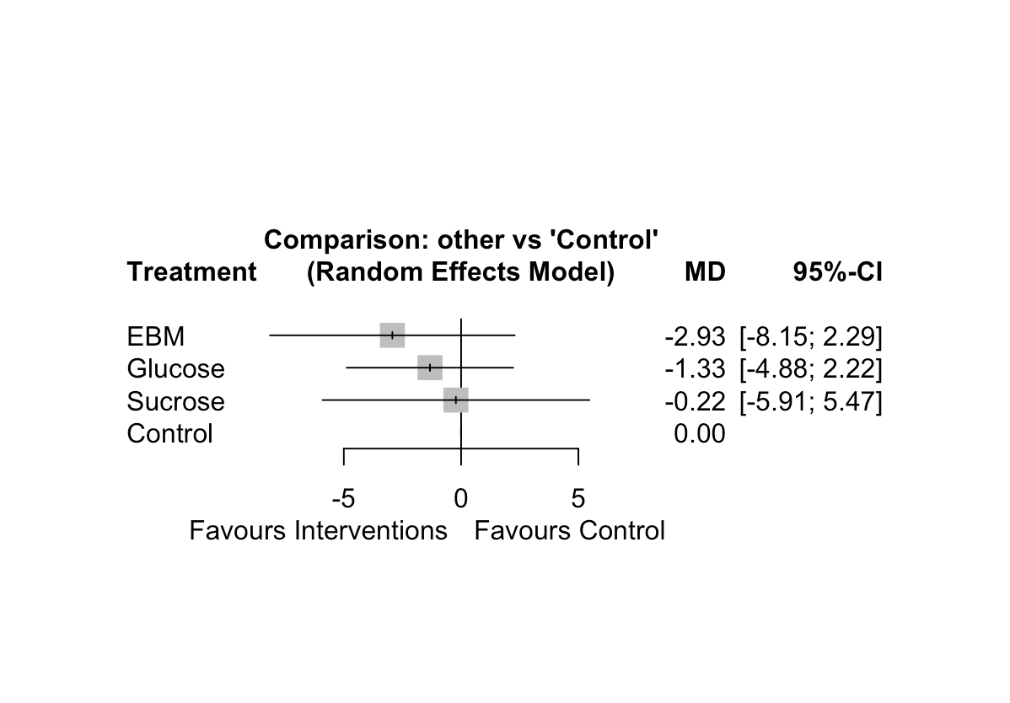


**D. Forest plot for respiratory rate** among sweet solutions to control in: (1) reactivity phase; and (2) recovery

**
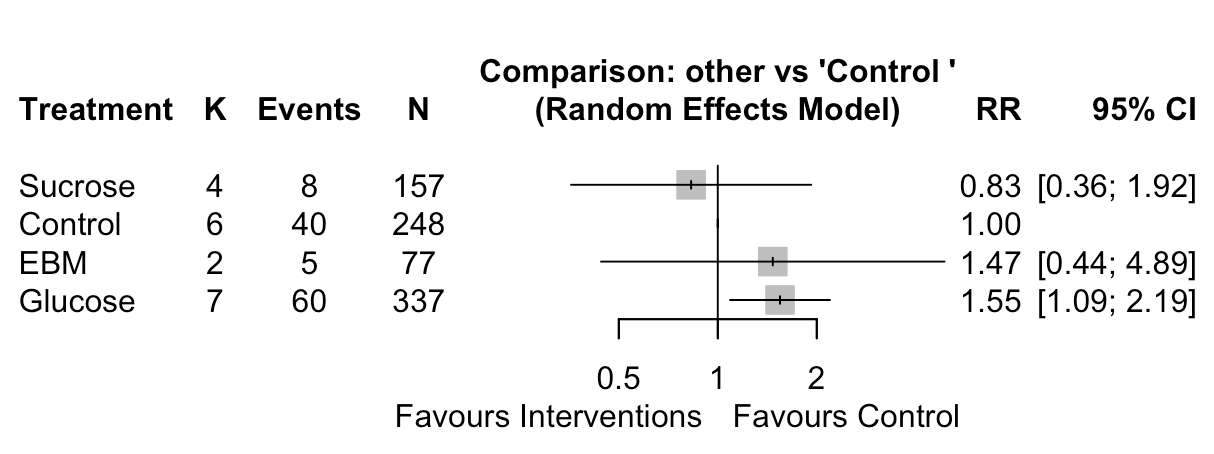
**

Note: K: total studies; N: total sample size; RR: risk ratio; CI: confidence interval

**E. Forest plot** of adverse event

**eFigure 5. Forward plot of Cook’s distance for model in the pain during phase**


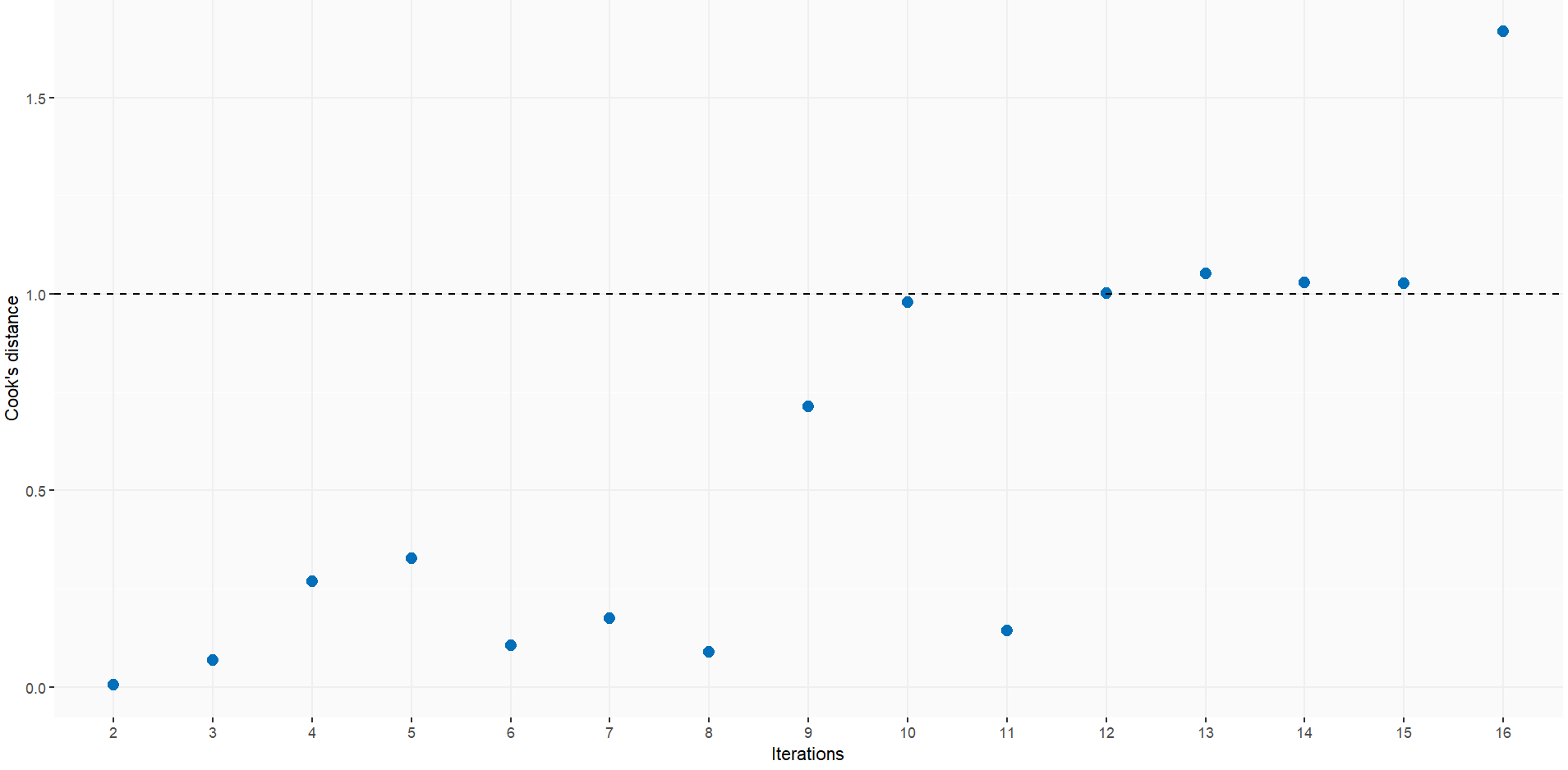


**eFigure 6. Forward plot of ratio of variance for model in the pain during phase**


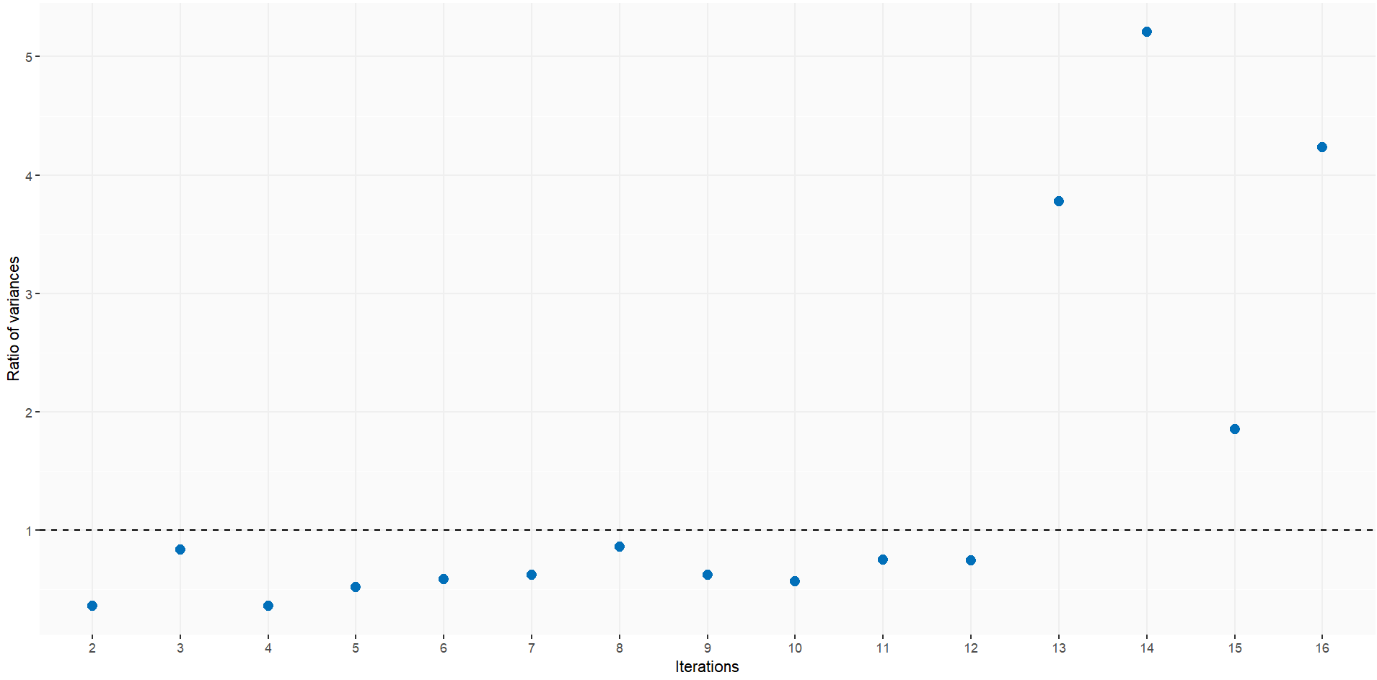


**eFigure 7. Forward plot of z-values that compare relative treatment effects estimated from direct and indirect evidence**


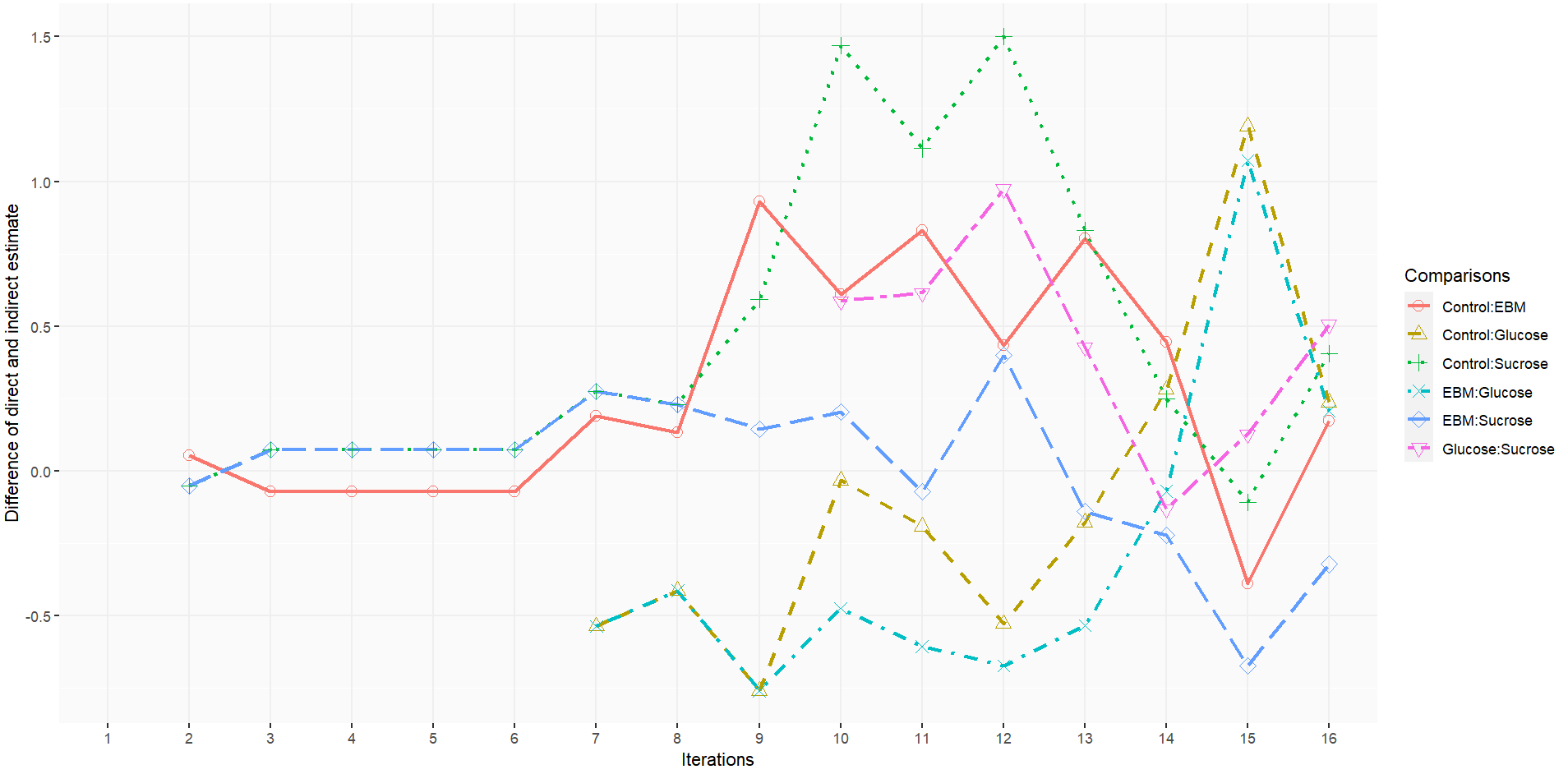


**eFigure 8. Forward plot for P-score in the pain during phase**


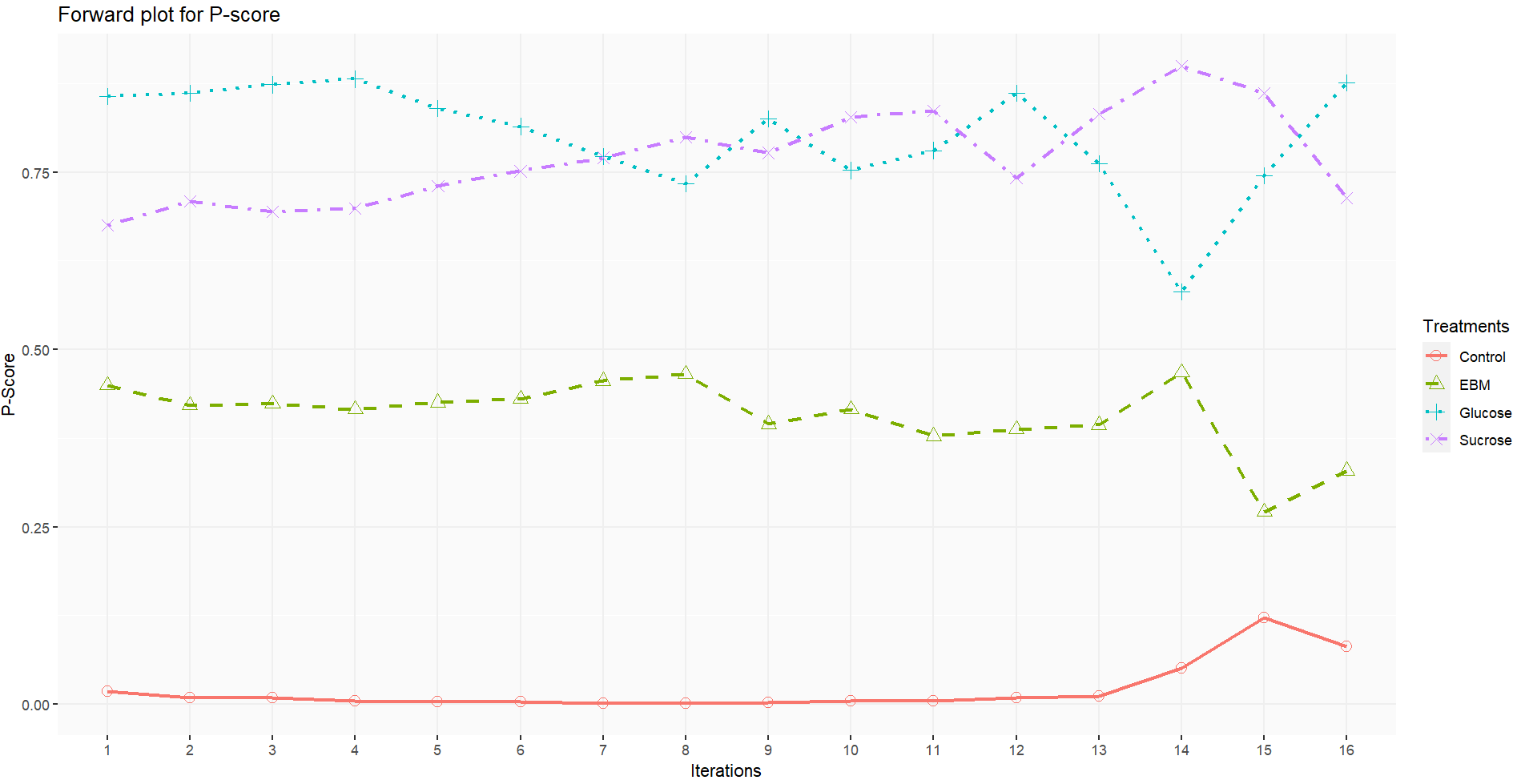


**eFigure 9. Funnel plot for publication bias**

**A.** Publication bias of pain level


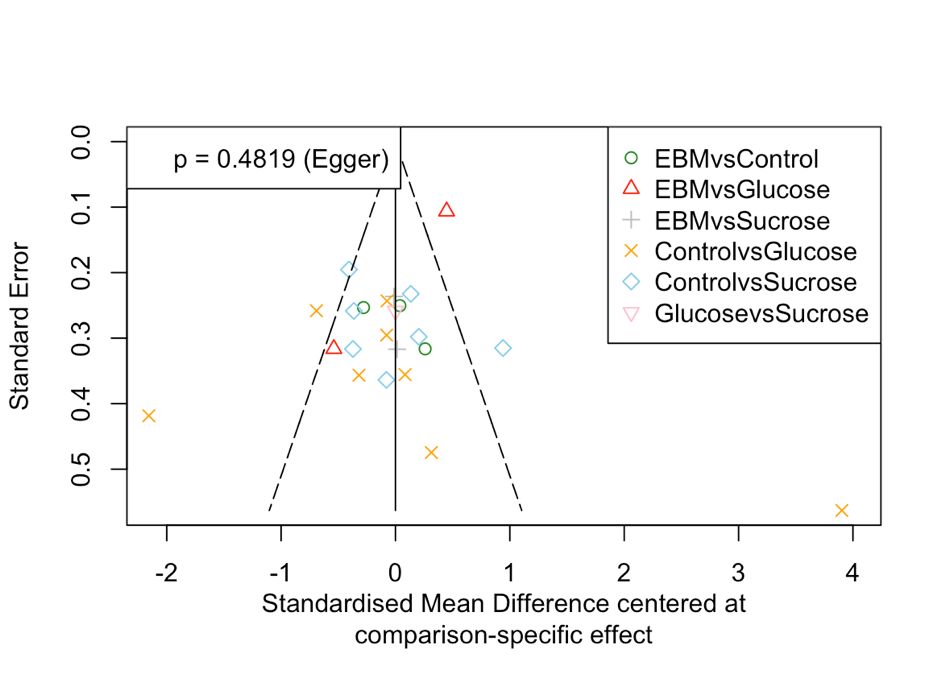


**(1)** Publication bias of pain in reactivity phase


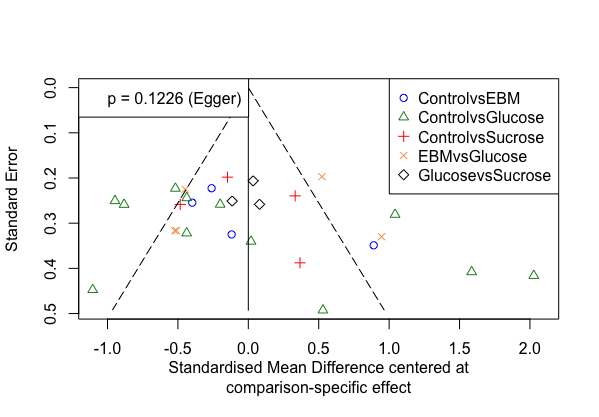


**(2)** Publication bias of pain level in regulation phase


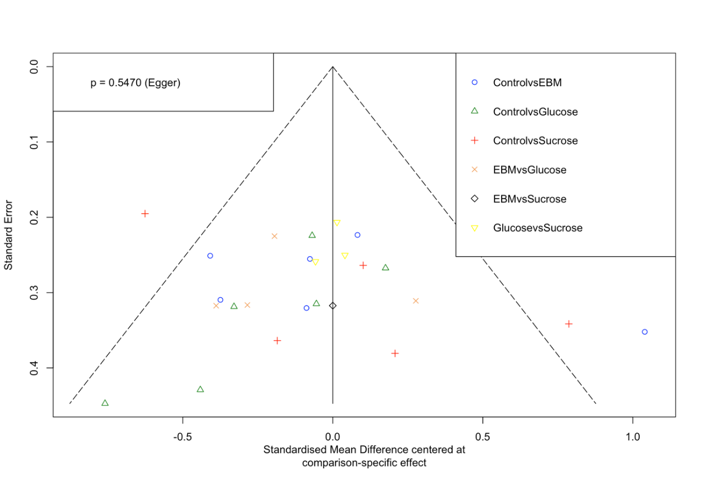


**(3)** Publication bias of pain in recovery phase

**B**. Publication bias of total crying time


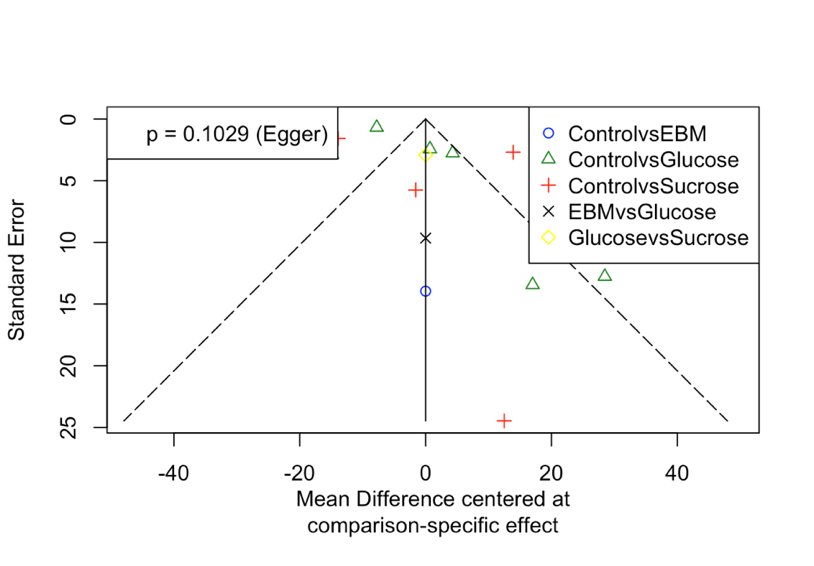


Publication bias of overall crying time

**C.** Publication bias of heart rate


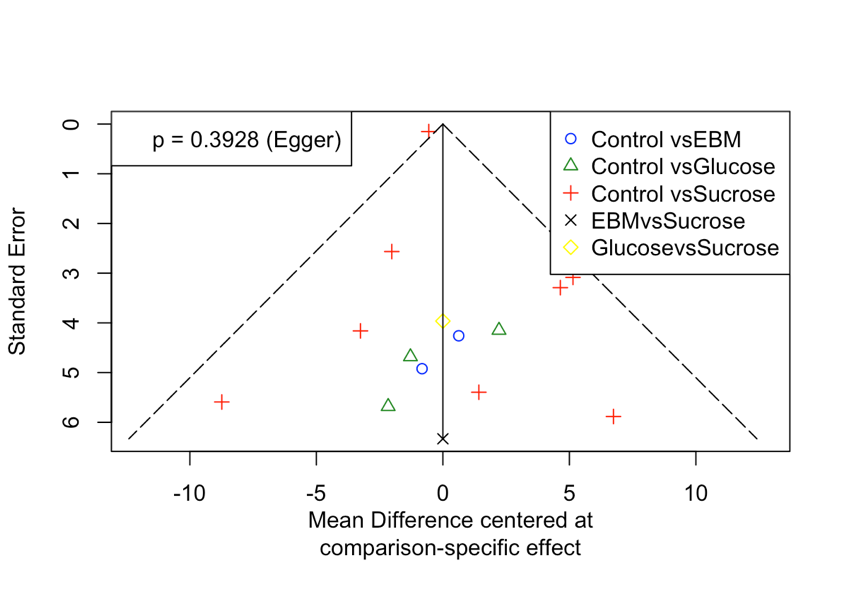


**(1)** Publication bias of heart rate during reactivity stage


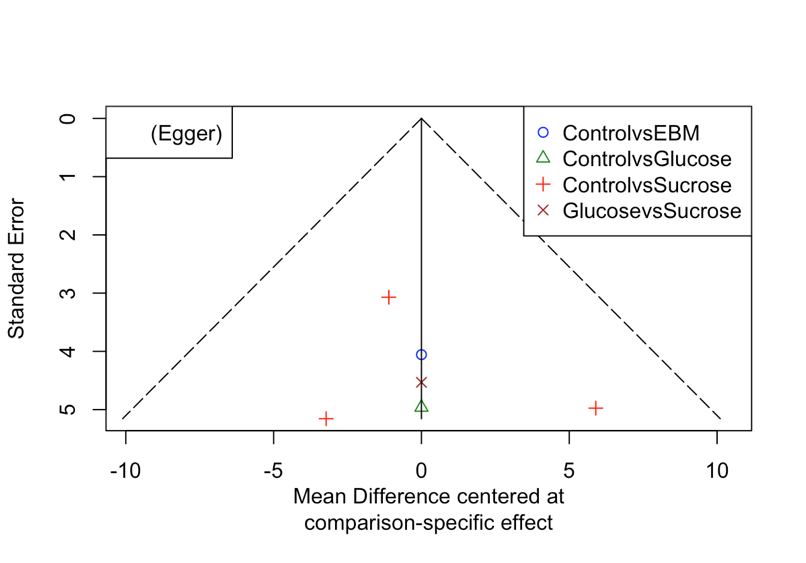


**(2)** Publication bias of heart rate in regulation stage (*p* Egger = 0.555)


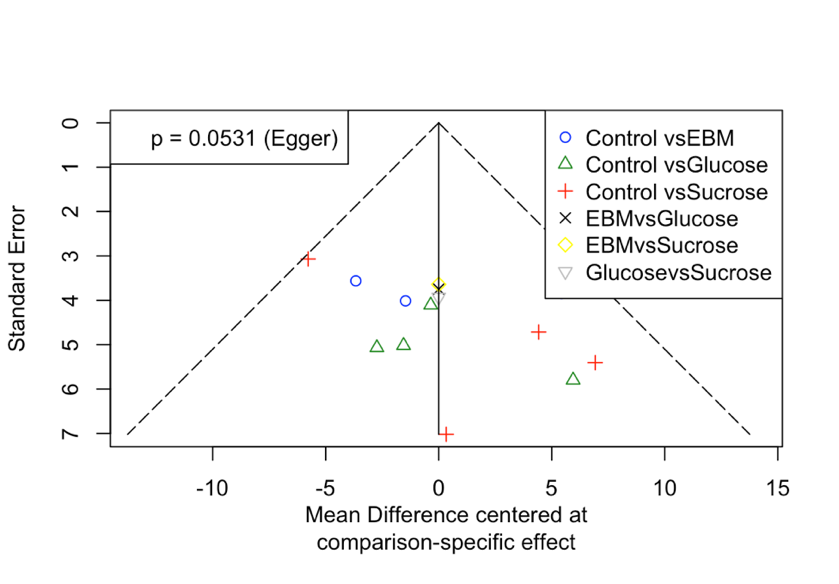


**(3)** Publication bias of heart rate in recovery stage

**D.** Publication bias of respiratory rate


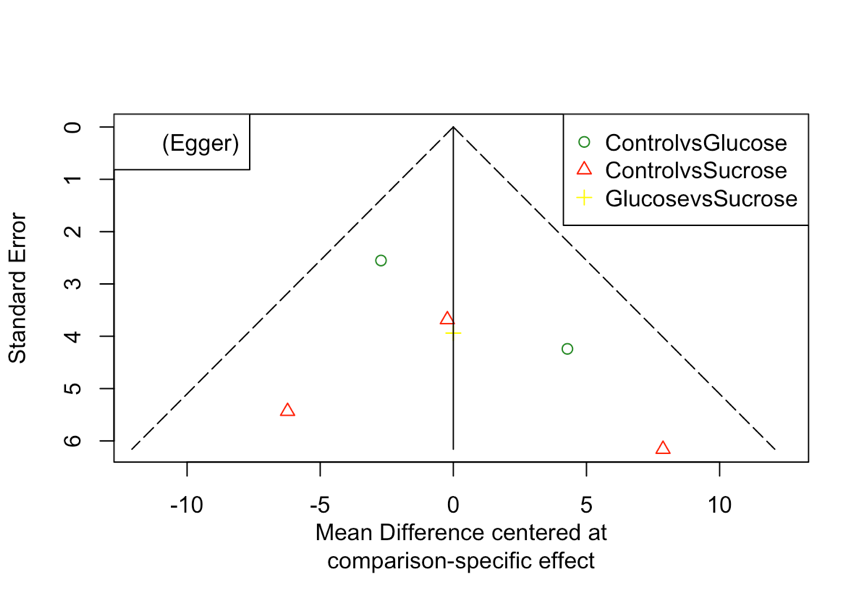


**(1)** Publication bias of respiratory rate in reactivity phase (*p* Egger = 0.334)


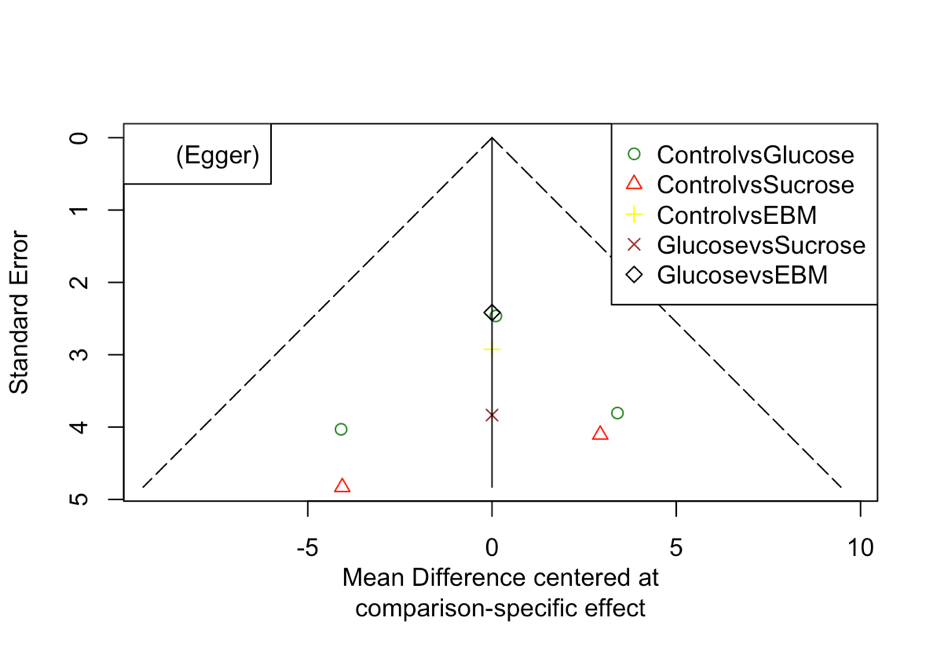


**(2)** Publication bias of respiratory rate recovery (*p* Egger = 0.681)

**E.** Publication bias of oxygen saturation


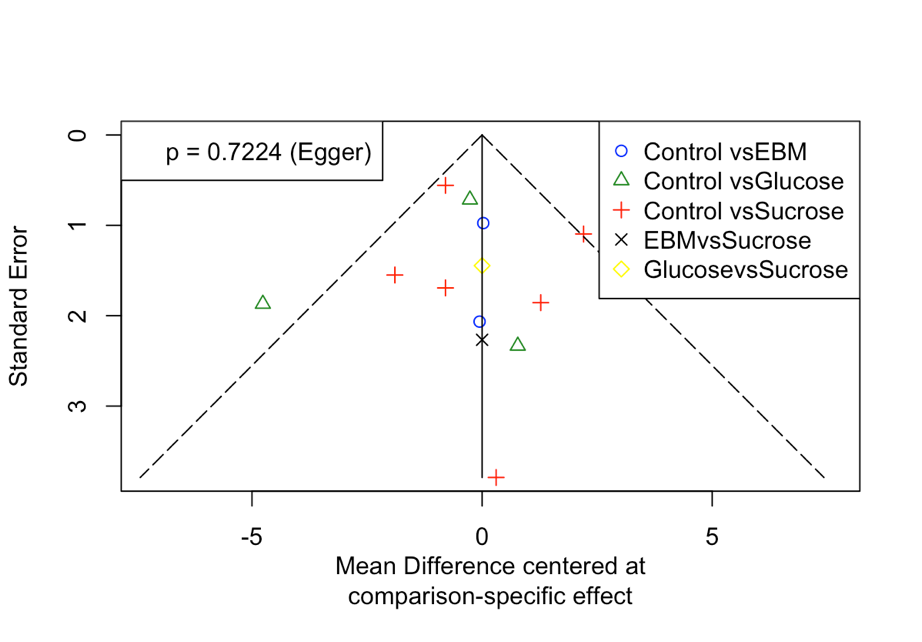


**(1)** Publication bias of oxygen saturation in reactivity phase


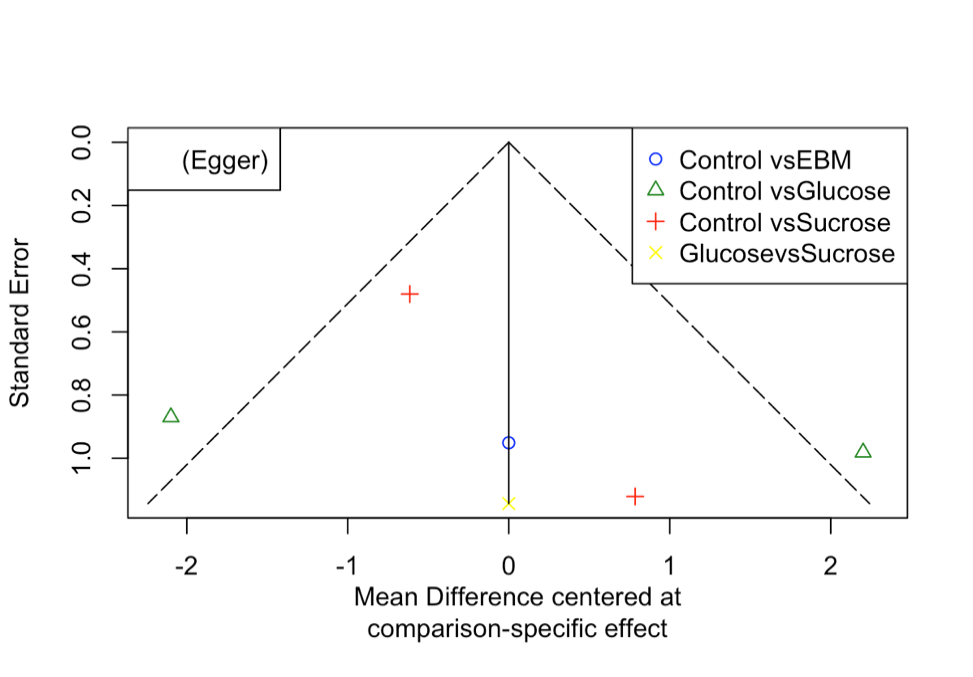


**(2)** Publication bias of oxygen saturation in regulation phase (*p* Egger = 0.424)


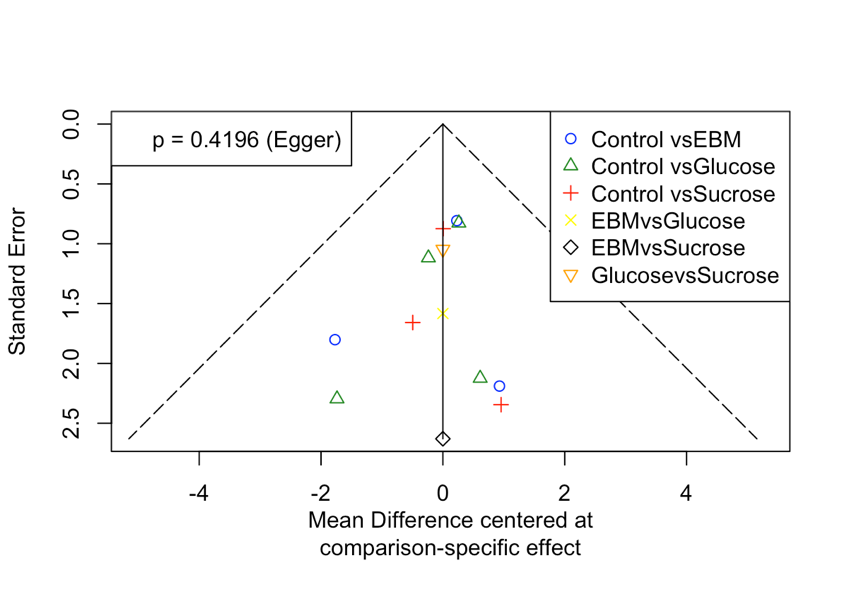


**(3)** Publication bias of oxygen saturation in recovery phase

**F.** Publication bias of adverse effect


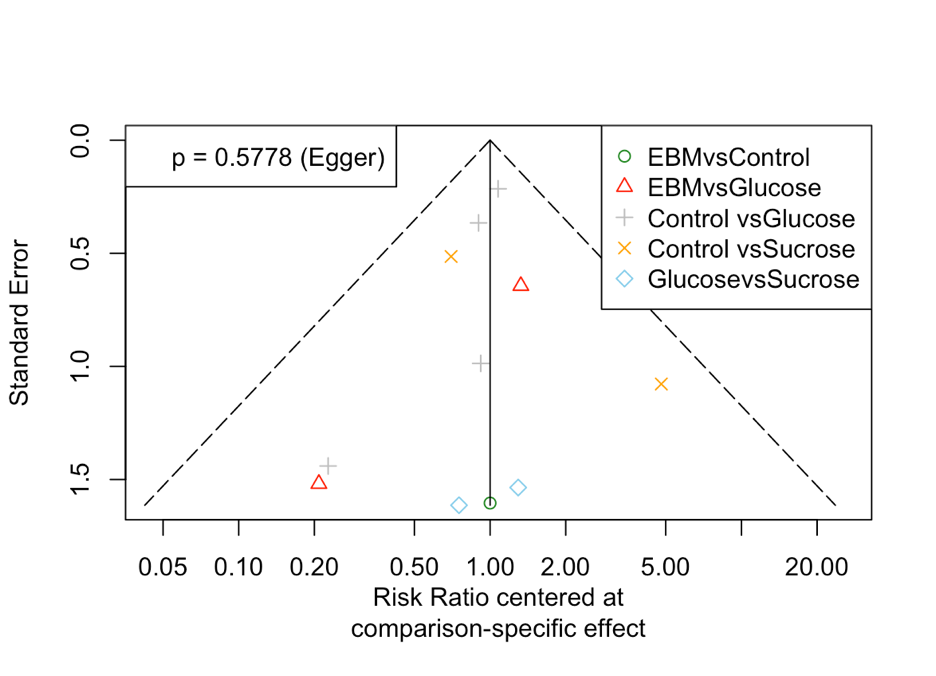


**(1)** Publication bias of adverse effect

**eFigure 10. Forest plots of differences in pain during reaction phase by risk of bias**

**A.** Forest plot based on evidence with high risk of bias


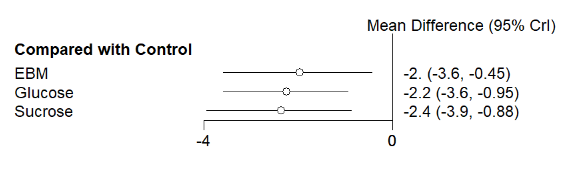


**B.** Forest plot based on evidence with non-high risk of bias


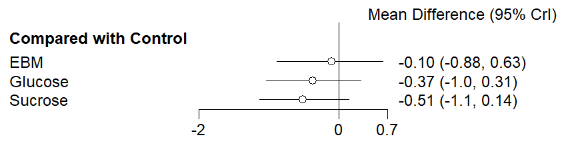


Note: The mean difference is standardized mean difference. CrI, credible interval.

**eFigure 11. Forest plots of differences in pain during reaction phase by painful procedure**

**A.** Forest plot based on evidence from heel stick


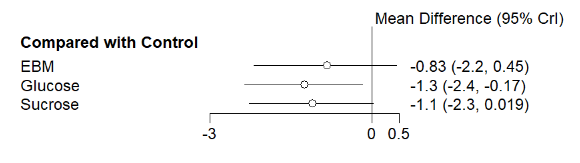


**B.** Forest plot based on evidence from non-heel stick


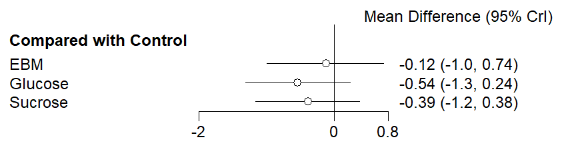


Note: The mean difference is standardized mean difference. CrI, credible interval.

**eFigure 12. Forest plots of adverse events during reaction phase using the inverse variance, peto and exact methods**

**A. Forest plot of the Peto methods**

**
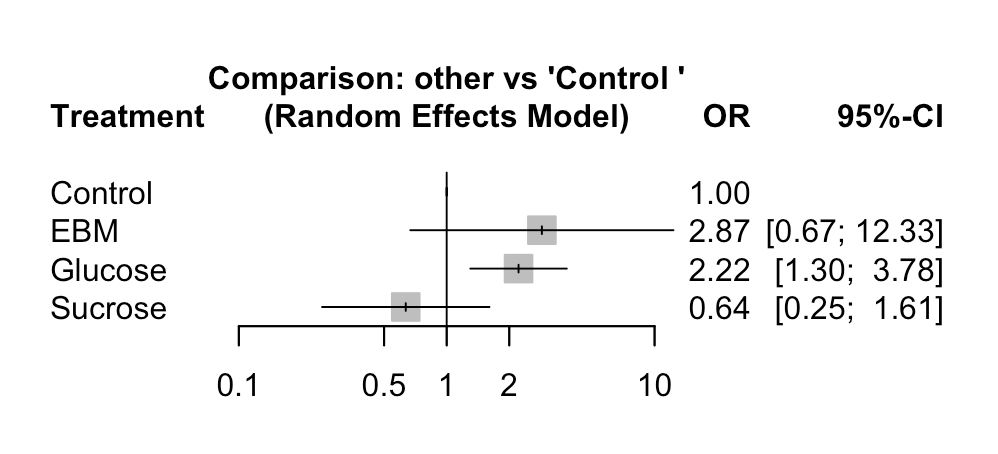
**

Note: OR = odd ratio

**B. Forest plot of the Inverse variance methods**

**
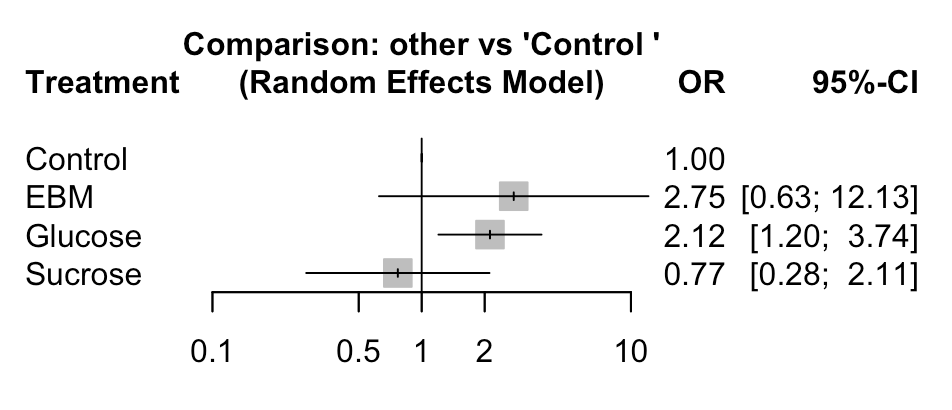
**

Note: OR = odd ratio

**C. Forest plot of the Exact methods**

**
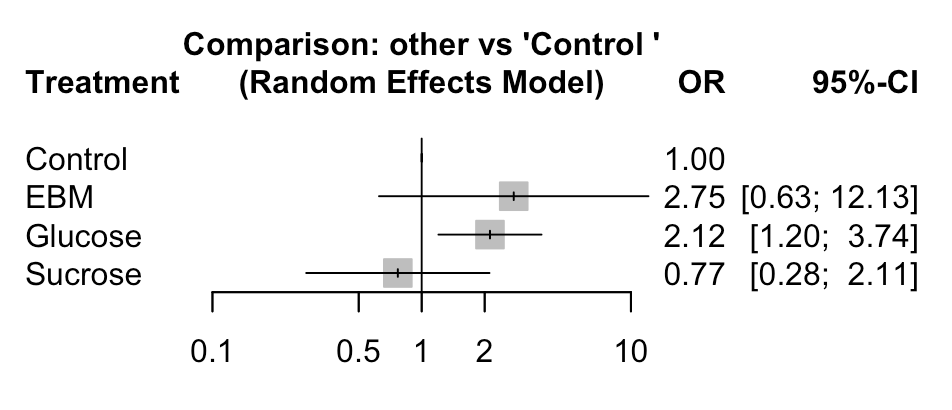
**

Note: OR = odd ratio

**References:**

1. Marilyn B, Stevens B, McAllister M, Dionne K, Jack A. Validation of the Premature Infant Pain Profile in the Clinical Setting. *The Clinical Journal of Pain*. 1999;15:297-303.

2. Carbajal R, Paupe A, Hoenn E, Lenclen R, Olivier-Martin M. DAN : une échelle comportementale d'Evaluation de la douleur aigui~ du nouveau-né. *Arch Pediatr*. 1997;4:623-628.

3. Lawrence J, Alcock D, Kay J, McGrath PJ. The development of a tool to assess neonatal pain. *Journal of Pain and Symptom Management*. 1991;6(3)doi:<https://doi.org/10.1016/0885-3924(91)91127-U>

4. Grunau RVE, Craig KD. Pain expression in neonates: facial action and cry. *Pain*. 1987;28(3)doi:doi:10.1016/0304-3959(87)90073-X

5. Hummel P, Puchalski M, Creech SD, Weiss MG. Clinical reliability and validity of the N-PASS: neonatal pain, agitation and sedation scale with prolonged pain. *J Perinatol*. Jan 2008;28(1):55-60. doi:10.1038/sj.jp.7211861

6. Abad F, Diaz N, Domenech E, Robayna M, Rico J. Oral sweet solution reduces pain-related behaviour in preterm infants. *Acta Pediatric*. 1996;85

7. Acharya AB, Annamali S, Taub NA, Field D. Oral sucrose analgesia for preterm infant venepuncture. *Arch Dis Child Fetal Neonatal*. 2004;89

8. Axelin A, ̈ SS, Kirjavainen J, Lehtonen L. Oral Glucose and Parental Holding Preferable to Opioid in Pain Management in Preterm Infants. *Clin J Pain*. 2009;25(2)

9. Bellieni CV, Cordelli DM, Perrone S, Nenci A, Buonocore G. Analgesia for premature children. *The Italian Journal of Pediatrics*. 2003;29

10. Bellieni CV, Buonocore G, Nenci A, Franci N, Cordelli DM, Bagnoli F. Sensorial Saturation: An Effective Analgesic Tool for Heel-Prick in Preterm Infants. *Biol Neonate*. 2001;80:15-18.

11. Boyer K, Johnston C, Walker CD, Filion F, Sherrard A. Does sucrose analgesia promote physiologic stability in preterm neonates? *Biol Neonate*. 2004;85(1):26-31. doi:10.1159/000074954

12. Boyle EM, Freer Y, Khan-Orakzai Z, et al. Sucrose and non-nutritive sucking for the relief of pain in screening for retinopathy of prematurity: a randomised controlled trial. *Archives of Disease in Childhood - Fetal and Neonatal Edition*. 2006;91(3):F166-F168. doi:10.1136/adc.2005.087668

13. Bucher H-U, Moser T, Seibenthal KV, Keel M, Wolf M, Duc G. Sucrose reduces pain reaction to heel lancing in preterm infants: a placebo-controlled, randomized and masked study. *Pediatric Research*. 1995;38(3)

14. Bueno M, Stevens B, de Camargo PP, Toma E, Krebs VL, Kimura AF. Breast milk and glucose for pain relief in preterm infants: a noninferiority randomized controlled trial. *Pediatrics*. Apr 2012;129(4):664-70. doi:10.1542/peds.2011-2024

15. Carbajal R, Lenclen R, Gajdos V, Jugie M, Paupe A. Crossover trial of analgesic efficacy of glucose and pacifier in very preterm neonates during subcutaneous injections. *Pediatrics*. Aug 2002;110(2 Pt 1):389-93. doi:10.1542/peds.110.2.389

16. Cirik VA, Efe E. The effect of expressed breast milk, swaddling and facilitated tucking methods in reducing the pain caused by orogastric tube insertion in preterm infants: A randomized controlled trial. *Int J Nurs Stud*. Apr 2020;104:103532. doi:10.1016/j.ijnurstu.2020.103532

17. Costa MC, Eckert GU, Fortes BG, Fortes Filho JB, Silveira RC, Procianoy RS. Oral glucose for pain relief during examination for retinopathy of prematurity: a masked randomized clinical trial. *Clinics (Sao Paulo)*. 2013;68(2):199-204. doi:10.6061/clinics/2013(02)oa13

18. Dehghani K, Ahmadabadi AB, Fallahzade H, Salimi T. Comparison of the Effect of Yakson Touch and Oral Glucose on the Severity of Phlebotomy Pain in Preterm Infants. *Iranian Journal of Neonatology*. 2019;10(4)doi:10.22038/ijn.2019.38769.1614

19. Desai S, Nanavati RN, Nathani R, Kabra N. Effect of Expressed Breast Milk versus Swaddling versus Oral Sucrose Administration on Pain Associated with Suctioning in Preterm Neonates on Assisted Ventilation: A Randomized Controlled Trial. *Indian J Palliat Care*. Oct-Dec 2017;23(4):372-378. doi:10.4103/IJPC.IJPC_84_17

20. Deshmukh LS, Udani RH. Analgesic Effect of Oral Glucose in Preterm Infants During Venipuncture—A Double-blind, Randomized, Controlled Trial. *Journal of Tropical Pediatrics*. 2002;48

21. Elserafy FA, Alsaedi SA, Louwrens J, Sadiq BB, Mersal AY. Oral sucrose and a pacifier for pain relief during simple procedures in preterm infants: a

randomized controlled trial. *Ann Saudi Med*. 2009;29(3)

22. de Sousa Freire NB, Garcia JBS, Lamy ZC. Evaluation of analgesic effect of skin-to-skin contact compared to oral glucose in preterm neonates. *Pain*. Sep 30 2008;139(1):28-33. doi:10.1016/j.pain.2008.02.031

23. Gaspardo CM, Miyase CI, Chimello JT, Martinez FE, Linhares MBM. Is pain relief equally efficacious and free of side effects with repeated doses of oral sucrose in preterm neonates? *Pain*. Jul 2008;137(1):16-25. doi:10.1016/j.pain.2007.07.032

24. Hsieh KH, Chen SJ, Tsao PC, et al. The analgesic effect of non-pharmacological interventions to reduce procedural pain in preterm neonates. *Pediatr Neonatol*. Feb 2018;59(1):71-76. doi:10.1016/j.pedneo.2017.02.001

25. Johnston CCl, Stremler RL, Stevens BJ, Horton LJ. Effectiveness of oral sucrose and simulated rocking on pain response in preterm neonates. *Pain*. 1997;72:1993-1999.

26. Johnston CC, Stremlera R, Horton L, Friedman A. Effect of repeated doses of sucrose during heel stick procedure in preterm neonates. *Biology of the Neonate*. 1999;75:160-166.

27. Kazmi AA, Noor M, Shabir S, Aziz S, Aslam S, Fatima A. Comparison of Mean Pain score in Preterm neonatal Procedural Pain Treated with Expressed Breast Milk and 25% Dextrose Solution. *PJMHS*. 2020;14(4)

28. Kristoffersen L, Skogvoll E, Hafstrom M. Pain reduction on insertion of a feeding tube in preterm infants: a randomized controlled trial. *Pediatrics*. Jun 2011;127(6):e1449-54. doi:10.1542/peds.2010-3438

29. Kumari S, Datta V, Rehan H. Comparison of the Efficacy of Oral 25% Glucose with Oral 24% Sucrose for Pain Relief during Heel Lance in Preterm Neonates: A Double Blind Randomized Controlled Trial. *J Trop Pediatr*. Feb 2017;63(1):30-35. doi:10.1093/tropej/fmw045

30. McCullough S, Halton T, Mowbray D, Macfarlane PI. Lingual sucrose reduces the pain response to nasogastric tube insertion: a randomised clinical trial. *Arch Dis Child Fetal Neonatal Ed*. Mar 2008;93(2):F100-3. doi:10.1136/adc.2006.110338

31. D'Lima A, Naik A, Sreekumar K, Silveira M. Efficacy of expressed breast milk alone or in combination with paracetamol in reducing pain during ROP screening: A randomized controlled trial. *Journal of Clinical Neonatology*. 2021;10(2)doi:10.4103/jcn.jcn_198_20

32. Okan F, Coban A, Ince Z, Yapici Z, Can G. Analgesia in preterm newborns: the comparative effects of sucrose and glucose. *Eur J Pediatr*. Oct 2007;166(10):1017-24. doi:10.1007/s00431-006-0373-z

33. Olsson E, Eriksson M. Oral glucose for pain relief during eye examinations for retinopathy of prematurity. *J Clin Nurs*. Apr 2011;20(7-8):1054-9. doi:10.1111/j.1365-2702.2010.03529.x

34. Ou-Yang MC, Chen IL, Chen CC, Chung MY, Chen FS, Huang HC. Expressed breast milk for procedural pain in preterm neonates: a randomized, double-blind, placebo-controlled trial. *Acta Paediatr*. Jan 2013;102(1):15-21. doi:10.1111/apa.12045

35. Pandey M, Datta V, Rehan HS. Role of sucrose in reducing painful response to orogastric tube insertion in preterm neonates. *Indian J Pediatr*. Jun 2013;80(6):476-82. doi:10.1007/s12098-012-0924-4

36. Ramar P, Vinayagam P, Seeralar A. Effectiveness of oral glucose as analgesic for neonates undergoing retinopathy of prematurity screening – A randomized pilot study for a parallel randomized control trial. *Journal of Clinical Neonatology*. 2019;8(4)doi:10.4103/jcn.JCN_59_18

37. Ramenghi LA, Wood CM, Griffith GC, Levene MI. Reduction of pain response in premature infants using intraoral sucrose. *Archives of Disease in Childhood*. 1996;74

38. Ramenghi LA, Evans DJ, Levene MI. “Sucrose analgesia”: absorptive mechanism or taste perception? *Arch Dis Child Fetal Neonatal* 1999;80

39. Ranjbar A, Bernstein C, Shariat M, Ranjbar H. Comparison of facilitated tucking and oral dextrose in reducing the pain of heel stick in preterm infants: a randomized clinical trial. *BMC Pediatr*. Apr 14 2020;20(1):162. doi:10.1186/s12887-020-2020-7

40. Rawal S, Ghai A, Jindal T. Twenty-Five Percent Dextrose and EBM in Pain Relief During Heel Lance in Late Preterm Babies Using the PIPP Score: A Randomized Controlled Trial. *Journal of Neonatology*. 2018;32(2-3):43-49. doi:10.1177/0973217918795027

41. Rodrigues L, Nesargi SV, Fernandes M, Shashidhar A, Rao SPN, Bhat S. Analgesic Efficacy of Oral Dextrose and Breast Milk during Nasopharyngeal Suctioning of Preterm Infants on CPAP: A Blinded Randomized Controlled Trial. *J Trop Pediatr*. Dec 1 2017;63(6):483-488. doi:10.1093/tropej/fmx017

42. Sagheb S, Mosayebi Z, Karimi N, Nikseresht Z, Ani MM. Efficacy of 25% glucose in pain alleviation during retinopathy of prematurity

(ROP) screening: A randomized controlled trial. *Acta Medica Iran*. 2020;58(12)

43. Sasidharan R, Gupta N, Yadav B, Chawla D, Singh K, Kumarendu Singh A. 25% Dextrose Versus 24% Sucrose for Heel Lancing in Preterm Infants: A Noninferiority RCT. *Pediatrics*. May 1 2022;149(5)doi:10.1542/peds.2021-054618

44. Sener Taplak A, Erdem E. A Comparison of Breast Milk and Sucrose in Reducing Neonatal Pain During Eye Exam for Retinopathy of Prematurity. *Breastfeed Med*. Jun 2017;12:305-310. doi:10.1089/bfm.2016.0122

45. Tekgunduz KS, Polat S, Gurol A, Apay SE. Oral Glucose and Listening to Lullaby to Decrease Pain in Preterm Infants Supported with NCPAP: A Randomized Controlled Trial. *Pain Manag Nurs*. Feb 2019;20(1):54-61. doi:10.1016/j.pmn.2018.04.008

46. Uzelli D, Yapucu Gunes U. Oral glucose solution to alleviate pain induced by intramuscular injections in preterm infants. *J Spec Pediatr Nurs*. Jan 2015;20(1):29-35. doi:10.1111/jspn.12094

47. Vezyroglou K, Mehler K, Kribs A, et al. Oral glucose in preterm neonates during oropharyngeal suctioning: a randomized controlled cross-over trial. *Eur J Pediatr*. Jul 2015;174(7):867-74. doi:10.1007/s00431-014-2472-6

48. Banga S, Datta V, Rehan HS, Bhakhri BK. Effect of Sucrose Analgesia, for Repeated Painful Procedures, on Short-term Neurobehavioral Outcome of Preterm Neonates: A Randomized Controlled Trial. *J Trop Pediatr*. Apr 2016;62(2):101-6. doi:10.1093/tropej/fmv079

49. Dilli D, İlarslan NE, Kabataş EU, Zenciroğlu A, Şimşek Y, Okumuş N. Oral sucrose and non-nutritive sucking goes some way to reducing pain during retinopathy of prematurity eye examinations. *Acta Paediatr*. Feb 2014;103(2):e76-9. doi:10.1111/apa.12454

50. Grabska J, Walden P, Lerer T, et al. Can oral sucrose reduce the pain and distress associated with screening for retinopathy of prematurity? *J Perinatol*. Jan 2005;25(1):33-5. doi:10.1038/sj.jp.7211199

51. Ilarslan NC, Dilli D, Kabataş E, et al. Predicting The Efficacy of Oral Sucrose in Reducing Pain During Ophtalmological Examination for Retinopathy of Prematurity: A Prospective Randomised Study. *Archives of Disease in Childhood*. 2012;97(Suppl 2):A55-A55. doi:10.1136/archdischild-2012-302724.0190

52. Silveira A, Christoffel MM, Velarde LGC, Rodrigues EDC, Magesti BN, Souza RO. Effect of glucose and non-nutritive sucking on puncture pain in premature infants: a crossover clinical trial. *Rev Esc Enferm USP*. 2021;55:e03732. Efeito da glicose e sucção não nutritiva na dor de prematuros na punção: ensaio clínico crossover. doi:10.1590/s1980-220x2020018303732

53. Velumula PK, Elbakoush F, Tabb C, 2nd, et al. Breast milk vs 24% sucrose for procedural pain relief in preterm neonates: a non-inferiority randomized controlled trial. *J Perinatol*. Jul 2022;42(7):914-919. doi:10.1038/s41372-022-01352-2
